# From *in silico* prediction to experimental validation: Identification of drugs and novel synergistic combinations that inhibit growth of inflammatory breast cancer cells

**DOI:** 10.1371/journal.pone.0339543

**Published:** 2026-09-16

**Authors:** Esraa A. Salim, Xiaojia Ji, Michael Tarpley, Maria S. Dixon, Weifan Zheng, John E. Scott, Kevin P. Williams

**Affiliations:** 1 Biomanufacturing Research Institute and Technology Enterprise, North Carolina Central University, Durham, North Carolina, United States of America; 2 Department of Pharmaceutical Sciences, North Carolina Central University, Durham, North Carolina, United States of America; BMSCE: BMS College of Engineering, INDIA

## Abstract

Drug repurposing can accelerate the identification of novel therapeutic candidates for rare cancers such as inflammatory breast cancer (IBC), an aggressive type with limited therapeutic options. Here, we report an experimental validation study of compounds previously identified through two computational approaches: Literature Wide Association Studies (LWAS) and Gene Reversal Rate (GRR). Candidate compounds were tested using orthogonal cell viability assays in 2D models across IBC and non-IBC cell lines. In the SUM149 IBC cell line, repurposed compounds predicted from LWAS achieved a 70% success rate, with several showing nanomolar potency, while those predicted from GRR showed a 38% success rate. Through systematic combination screening in both 2D and 3D-spheroid SUM149 models, we identified novel synergistic compound pairs targeting crosstalk between IGF-1R, EGFR and PI3K/Akt/mTOR pathways, with high synergy scores across multiple reference models. Using these combinations, western blott analysis revealed significant suppression in the phosphorylation of key signaling proteins and downstream effectors, while wound healing assays showed reduced cell migration with some combination treatments, suggesting effective pathway inhibition. To further validate these findings at the transcriptional level, RNA-Seq analysis in SUM149 cells confirmed that the GRR drug combinations significantly reversed the IBC gene expression signature (IBC-GES) and identified several clinically relevant genes whose expression was significantly altered. Together, these findings validate our computational predictions and identify candidate combination strategies that may help address therapeutic resistance in IBC. This integrated computational-experimental approach establishes a pipeline for systematic drug repurposing and highlights novel therapeutic combinations for further investigation.

## Introduction

Inflammatory breast cancer (IBC) is an uncommon but highly aggressive type of breast cancer [1–3]. In Western nations, IBC accounts for up to 5% of breast cancer cases [4, 5], with higher rates reported among African American [6] and North African populations, reaching 7–11.1% [7]. Although considered rare, IBC is responsible for 8–10% of all breast cancer deaths [8]. At diagnosis, up to 85% of IBC patients have lymph node involvement, and up to 30% show distant metastasis [9, 10]. The aggressive nature of IBC and the presence of unique characteristics in its presentation [11, 12] and progression contribute significantly to the higher rates of treatment resistance, relapse, and mortality [13]. Despite the widespread adoption of trimodality treatment for IBC [14], which combines anthracycline- and taxane-based neoadjuvant chemotherapy, surgery, and radiation therapy [15], the 5-year and 10-year overall survival (OS) rates remain low at 55.4% and 37.3%, respectively [16, 17]. Currently, there is no Food and Drug Administration (FDA)-approved targeted therapy specific to IBC and the current treatment strategies are primarily based on studies of high-risk non-IBC patients [18]. This critical gap in treatment options highlights the essential need for innovative therapeutic strategies to improve patient outcomes in rare diseases.

Several drug discovery approaches can be undertaken to find novel drugs for IBC, including drug repurposing, which is the application of existing, market-approved drugs to new disease indications, and offers significant advantages over *de novo* drug discovery [19]. This approach aims to enhance the drug discovery process by reducing both time and cost [20, 21]. By utilizing known drugs with established safety profiles, drug repurposing can bypass intermediate development steps and accelerate the path to clinical application [22–24]. The primary objective of drug repurposing is to identify novel associations between existing drugs and diseases through two main approaches: computational prediction and experimental validation [25]. Modern computational drug repurposing technologies seek to systematically analyze diverse data types, including genomic, proteomic, clinical health records, and scientific literature to predict potential drug-disease connections [21, 26, 27]. Our previous studies used two distinct computational approaches [28, 29] to identify repurposing candidates for IBC treatment. The first approach utilized Literature Wide Association Study (LWAS) [28], which applies extensive text mining [30] as a powerful tool to identify new repurposed candidates. The second approach used Gene Reversal Rate (GRR) [29], which employs gene expression reversal methodology [31, 32] to identify compounds that are capable of reversing cancer-associated gene expression patterns. Experimental validation then tests these predicted compounds in disease-relevant models and helps prioritize candidates for further development. This present study aimed to assess the efficacy of the drug and compound candidates we had predicted for IBC from our LWAS [28] and GRR [29] studies, using *in vitro* screening techniques in cell-based models. We tested these candidates both alone and in combination, using different cell viability and proliferation assays to evaluate drug potency and efficacy in 2D IBC and non-IBC breast cancer cell models and in 3D SUM149 spheroids. Our initial findings identified a subset of compounds with anti-proliferative activity in both IBC and non-IBC cell lines, with 70% (17 of 24) of the LWAS-predicted compounds and 38% (6 of 16) of the GRR-predicted compounds showing efficacy in the SUM149 IBC cell line. To address potential drug resistance to single-agent treatment, we used a matrix-based drug combination assay and synergy analysis pipeline to identify synergistic combinations. Three novel synergistic combinations were identified with significant effects on proliferation, spheroid integrity, protein expression, and cell migration in SUM149 IBC cell models. These combinations comprised compounds predicted by our GRR study and to our knowledge, had not been previously reported in IBC clinical trials or in published studies. In addition to the phenotypic and protein-level validation, we further assessed transcriptional responses to these GRR-derived combinations using RNA-Seq and observed a significant reversal of genes in the IBC gene expression signature. Overall, the synergistic GRR-derived combinations that we identified act on different targets including insulin-like growth factor 1 receptor (IGF-1R) and epidermal growth factor receptor (EGFR) signaling, as well as downstream PI3K/Akt/mTOR signaling, providing a rationale for evaluating these pathway-directed combinations in IBC.

## Materials and methods

### Cell lines and compounds

Four breast cancer cell lines were used in this study: SUM149PT (SUM149) and SUM159PT (SUM159) (obtained from BioIVT, Westbury, NY, USA), and MDA-MB-231 and MCF-7 (obtained from American Type Culture Collection, ATCC, Manassas, VA, USA). Compounds identified in the LWAS [28] (n = 24) and the GRR [29] (n = 16; 16 of 19 were commercially available) were purchased from Selleck Chemicals (Houston, TX, USA) and Sigma-Aldrich (St. Louis, MO, USA). All compounds were supplied as powders, dissolved in 100% DMSO to a stock concentration of 10 mM, and stored at −20°C.

### Cell culture

SUM149 and SUM159 cells were cultured in Ham’s F-12 medium supplemented with 5% FBS, 1 µg/mL hydrocortisone, 5 µg/mL insulin, 1% Penicillin/Streptomycin (10,000 units/mL), and 10 mM HEPES, according to BioIVT guidelines. MCF-7 cells were maintained in EMEM supplemented with 0.01 mg/mL human recombinant insulin, 10% FBS, and 1% Penicillin/Streptomycin, following ATCC protocols. MDA-MB-231 cells were cultured in Leibovitz’s L-15 Medium supplemented with 10% FBS and 1% Penicillin/Streptomycin, as recommended by ATCC. SUM149, SUM159, and MCF-7 cells were incubated at 37°C in a humidified atmosphere with 5% CO_2_. MDA-MB-231 cells were maintained at 37°C with free gas exchange and 0% CO_2_.

### Compound identification: computational approaches used to identify potential drugs for repurposing for IBC

Our prior work utilized two *in silico* approaches to identify drugs predicted to be potential repurposed candidates for IBC. In the LWAS study [28], we used Word2Vec-based text-mining [33] trained on ~3.8 million cancer-related PubMed abstracts to generate vector embeddings for diseases and drugs. Disease–disease and drug–disease similarity maps were visualized using t-distributed stochastic neighbor embedding (t-SNE) [34]. Diseases that co-occur with IBC were identified from the maps [28], with the concept being that drugs for diseases that cluster with IBC in the literature-derived embedding space can be potentially repurposed for IBC. Mapping these IBC-neighbor diseases to curated drug–disease associations from public resources (DrugBank and KEGG) yielded a list (S1 Table) of 24 oncology-based compounds [28] for experimental evaluation in IBC models.

The GRR approach [32] identifies drugs predicted to reverse a disease-associated gene expression signature back to a more “normal/healthy-like” transcriptional state. In our previous GRR study (Ji 2023), we first compiled a list of IBC-associated genes from six published IBC transcriptomic studies [35–40], which, after filtering to remove duplicates and genes lacking consistent directionality data, yielded a collated 387-gene IBC gene expression signature (IBC-GES consisting of 265 upregulated and 122 downregulated genes). Of the 387 IBC-GES genes, 297 of the genes had drug perturbation data in the LINCS L1000 database [41, 42]. We then used this 297 IBC-GES subset to query LINCS for those drug treatments that reversed any of the IBC-GES genes (irrespective of cell type in LINCS) and ranked the drug treatments based on the percentage of genes the drug (at its reported dose in LINCS) was calculated to reverse (gene reversal rate, GRR). Using this approach we identified 32 drugs with GRR values >50% [29]. We then analyzed binary combinations of these drugs and selected combinations predicted to reverse at least 80% of the 297-gene IBC-GES. This analysis identified 19 unique drugs [29] (S2 Table) for experimental evaluation in IBC models.

### High-content imaging cell proliferation assay

Cell proliferation was quantified using an automated Hoechst 33342 DNA staining method, as previously described [43, 44]. Breast cancer cell lines (SUM149, SUM159, MDA-MB-231, and MCF-7) were seeded in 384-well microplates (Corning, 3764) at optimized densities of 800, 600, 1600, and 2500 cells/well, respectively. Cell suspensions were dispensed at 50 µL per well using a MultiFlo Dispenser (Agilent BioTek). Each plate included negative controls (0.1% DMSO) and positive controls (staurosporine at 10 µM and 1 µM). After 24 h, cells were treated with test compounds dispensed by a D300 Digital Dispenser (HP Inc., Palo Alto, CA, USA). Test compounds were dispensed in a 20-point dose-response series using 1:2 serial dilutions, starting at 10 µM. After 72 h of treatment, cells were stained with Hoechst 33342 using a Biomek NX Automated Workstation (Beckman Coulter, Brea, CA, USA). The dye was added at a final concentration of 10 µg/mL, and plates were incubated for 45 min at 37°C. The culture medium and dye were then aspirated, and cells were fixed with 20 µL of 4% formalin for 15 min at room temperature. After removing the fixative, 50 µL PBS was added to each well. Cell nuclei were imaged and quantified using a CellInsight CX7 High-Content Screening (HCS) Platform (Thermo Fisher Scientific). Five fields per well were captured using a 10 × objective. Data were analyzed using GraphPad Prism 9.0 (GraphPad Software, San Diego, CA, USA). All experiments were performed with three technical replicates in each of three independent experiments, and results are presented as mean ± SD.

### Automated cell viability assay

Cell viability was evaluated in four breast cancer cell lines (SUM149, SUM159, MDA-MB-231, and MCF-7) using an automated high-throughput screening approach [45]. The cells were seeded at optimized densities (SUM149: 800 cells/well; SUM159: 600 cells/well; MDA-MB-231: 4000 cells/well; MCF-7: 2500 cells/well) in 384-well flat-bottom polystyrene microplates (Corning, Cat. #3701). Cell suspensions were dispensed using a MultiFlo Dispenser (Agilent BioTek, Santa Clara, CA, USA) in 50 µL of complete growth medium per well. Following 24 h, cells were treated with test compounds in a dose-response format as described above. Each plate contained maximum solvent control wells (0.1% DMSO) and minimum control wells (no cells, culture medium only). Cell viability was quantified 72 h post-treatment using the MTT (3-(4,5-dimethylthiazol-2-yl)-2,5-diphenyltetrazolium bromide) colorimetric assay. MTT solution was delivered to each well at a final concentration of 0.5 mg/mL using a Biomek NX Automated Workstation (Beckman Coulter, Brea, CA, USA). After a 4 h incubation at 37°C to allow formazan crystal formation, the culture medium was aspirated, and the crystals were solubilized in 40 µL DMSO by incubating the plates for 1 h in the dark at room temperature. Absorbance was measured at 550 nm using a SpectraMax Microplate Reader (Molecular Devices, San Jose, CA, USA). Cell viability percentages were calculated by normalizing sample absorbance values to vehicle-control wells. Dose-response curves were generated, and the half-maximal inhibitory concentration (IC_50_) was calculated using GraphPad Prism 9.0 (GraphPad Software, San Diego, CA, USA). All experiments were conducted in three independent experiments, with data presented as mean ± standard deviation (SD).

### Drug combination screening

The synergistic effects of drug combinations were evaluated in 2D SUM149 cells using the Hoechst 33342 DNA staining assay. This assay was performed over five days as described above. The combination effects were assessed in two sequential phases: primary screening followed by secondary validation screening.

### Primary combination screening: single-dose drug combinations

Each pair of compounds was combined in the same well at the respective EC_25_ concentration of each compound to get a singular dose. This EC_25_ concentration that achieves 25% inhibition was calculated using the following equation, in which F is the desired response level, and H is the Hill slope:


$$\begin{array}{c} {𝐄𝐂}_{𝐅}={\left(\frac{𝐅}{100-𝐅}\right)}^{\frac{1}{𝐇}}\times \;𝐄{𝐂}_{50} \end{array}$$ (1)

In total, 78 combinations from the LWAS study and 15 combinations from the GRR study were evaluated. Synergy analysis was performed using the Bliss excess Independence method [46] using the following equation:


$$\begin{array}{c} 𝐁𝐥𝐢𝐬𝐬\;𝐞𝐱𝐜𝐞𝐬𝐬=𝐕𝐢𝐚𝐛𝐢𝐥𝐢𝐭𝐲\;\left(𝐃𝐫𝐮{𝐠}_{𝐀}+𝐃𝐫𝐮{𝐠}_{𝐁}\right)-\left(𝐕𝐢𝐚𝐛𝐢𝐥𝐢𝐭𝐲\;\left(𝐃𝐫𝐮{𝐠}_{𝐀}\right)\text{ x }𝐕𝐢𝐚𝐛𝐢𝐥𝐢𝐭𝐲\;\left(𝐃𝐫𝐮{𝐠}_{𝐁}\right)\right) \end{array}$$ (2)

The Bliss excess score, with negative values indicating synergy, was calculated across quadruplicate technical replicates to assess statistical significance.

### Secondary drug combination screening: matrix-designed drug treatments

An 8 × 8 dose-response matrix [47, 48] was designed using ½ log serial dilutions for the top five combinations from each list, based on the Bliss synergy scores from the primary screening. This was implemented using the Synergy Wizard feature in the D300 Control Software (HP Inc.). The highest concentration in each matrix was selected based on each drug’s IC_50_ value. Staurosporine (10 µM) was used as a positive control and DMSO (0.2%) as a negative control. DMSO tolerance in SUM149 cells was confirmed up to 0.8%. The experiment was conducted with three technical replicates and three independent experiments. Percent inhibition and synergy scores were calculated and analyzed using SynergyFinder3 [49] and SynergyFinder+ [50] web-based tools.

### Quantification of cell viability in SUM149 spheroids

Cell viability was assessed using the CellTiter-Glo Luminescent Cell Viability Assay (Promega, Madison, WI, USA) according to the manufacturer’s instructions. Briefly, SUM149 cells were plated in 384-well microplates (Corning, Cat. No. 3830) at 5000 cells per well and allowed to form a spheroid for 72 h. Spheroids were then treated with selected combinations identified in the 2D combination screen, using staurosporine and DMSO as controls, as described previously. After 72 h, plates were incubated at room temperature for 30 min. An equal volume of CellTiter-Glo reagent was added to each well, and plates were then shaken for 5 minutes to promote cell lysis, followed by 30 min incubation at room temperature. Luminescence was measured using a PHERAstar FSX detection system (BMG LABTECH, Cary, NC, USA). Cell viability was calculated as a percentage relative to vehicle-treated control cells. Each experiment was performed in triplicate and repeated in three independent experiments. Data were analyzed using SynergyFinder+ and SynergyFinder 3 web-based tools.

### Cell death evaluation and spheroid imaging

SYTOX Red Dead Cell Stain (Invitrogen, Cat. No. S34859) was used to visualize spheroid morphology and integrity. Spheroids were formed and treated in 384-well microplates as described above in for the CellTiter-Glo assay. SYTOX Red was added to a final concentration of 40 nM immediately after treatment. Imaging was performed using an IncuCyte S3 Live-Cell Analysis System (Sartorius, Ann Arbor, MI, USA) equipped with a 10 × objective. The IncuCyte system was maintained at 37°C and 5% CO_2_ throughout the experiment. Images were acquired every 6 h for 3 days, capturing both brightfield and red fluorescence (excitation 585 nm, emission 635 nm) channels. Spheroid size and fluorescence intensity were quantified using the IncuCyte S3 software. A minimum of four spheroids were analyzed per condition.

### Wound healing and cell migration

SUM149 cells were seeded at 60,000 cells per well in an ImageLock 96-well plate (Essen Bioscience, Ann Arbor, Michigan) and allowed to attach overnight at 37°C in a humidified atmosphere with 5% CO_2_. A uniform vertical scratch wound was created across all wells using the automated WoundMaker™ tool (Essen BioScience). Cells were gently washed with medium to remove detached cells, and fresh culture medium was added before the combination treatment using a D300 Digital Dispenser (HP). Cell migration into the wound area was monitored in real-time using the IncuCyte® S3 Live-Cell Analysis System (Sartorius) as previously described [44]. Images were automatically monitored in real time at 0, 6-, 12-, 18-, and 24-h post-wounding using a 10 × objective lens, enabling quantitative assessment of wound closure kinetics.

### Western blotting

SUM149 cell lysates (after 24 h drug treatment) were collected using M-PER buffer supplemented with protease/phosphatase inhibitors (Thermo Fisher Scientific, Cat. No. A32959). Lysates were centrifuged (14,000 × g, 15 min, 4°C) and protein concentrations were determined by BCA assay (Pierce, Cat. No. 23225). Equal protein amounts (15−30 μg/lane) were loaded, and samples were electrophoresed on 4−12% Bis-Tris gels (Invitrogen, Cat. No. NP0336BOX). For mTOR and p-mTOR, samples were run on 8% Tris-Glycine gels (Invitrogen, Cat. No. XP00085BOX). Proteins were transferred to 0.45 μm PVDF membranes (Invitrogen, Cat. No. LC2005) using an XCell II™ Blot Module (Overnight, 4°C). Membranes were blocked with 5% BSA in TBS (pH 7.4) for 1 h at room temperature. Primary antibodies were diluted in 5% BSA in TBS-T (0.1% Tween-20) and incubated with membranes overnight at 4°C. All primary antibodies were used at 1:1000 dilution except anti-β-Actin and anti-p-Akt ((Ser473) which were used at 1:2000. Antibodies included: anti-β-Actin (45 kDa, LI-COR, No. 926−42212), anti-Akt (60 kDa, CST, No. 9272), anti-p-Akt(Ser473) (60 kDa, CST, No. 4060), anti-IGF-1R (95 kDa, CST, No. 3027), anti-p-IGF-1R(Tyr1135/1136) (95 kDa, CST, No. 3024), anti-EGFR (175 kDa, CST, No. 4267), anti-p-EGFR(Tyr1068) (175 kDa, CST, No. 3777), anti-mTOR (289 kDa, CST, No. 2983), and anti-p-mTOR(Ser2448) (289 kDa, CST, No. 2971). After TBS-T washes (3 × 10 min), membranes were incubated with either IRDye secondary antibodies (800CW/680RD, LI-COR, 1:20,000 in 5% BSA/TBS-T [0.2% Tween-20]/0.02% SDS) or HRP-linked antibody (1:10,000, CST, 7074) for 1 h at room temperature. Following TBS-T washes (3 × 5 min), membranes were imaged using Odyssey CLx (LI-COR) or iBright Systems (Invitrogen). For IGF-1R and pIGF-1R (and β-Actin control for these samples), HRP secondary antibody was used for detection, and blots were imaged by iBright. Analysis was performed using Image Studio (v5.2, LI-COR) or iBright software. Band intensities were normalized to total protein stain (for LI-COR) and β-Actin (for iBright). Experiments were performed in triplicate (n = 3), with significance determined as *p* < 0.05.

### RNA extraction and RNA-Seq analysis

Total RNA was extracted from SUM149 cultured cells after 24 h of treatment with either drug combinations or DMSO vehicle control, using the RNeasy Mini Kit (Qiagen, 74104), following the manufacturer’s instructions. RNA concentration and purity were assessed using a NanoDrop 2000 spectrophotometer (Thermo Fisher Scientific). The absorbance ratios A260/A280 and A260/A230 were used to evaluate RNA quality and purity. Samples with A260/A280 ratios between 1.8 and 2.1 and A260/A230 ratios above 1.8 were considered of suitable quality for RNA-Seq analysis. All samples were prepared as independent replicates (n = 3). RNA-Seq analysis was performed by MedGenome (MedGenome Inc., Foster City, CA, USA) following their standard pipeline, beginning with the preparation of RNA-Seq libraries using the KAPA mRNA HyperPrep kit, which were then sequenced on an Illumina NovaSeq 6000 / X Plus platform to produce 100-bp paired-end reads. Raw reads underwent quality control with FastQC v0.11.9; adapters and low‑quality bases were trimmed using fastq-mcf v1.05 and cutadapt v4.7. Potential contaminants (rRNA, tRNA, adapters, mitochondrial sequences) were filtered with Bowtie2 v2.5.3, and the remaining reads were aligned to the human Ensembl GRCh38 (release 87) reference using STAR v2.7.11b in two‑pass mode. Gene‑level counts were assigned with HTSeq v2.0.5. and count matrices were normalized with DESeq2 v1.10.1 in R. Differential expression was carried out in DESeq2 using contrasts defined as Control_vs_Case (direction reported for the case group relative to control) with significance defined as adjusted *p* value < 0.05 and |Log_2_fold‑change| ≥ 2.

Raw fastq files, processed data files and metadata spreadsheet were uploaded to the GEO repository and after validation and approval on May 18^th^ 2026, received an assigned GEO accession number: GSE331356.

### Data processing and statistical analysis

Statistical analyses and data visualizations were performed using GraphPad Prism 9 (GraphPad Software, Inc., San Diego, CA, USA), with statistical significance set at *p* < 0.05 for all analyses. Drug potency (IC_50_) is defined as the half maximal inhibitory concentration and efficacy (E_max_) is defined as the maximal observed inhibition within the tested concentration range. IC_50_ values were determined using nonlinear logistic regression analysis with a three-parameter log(inhibitor) vs. dose-response function, constrained to a zero-bottom asymptote. SynergyFinder3 and SynergyFinder + , web-based tools, were used to calculate synergy scores based on four widely used reference models: Bliss, Loewe, Highest Single Agent (HSA), and Zero interaction potency (ZIP). *p*-values for synergy scores were determined by SynergyFinder + . All data are presented as mean ± SD, unless otherwise noted.

## Results

### Evaluation of LWAS- and GRR-predicted drug candidates for inhibitory effects in breast cancer cell lines

To assess the efficacy of the drug and compound candidates predicted from our previous LWAS [28] and GRR [29] studies, we conducted high-throughput screening (HTS) using dose-response treatments in an automated 384-well format. Two orthogonal cell-based assays were utilized to determine the efficacy and potency of each candidate: the Hoechst nuclear count assay, a fluorescent DNA staining method for assessing cell number using high-content imaging [43], and the MTT assay to assess effects on cell viability. Screening was conducted across a panel of IBC and non-IBC cell lines: SUM149, SUM159, MDA-MB-231, and MCF-7.

### Assessment of LWAS-predicted candidates for effects on cell proliferation

**Fig 1:**
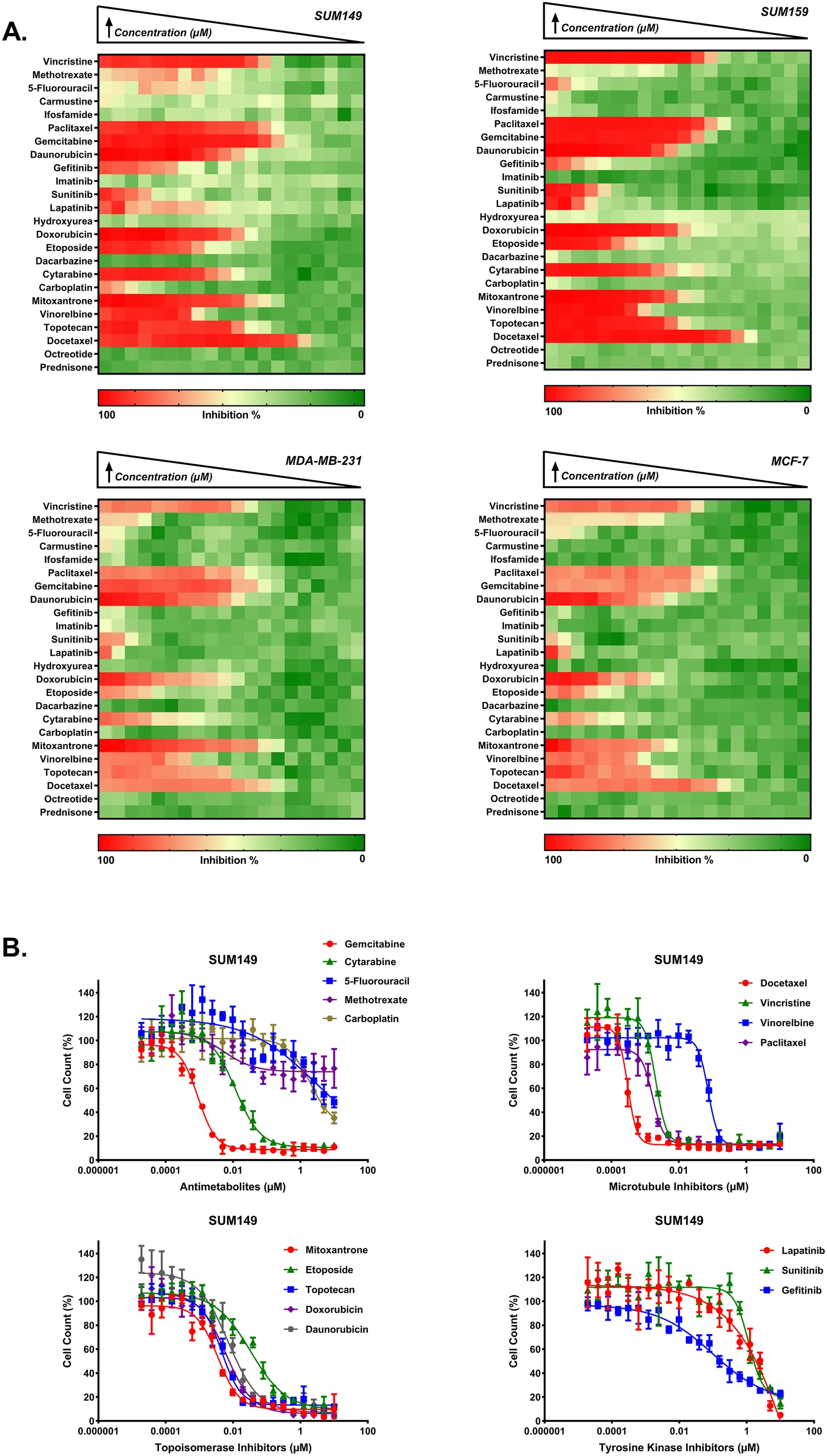
Drug sensitivity profiles for LWAS-predicted drugs and compounds in IBC and non-IBC breast cancer cell line panel (Hoechst assay). (A) Heatmaps representing the percent inhibition response for a 2-fold concentration dose-response starting at 10 µM for SUM149, SUM159, MDA-MB-231, and MCF-7. Drug names are listed on the y-axis. The color scale indicates the level of inhibition, with red representing high inhibition (close to 100%) and green representing low inhibition (close to 0%). (B) Representative dose-response curves (DRC) for the LWAS drugs with activity in the SUM149 cell line, showing the percent cell count relative to control against drug concentration (µM). Plots are grouped by drug mechanism: antimetabolites, microtubule inhibitors, topoisomerase inhibitors, and TKIs. Data points and error bars represent the mean ± SD. DRCs for the LWAS drugs in SUM159, MDA-MB-231, and MCF-7 are in S1 Fig.

Our previous LWAS text mining study [28], used a natural language processing-based model trained on a large corpus of cancer-related abstracts to identify diseases that co-occur with IBC, with the concept that drugs used for diseases clustering near IBC could be candidate repurposing agents for IBC. This LWAS analysis [28] identified 24 oncology-based compounds with different mechanistic classes: tyrosine kinase inhibitors (TKIs), antimetabolites, topoisomerase inhibitors/anthracycline antibiotics, microtubule inhibitors, and alkylating agents (S1 Table). In this current study, we first evaluated the activity of these 24 compounds [28] using our lab-based Hoechst assay to quantify effects on cell number and proliferation. We found significant antiproliferative activity (IC_50_ < 10 μM) for 17 compounds in SUM149, 16 in SUM159, and 15 in both MDA-MB-231 and MCF-7 cell lines (shown in heatmap format in Fig 1A). Comparative analysis across cell lines revealed a graded pattern of drug sensitivity, with SUM149 demonstrating the highest responsiveness, particularly to microtubule inhibitors and antimetabolites. Analysis of median IC_50_ values across compound classes showed decreasing sensitivity in the following order: SUM149 > SUM159 > MDA-MB-231 > MCF-7, which may correlate with these cells’ relative doubling times. Among the compound classes, microtubule inhibitors demonstrated the highest potency, with docetaxel, paclitaxel, and vincristine showing low nanomolar activity (IC_50_: 0.2–13 nM) across all cell lines. Topoisomerase inhibitors represented the second-highest set of potent compounds, with doxorubicin and daunorubicin achieving IC_50_ values below 0.2 μM. The TKIs lapatinib and sunitinib showed moderate micromolar potency (IC_50_: 0.57–7.3 μM and 1.4–5.3 μM, respectively), while gefitinib showed selective activity against SUM149 and SUM159 (0.4–10 μM, respectively). Within the antimetabolite class, gemcitabine yielded high potency (IC_50_: 0.001–0.29 μM) and cytarabine yielded moderate potency (IC_50_: 0.023–0.65 μM). The alkylating agent carboplatin showed the lowest potency and was active only in SUM149 cells (IC_50_ = 6.04 μM). The remaining compounds from the LWAS screen (n = 7) showed minimal antiproliferative effects against SUM149 (IC_50_ > 10 μM). Representative dose-response curves (DRC) for SUM149 are shown in Fig 1B, while DRCs for SUM159, MDA-MB-231, and MCF-7 cell lines are in S1 Fig. Complete IC_50_ values (nM) are summarized in Table 1.

**Table 1:** Efficacy of LWAS-predicted drugs and compounds in four breast cancer cell lines: SUM149, SUM159, MDA-MB-231, and MCF-7 (Hoechst assay).

Drug	SUM149	SUM159	MDA-MB-231	MCF-7
	(IC_50_ nM)	(IC_50_ nM)	(IC_50_ nM)	(IC_50_ nM)
Docetaxel	0.4 ± 0.06	0.5 ± 0.02	1.2 ± 0.3	0.85 ± 0.1
Paclitaxel	2.0 ± 0.3	2.0 ± 0.1	2.5 ± 0.7	2.0 ± 0.4
Vincristine	4.0 ± 0.1	3.0 ± 0.2	9.0 ± 5.0	3.0 ± 0.8
Vinorelbine	90 ± 20	70 ± 7	250 ± 80	75 ± 8
Doxorubicin	10 ± 3	10 ± 1	30 ± 9	80 ± 11
Daunorubicin	10 ± 3	20 ± 1	20 ± 5	40 ± 3
Mitoxantrone	5 ± 0.3	10 ± 1	3 ± 0.7	20 ± 9
Etoposide	70 ± 16	100 ± 70	1100 ± 500	1300 ± 400
Topotecan	8 ± 1	12 ± 0.4	30 ± 10	80 ± 60
Gemcitabine	1 ± 0.1	4 ± 0.1	5 ± 2	4 ± 1
Cytarabine	25 ± 5	23 ± 3	200 ± 100	500 ± 200
Methotrexate	10 ± 8	10 ± 7	900 ± 120	20 ± 4
Lapatinib	600 ± 500	2100 ± 800	6500 ± 1000	4000 ± 400
Sunitinib	1400 ± 300	700 ± 100	1900 ± 300	3900 ± 800
Gefitinib	400 ± 160	900 ± 30	--	--
Carboplatin	4400 ± 500	--	--	--
5-Fluorouracil	4800 ± 600	2400 ± 1700	12400 ± 3900	3900 ± 3300

IC_50_ values (nM) determined for each drug by the Hoechst assay across the cell lines, with values reported as the mean ± SD from 3 independent experiments.

### Assessment of GRR-predicted candidates for effects on cell proliferation

**Fig 2:**
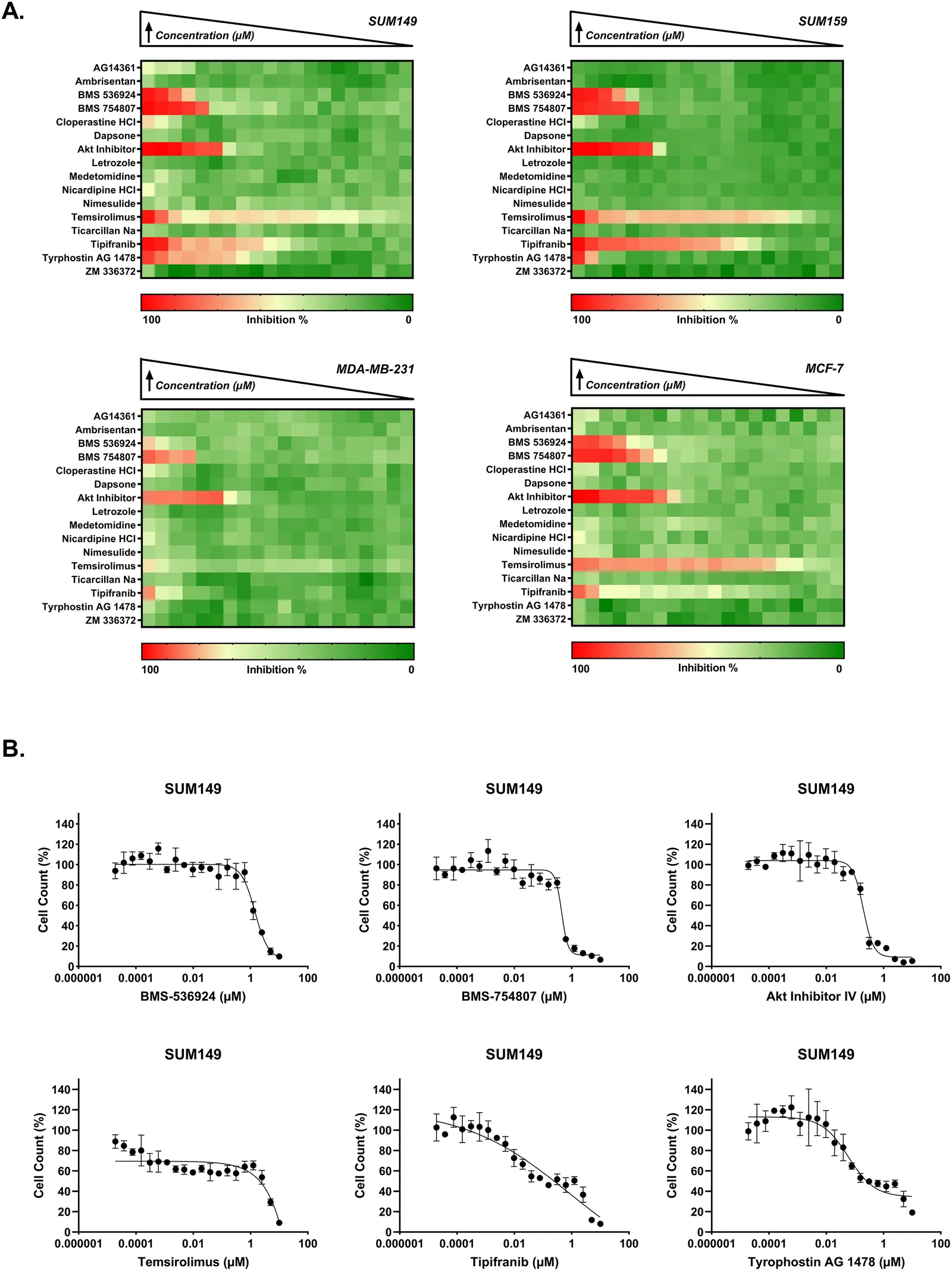
Drug sensitivity profiles for GRR-predicted drugs and compounds in IBC and non-IBC breast cancer cell line panel (Hoechst assay). (A) Heatmaps representing the percent inhibition response for a 2-fold concentration dose-response starting at 10 µM for SUM149, SUM159, MDA-MB-231, and MCF-7. Drug names are listed on the y-axis. The color scale indicates the level of inhibition, with red representing high inhibition (close to 100%) and green representing low inhibition (close to 0%). (B) Representative dose-response curves for active inhibitors BMS-536924, BMS-754807, AKTIV, temsirolimus, tipifarnib, and tyrphostin AG 1478 in the SUM149 cell line, showing the percent cell count relative to control against drug concentration (µM). Data points and error bars represent the mean ± SD. DRCs for SUM159, MDA-MB-231, and MCF-7 are in S2 Fig.

In our previous GRR analysis, we used a compiled IBC-gene expression signature (IBC-GES) to query the LINCS database for drugs that significantly reversed expression of the majority of IBC-GES genes and identified 19 drugs predicted to have effects on IBC [29]. Here, we evaluated the anti-proliferative activity of these GRR-predicted candidates [29] using the Hoechst assay in the same four cell lines described above; 16 of the 19 predicted compounds were commercially available. The GRR-predicted candidates showed differential anti-proliferative activity in each cell line, with significant effects (IC_50_ < 10 μM) observed for 6 compounds in SUM149, SUM159 and MDA-MB-231, and 5 in MCF-7 cells (represented as heatmaps in Fig 2A). Although the GRR list comprises both oncology and non-oncology compounds from diverse drug classes (S2 Table), the active compounds were primarily TKIs, with Akt Inhibitor-IV (AKTIV) being an exception. In SUM149, tipifarnib displayed the highest potency (absolute IC_50_ = 0.075 μM), followed by tyrphostin AG-1478 and AKTIV, which showed comparable potency (absolute IC_50_ ≈ 0.2 μM). Tipifarnib showed a biphasic DRC, and upon calculating the relative IC_50_ in SUM149, it shifted to 7 ± 0.2 nM (10-fold increase in potency). Tyrphostin AG-1478 showed a low efficacy curve (indicating antiproliferative activity), its relative IC_50_ in SUM149 shifted to 0.05 ± 0.01 μM (4-fold increase in potency). Temsirolimus displayed varying potency across the 4 cell lines with an absolute IC_50_ of 4.3 μM in SUM149, compared to much higher potency in SUM159, MDA-MB-231, and MCF-7 cell lines (IC_50_ range: 0.2–0.6 nM). Temsirolimus also showed a biphasic DRC in SUM149 cells, with a relative IC_50_ value of 0.1 nM, which is comparable to other cell lines but with lower efficacy (approximately 60% maximum inhibition). MDA-MB-231 showed unique sensitivity to nicardipine (projected IC_50_ = 16.7 μM), while showing reduced sensitivity to the other compounds compared to other cell lines. Tyrphostin AG-1478 showed no inhibitory activity in MCF-7, unlike other cell lines. Representative DRCs for SUM149 are shown in Fig 2B, while DRCs for SUM159, MDA-MB-231, and MCF-7 cell lines are in S2 Fig, showing a range of responses. The IC_50_ values (µM) for the GRR-predicted compounds are summarized in Table 2.

**Table 2:** Efficacy of the GRR-predicted drugs and compounds in four breast cancer cell lines: SUM149, SUM159, MDA-MB-231, and MCF-7 (Hoechst assay).

Drug	SUM149	SUM159	MDA-MB-231	MCF-7
	(IC_50_ µM)	(IC_50_ µM)	(IC_50_ µM)	(IC_50_ µM)
BMS-536924	1.9 ± 0.1	0.8 ± 0.3	8.3 ± 3.4	1.4 ± 0.1
BMS-754807	0.5 ± 0.1	0.5 ± 0.04	2.0 ± 0.34	0.4 ± 0.06
AKTIV	0.2 ± 0.01	0.2 ± 0.02	0.1 ± 0.025	0.2 ± 0.03
Temsirolimus	4.3 ± 0.3	0.0003 ± 0.0004	0.0006 ± 0.0004	0.0002 ± 0.0001
Tipifarnib	0.08 ± 0.03	0.0008 ± 0.0002	6.0 ± 1.3	5.8 ± 3
Tyrphostin AG-1478	0.2 ± 0.15	3.5 ± 1.4	9.2 ± 4.2	--
Nicardipine	--	--	16.7 ± 6.1	--

IC_50_ values (µM) determined for each drug by the Hoechst assay across the cell lines, with values reported as the mean ± SD from 3 independent experiments.

### Assessment of LWAS- and GRR-predicted candidates for efficacy in the MTT cell viability assay

To provide an orthogonal readout of cell viability, we also evaluated the LWAS- [28]and GRR-predicted [29] compounds using the MTT assay. Testing the compounds in the MTT assay yielded IC_50_ values and DRCs comparable to those of the Hoechst assay for both the LWAS (S3 Fig) and the GRR (S4 Fig) compounds. All the IC_50_ values for the LWAS drugs and GRR compounds in the MTT assay across the 4 cell lines are summarized in S3 Table. For the MTT assay, using an activity threshold of IC₅₀ < 10 µM, the LWAS list screening yielded 17 active compounds in SUM149, 16 in SUM159, 15 in both MDA‑MB‑231 and MCF-7, whereas the GRR panel yielded 6 anti-proliferative compounds in SUM149, SUM159 and MDA-MB-231, and 5 in MCF-7. The same compounds were active in both the Hoechst and MTT assays and maintained the same overall rank-order of potency. For LWAS compounds tested in the MTT assay, microtubule inhibitors again were the most potent class with nanomolar potency in both assays across all cell lines, and the median docetaxel IC₅₀ shifts by <1.5‑fold (e.g., 0.0004 µM in SUM149 by Hoechst versus 0.0003 µM by MTT). Likewise, topoisomerase inhibitors retain mid-nanomolar potency, while antimetabolites gemcitabine and cytarabine showed only ≤ 2-fold differences between assays. For the GRR list, the potencies for each compound in both assay formats were comparable, in the range of 1‑ to 3‑fold difference. In SUM149, temsirolimus showed an IC_50_ value of 4.3 µM in the Hoechst assay and 7.3 µM in MTT assay. For AKTIV, BMS‑536924 and BMS‑754807, the fold shifts are generally below 3, except for a 7-fold change for BMS‑754807 in SUM149 (IC_50_: 3.6 µM in MTT assay 0.5 µM in Hoechst assay). For both assays, tyrphostin AG‑1478 shows comparable nanomolar activity in SUM149, and comparable micromolar activity SUM159 cell line. Exceptions included tipifarnib in SUM159, where the IC_50_ was 0.8 nM by Hoechst compared to 2.1 µM by MTT, and gefitinib, which was ~ 10-fold less potent in SUM159 by MTT (IC_50_: 11 µM) than by Hoechst (IC_50_: 0.9 µM). These differences might reflect the mechanism behind each assay where cytostatic effect manifests faster in the nuclear‑count readout (Hoechst) than in the metabolic assay (MTT). Hoechst-based imaging primarily quantifies cell number as a direct readout of nuclear count, whereas MTT is a tetrazolium-reduction assay that reflects cellular redox/metabolic activity. For tipifarnib, the lower IC_50_ by Hoechst (0.8 nM) compared with MTT (2.1 µM) is consistent with a cytostatic/anti-proliferative response at low concentrations (e.g., G1 arrest), which might reduce cell nuclei count without necessarily causing a proportional decrease in metabolic activity in the remaining cell [51, 52]. The EGFR inhibition by gefitinib is also associated with cell-cycle arrest and growth suppression, which can yield a lower IC_50_ in a cell-number endpoint assay (Hoechst; 0.9 µM) than in a metabolism-based endpoint assay (MTT; 11 µM) [53]. Most IC_50_ values differed by less than 2-fold, indicating strong concordance (85%) between the nuclear-count and metabolic readouts in all cell lines with SUM149 being the most responsive cell line in both formats.

### Assessing drug combinations in SUM149 for synergistic effects

**Fig 3:**
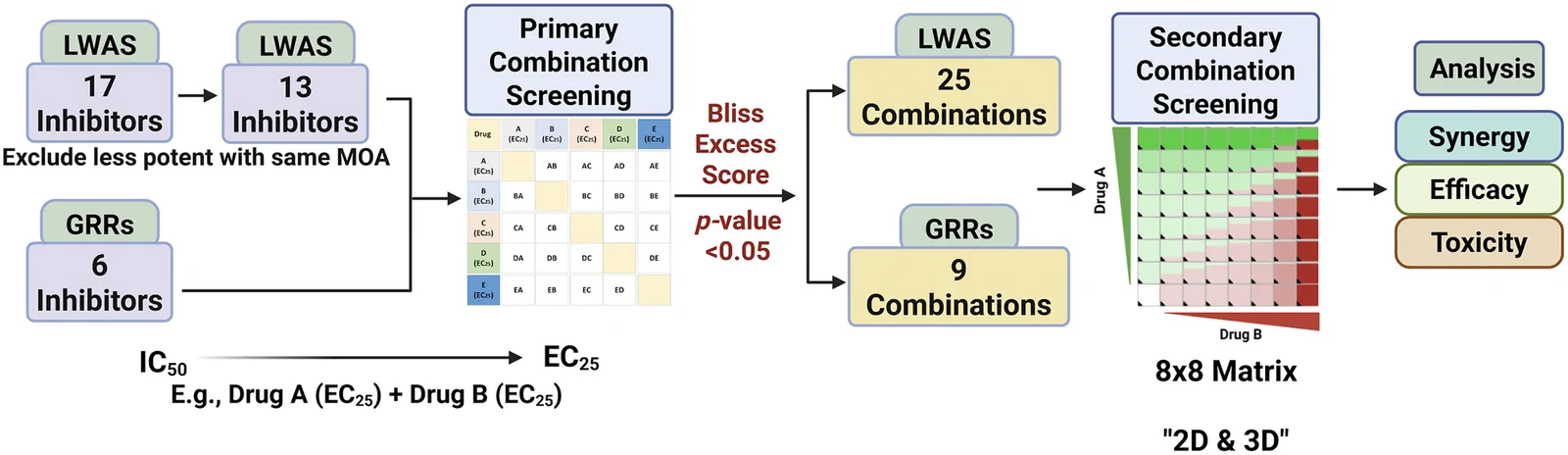
Workflow for identification and evaluation of synergistic drug combinations. The combination screening process began with the 17 LWAS-predicted inhibitors shown to have efficacy in the Hoechst assay (Table 1), which was refined to 13 LWAS inhibitors after excluding less potent compounds with similar mechanisms of action (MOA) plus the 6 GRR-predicted inhibitors shown to have efficacy in the Hoechst assay (Table 2). Primary drug combination screening was done using drug pairs at their respective EC_25_ values. The identified combinations were selected based on Bliss excess scores and statistical significance (p value < 0.05), which identified 25 LWAS and 9 GRR synergistic combinations to be confirmed in the secondary screening phase, utilizing an 8 × 8 matrix format. The final analysis included synergy assessment and efficacy evaluation in 2D and 3D cell models.

The potential synergistic effects of drug combinations were assessed in the SUM149 cells which were used as the representative IBC model. The SUM149 cell line was derived from an IBC patient tumor [54], and is a well-established model for IBC [44,45,55–57]. Based on the Hoechst and MTT efficacy profiling results of single compounds, active LWAS and GRR-predicted compounds were selected for subsequent combination studies using a two-tiered approach that included primary and secondary screening. In the primary screening, every two compounds were combined and tested at their respective EC_25_ values. The EC_25_ value was chosen to achieve a modest effect on cell viability, thereby minimizing toxicity and avoiding one drug overwhelming the response. In this case, each drug can affect cellular activity without killing most cells, creating an opportunity to observe an extra effect when the drugs are combined [58]. The 17 active LWAS inhibitors were reduced to 13 by excluding less potent compounds with similar mechanisms of action (MOA), yielding a total of 78 unique two-drug combinations. Likewise, combining the 6 GRR inhibitors gave 15 unique two-drug combinations. The Bliss excess scores [46] were calculated, and a one-sample t-test was used to determine the statistical significance. Combinations that showed statistical significance were subsequently validated in the secondary combination screening phase using 8 × 8 dose-response matrices in 2D cell models and then confirmed in 3D spheroid models to enable a more comprehensive assessment. Synergy evaluation was performed using SynergyFinder3 [49] and SynergyFinder+ [50] online tools. The complete experimental workflow is summarized in Fig 3.

### Identification of key combinations using single-dose primary screening

**Fig 4:**
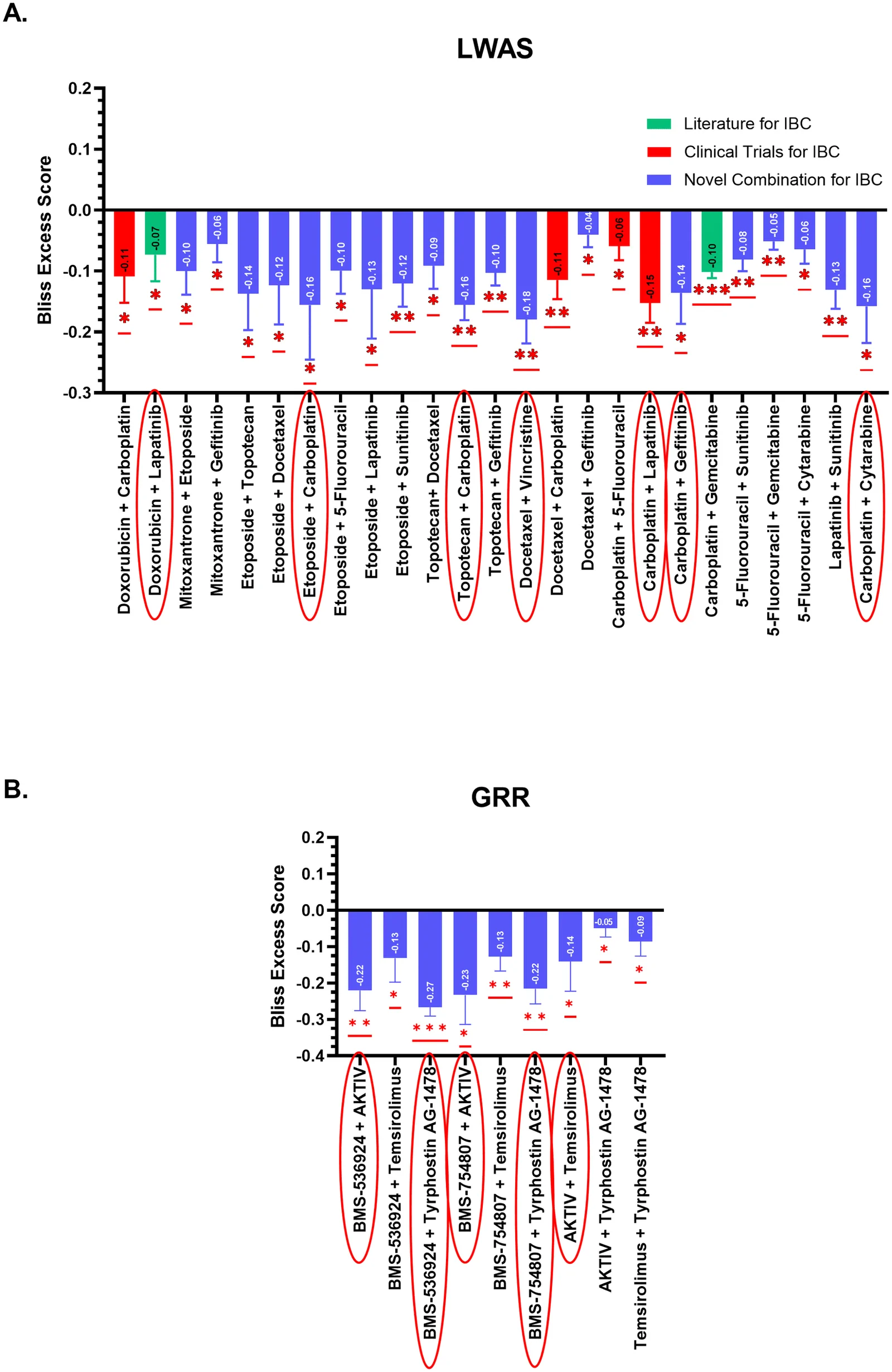
Drug combinations identified from primary combination screening based on Bliss excess scores in SUM149. Primary drug combination screening was done using drug pairs at their respective EC_25_ values. Each combination is evaluated based on percentage viability, with negative scores indicating synergism. (A) Twenty-five statistically significant synergistic combinations were identified from the LWAS study and categorized as reported in the literature for IBC (green bars), tested in clinical trials for IBC (red bars), and novel combinations for IBC (blue bars). (B) Nine statistically significant synergistic combinations were identified from the GRR list, all of which were novel for IBC. Red asterisks indicate statistically significant deviations centered at zero based on a t-test (*p < 0.05; **p < 0.01; ***p < 0.001). Red oval shapes highlight the selected combinations for further screening in the 8 × 8 matrix.

Our initial primary screening analysis, while using only single EC_25_-dose combinations, revealed potential drug synergy based on Bliss excess score calculations, in which negative values indicate a synergistic effect when starting with cell viability data [46]. Quantitative analysis of the primary combination screen identified 25 synergistic combinations from the LWAS list (Fig 4A, 32.1% success rate, *p* < 0.05) and 9 synergistic combinations from the GRR list (Fig 4B, 60% success rate, *p* < 0.05). These synergistic combinations demonstrated strong negative excess Bliss scores ranging from −0.16 to −0.27. The success rate for both single and combination treatments was defined and calculated as the fraction of selected compounds that met a prespecified activity threshold in the experimental assay. It was computed as SR = N _successful_ / N total _tested_, and is reported as a percentage.

For the LWAS primary combination results, 4 of the 25 combinations (red bar color in Fig 4) have been previously documented in IBC-related clinical trials registered on ClinicalTrials.gov, including where carboplatin was combined with either doxorubicin (NCT05415215), docetaxel (NCT00251329 and NCT00118053), lapatinib (NCT01426880), or 5-fluorouracil (NCT01036087). These trials either included IBC patients or specifically focused on IBC populations. Further, the combination of carboplatin with gemcitabine is listed as a treatment option for IBC [59]. For the GRR primary combination screen, combinations targeting PI3K/AKT/mTOR downstream pathways, either directly or indirectly, showed high synergy scores, including BMS-536924 (a dual IGF-1R/IR tyrosine kinase inhibitor) with tyrphostin AG 1478 (a selective EGFR tyrosine kinase inhibitor); and BMS-536924 with AKTIV (an inhibitor of Akt phosphorylation). To our knowledge, the remaining LWAS combinations (bars highlighted in blue) and all the GRR pairs represent potential novel therapeutic strategies for IBC.

Drug combinations with the highest negative excess Bliss scores (highlighted by red oval shapes in Fig 4, along with two previously reported were selected for secondary combination screening and evaluated using 8 × 8 dose-response matrices.

### Determination of synergy score based on dose-response combination matrix

For our secondary screening, we conducted an 8 × 8 dose-response matrix analysis (Fig 3) to validate promising drug combinations identified from the primary single-dose combination screening. This approach again focused on evaluating drug synergy for SUM149 cell proliferation. For each drug pair (7 for LWAS and 5 for GRR), we designed dose matrices with IC_50_ concentrations positioned centrally, with concentration ranges above and below IC_50_ at ½-log intervals (S4 Table). The experimental protocol involved treating SUM149 cells with drug combinations for 72 h, followed by Hoechst dye-based cell counting. Based on the results from our primary combination screening, we selected the top five combinations from both LWAS and GRR lists. Additionally, we included two other combinations from the LWAS list: one documented in the literature [60] and one found in clinical trials [61] (see Fig 4). We conducted a comprehensive synergy and efficacy analysis to identify the optimal combinations. Synergy scores were calculated using four widely used reference models: Bliss, Loewe, HSA, and ZIP [62]. Bliss/ZIP are generally better suited for combinations of drugs with different targets or modes of action and do not require dose equivalence, while HSA provides a conservative benchmark versus the best single agent at each dose. Loewe assumes dose additivity and performs best when single-agent curves are well defined and drugs have comparable effects [62–64]. According to the framework proposed by Malyutina *et al* [65], our combinations were classified as synergistic (scores > 5) or antagonistic (scores < −5) when meeting these thresholds across all models as a strict criterion. For the efficacy metric evaluation, we used the Combination Sensitivity Score (CSS) developed by Malyutina *et al* [65]. CSS quantifies combination efficacy against cell proliferation by calculating the mean area under the dose-response curve when titrating one drug against the IC_50_ concentration of the second drug (CSS_1_IC50_2_ and CSS_2_IC50_1_). SynergyFinder+ was used to compute the average CSS values for all combinations. In the LWAS combination analysis, we found that none met the strict criterion for overall synergy or antagonism (all the overall synergy (δ) scores fell between −5 and 5 across all reference models, indicating additive effects) (S5 Fig). A closer examination of the contour plots was then undertaken, focusing on the Most Synergistic Area (MSA) [49], which represents a 3 × 3 dose window in the combination matrix that yields the highest positive deviation from the chosen reference model [49]. This analysis of MSA δ scores gave (δ > 5) for five (Loewe model) and seven (HSA model) of the combinations, respectively (S5 Fig, Table 3). This approach can highlight the dose ranges where the interaction is most pronounced and help in selecting concentration pairs for follow-up validation. For example, we observed that the lapatinib + doxorubicin combination gave scores of 26.2 for Loewe and 7.5 for HSA, whereas lapatinib + carboplatin showed scores of 18.6 in Loewe and 10.1 in HSA. However, for the Bliss and ZIP models, MSA δ scores for all these were < 5, reducing confidence that these combinations might be synergistic.

**Table 3:** Synergy scores for the secondary drug combinations (8 × 8 Matrix) in 2D cell models across four reference models.

Study	Drug A	Drug B	Loewe δ score			HSA δ score			Bliss δ score			ZIP δ score			CSS %
			Overall	*p*	MSA	Overall	*p*	MSA	Overall	*p*	MSA	Overall	*p*	MSA	
**LWAS**	LAP	DOX	0.9	2.07e-01	26.2	2.4	9.23e-04	7.5	−3.4	2.73e-03	−0.5	−2.8	9.77e-04	1.6	71.4
	LAP	CARB	1.4	8.32e-01	18.6	1.6	4.47e-02	10.1	−4.1	5.54e-03	1.2	−2.7	3.25e-03	3.1	70.1
	ETOP	CARB	2.2	8.42e-01	6.5	1.7	1.59e-01	6.3	−1.9	6.43e-01	2.7	−1.7	2.06e-01	3.1	56.6
	TPT	CARB	1.4	9.25e-01	3.6	1.3	1.14e-02	6.2	−3.4	5.06e-03	1.1	−2.9	1.10e-03	1.2	59.6
	VCR	DOC	−1.4	1.41e-03	--	0.7	2.25e-01	5.2	−2.9	2.25e-02	2.2	−2.5	1.06e-05	3.5	68.8
	GEF	CARB	−1.4	7.86e-01	7.5	2.3	4.49e-03	8	−1.9	2.14e-01	1.8	−1.3	3.10e-02	2.7	55.6
	ARA-C	CARB	−4.4	1.86e-03	5.9	3.2	2.70e-04	9.2	−2.3	1.36e-01	2.5	−2	3.51e-01	0.1	65.0
**GRR**	AKT IV	TEM	6.1	1.91e-06	12.1	7.9	6.86e-08	16.4	1.3	2.97e-01	4.8	1.4	3.23e-01	5.3	58.6
	BMS-754807	TYR	3.5	4.09e-51	21.6	9	2.50e-52	22.2	4.4	5.36e-10	14.7	4.4	3.19e-13	15.2	71.5
	BMS-754807	AKT IV	2.0	2.99e-01	4.9	2.8	2.06e-02	7.6	−1.3	3.72e-01	1.9	−0.9	4.96e-01	2.3	66.2
	**BMS-536924**	**TYR**	**8.6**	**8.21e-16**	**26.1**	**11.8**	**3.05e-21**	**29.9**	**7.6**	**1.50e-04**	**20.8**	**7.9**	**8.16e-05**	**21.5**	**74.4**
	BMS-536924	AKT IV	5.4	8.07e-06	10.3	5.7	4.19e-10	16.3	1.9	4.23e-02	6.7	2.3	9.44e-05	7.2	79.3

Synergy scores are from SynergyFinder 3. Abbreviations: LAP, lapatinib; CARB, carboplatin; ETOP, etoposide; TPT, topotecan; VCR, vincristine; DOC, docetaxel; GEF, gefitinib; ARA-C, cytarabine; TEM, temsirolimus; TYR, tyrphostin (based on NCBI abbreviation lists).

**Fig 5:**
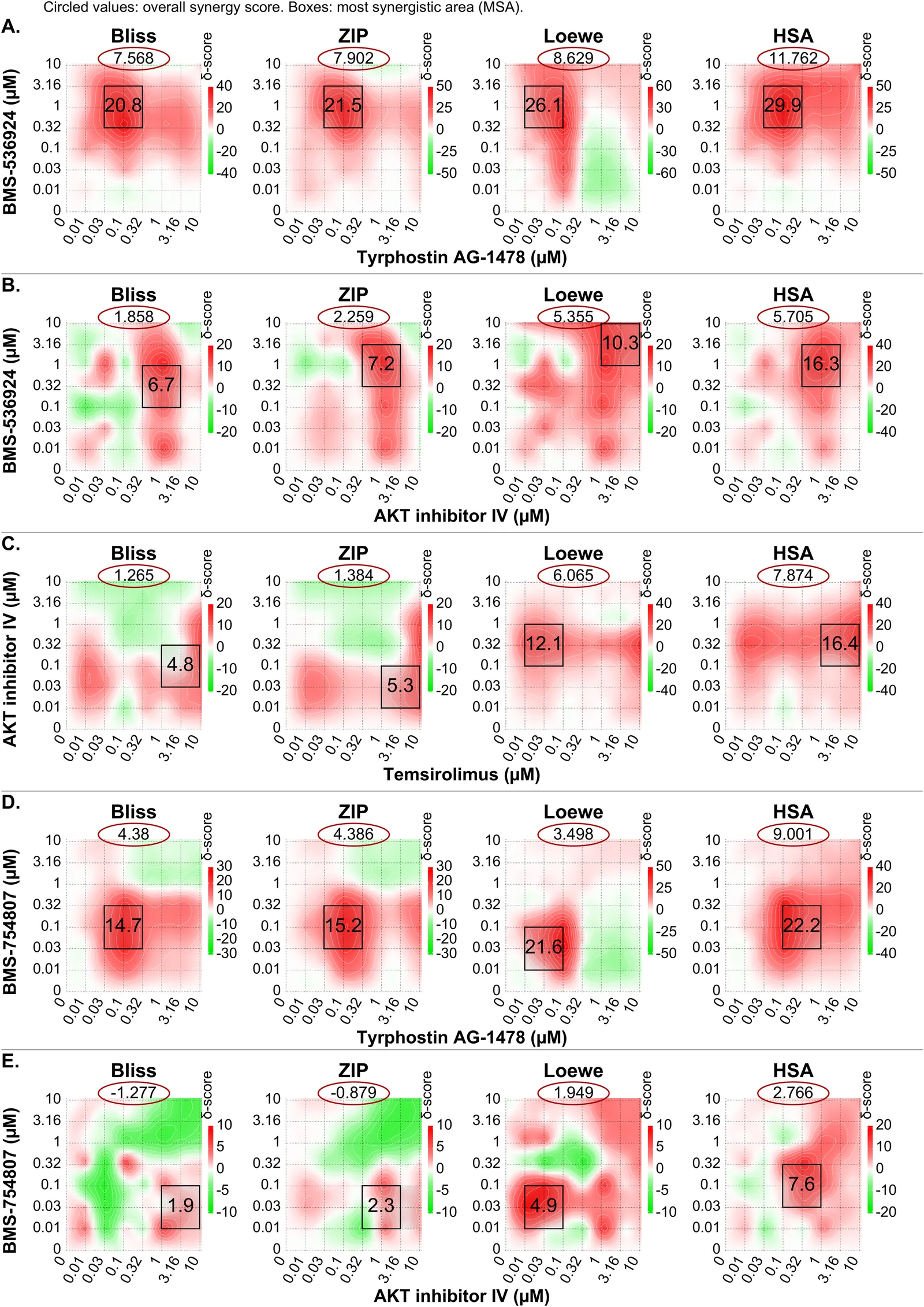
Contour plots of selected GRR-derived drug combinations. Synergy scores across Bliss, ZIP, Loewe and HSA reference models were obtained from SynergyFinder3. (A) BMS-536924 + tyrphostin AG 1478, (B) BMS-536924 + AKTIV, (C) Temsirolimus + AKTIV, (D) BMS-754807 + tyrphostin AG 1478, and (E) BMS-754807 + AKTIV. In each plot, x and y axes represent drug concentrations. Each 8×8 dose-response matrix consists of ½ log serial dilutions starting at 10 µM. Red areas indicate synergy, while green areas indicate antagonism. Black boxes highlight the MSA, with scores shown. Red circles in the top right corner of each plot indicate the overall δ score. Color scales on the right of each plot show the range of synergy scores. The data represent three independent experiments (n = 3).

In the GRR combination analysis, the BMS-536924 and tyrphostin AG-1478 combination (in bold in Table 3) exceeded the 5-score threshold at both the overall (ZIP: 7.9, HSA: 11.8, Bliss: 7.6, Loewe: 8.6) and MSA (ZIP: 21.5, HSA: 29.9, Bliss: 20.8, Loewe: 26.1) δ scoring levels, with a 74.4% CSS score as efficacy metric Three additional combinations (BMS-536924 + AKTIV; temsirolimus + AKTIV; and BMS-754807 + tyrphostin AG-1478) showed synergistic effects at the MSA level (exceeding the 5-score threshold), while only one (BMS-754807 + AKTIV) showed additive effects. The contour plots of the 2D drug combination experiments are shown in Fig 5. The corresponding overall synergy (δ) scores and MSA δ scores from SynergyFinder3 for all the GRR combinations are shown in Table 3. All the remaining LWAS and GRR combinations not shown had δ < 5 for all models at both the overall and MSA level. In this study, we distinguish between synergy and potentiation when interpreting combination effects. We use “synergy” to indicate that both agents have measurable single-agent activity and that the observed combination response exceeds the expected additive effect under a defined reference model, whereas “potentiation” describes situations in which one agent shows minimal intrinsic activity in a given assay format but enhances the effect of the active drug/compound when combined [63, 64, 66]. This is particularly relevant for 3D spheroid assays, where we observed limited single-agent activity for temsirolimus; therefore, the enhanced growth inhibition seen with temsirolimus + AKTIV in 3D is interpreted primarily as potentiation of AKTIV rather than strict two-active-agent synergy, even when some synergy metrics are elevated.

### Evaluation of selected GRR-derived drug combinations for effects on SUM149 spheroids

To more accurately mimic IBC tumors, we utilized SUM149 spheroids as a 3D tumor model [44, 67]. In this assay, the SUM149 spheroids were allowed to form over 72 h before adding each of the three selected drug combinations identified from the 2D screen. After an additional 72 h of treatment, cell viability was then evaluated using CellTiter-Glo, an ATP-based luminescence assay. To ensure consistency and allow direct comparison between 2D and 3D experiments, we used the same 8 × 8 dose-response matrices that were used in our 2D experiments.

**Fig 6:**
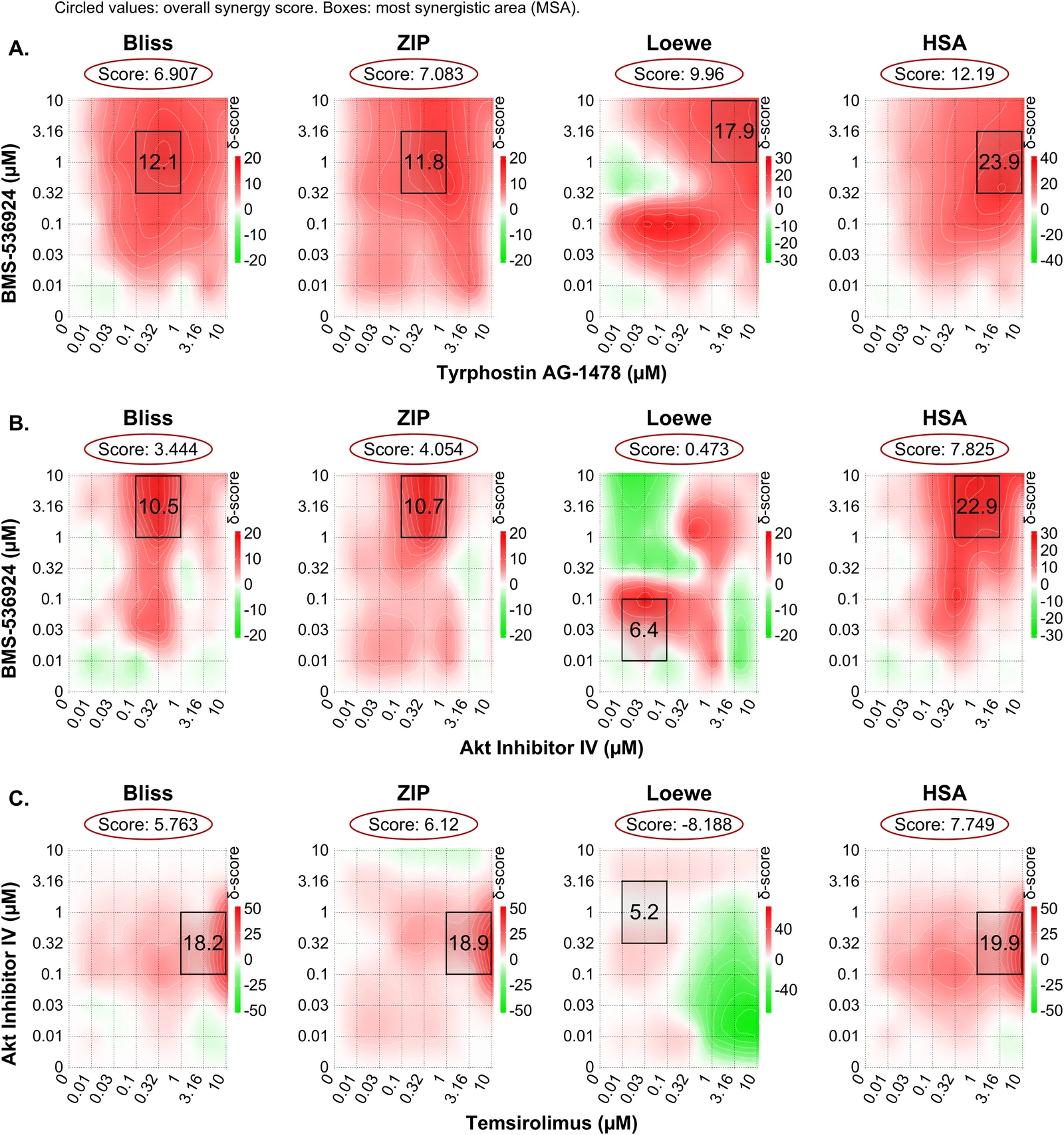
Analysis of drug combinations in 3D SUM149 spheroid model. Contour plots and their synergy scores across Bliss, ZIP, Loewe and HSA reference models obtained from SynergyFinder3. (A) BMS-536924 + tyrphostin AG 1478 (B) BMS-536924 + AKTIV and (C) Temsirolimus + AKTIV combinations. In each plot, x and y axes represent drug concentrations. Each 8×8 dose-response matrix consists of ½ log serial dilutions starting at 10 µM. Red areas indicate synergy, while green areas indicate antagonism. Black boxes highlight the MSA, with scores shown. Red circles in the top right corner of each plot indicate the overall δ score. Color scales on the right of each plot show the range of synergy scores. Data represent the average of three independent experiments (n = 3).

In our 3D spheroid study the BMS-536924 and tyrphostin AG 1478 combination yielded overall synergy scores of (ZIP: 7.1, HSA: 12.2, Bliss: 6.9, and Loewe: 9.9), while MSA level scores were (ZIP: 11.8, HSA: 23.9, Bliss: 12.1, and Loewe: 17.9). BMS-536924 and AKTIV combination showed overall synergy scores of (ZIP: 4.1, Loewe: 0.5, Bliss: 3.4, and HSA: 7.8), with MSA level scores reaching (ZIP: 10.7, Loewe: 6.4, Bliss: 10.5, and HSA: 22.9). Lastly, for the temsirolimus and AKTIV combination, overall synergy scores were (ZIP: 6.1, Loewe: −8.2, Bliss: 5.8, and HSA: 7.7), with MSA level scores of (ZIP: 18.9, Loewe: 5.2, Bliss: 18.2, and HSA: 19.9). The corresponding contour plots are shown in Fig 6. Based on the accepted criteria of a synergy score exceeding 5 across all reference models [65], these results indicate a synergistic effect at the MSA level for all tested combinations. Notably, as in the 2D study, the BMS-536924 and tyrphostin AG 1478 combination also achieved synergy at the overall level in the 3D study. It is worth noting that the Loewe model yielded a negative synergy score for the combination of temsirolimus and AKTIV. This result may be attributed to the fact that the Loewe model relies on well-defined dose-response curves for each agent [63, 64], a condition not satisfied by temsirolimus in our 3D assay, where it failed to produce a consistent dose–response relationship.

SynergyFinder+ was used to analyze the synergy scores and inhibition percentages (%) based on percent-effect of DMSO control at specific concentrations. There was good agreement between our 3D and 2D results, with comparable synergy scores across the four reference models (Table 4). Combination index (CI) values were also reported as an additional synergy metric to compare the three combinations between 2D and 3D models (Table 4). For the inhibition comparison, the BMS-536924 (1 µM) and tyrphostin AG 1478 (0.1 µM) combination showed nearly identical % inhibition in both 2D (78.6%) and 3D (79.1%) models. The BMS-536924 (1 µM) and AKTIV (1 µM) combination was more effective in 3D, achieving 92.3% inhibition compared to 79.4% in 2D. The temsirolimus (10 µM) and AKTIV (0.1 µM) combination achieved 61.2% inhibition in the 3D model, which is comparable to the 54.2% inhibition observed in 2D (S6 Fig). The 3D results also revealed stronger inhibitory effects from the combinations than from single agents. The combination of BMS-536924 (1 µM) with Tyrphostin AG 1478 (0.1 µM) achieved 79.1% inhibition, which is higher than each agent alone (BMS-536924: 66.6%; tyrphostin AG-1478: 3.7%). Similarly, combining BMS-536924 (1 µM) with AKTIV (1 µM) resulted in 92.3% inhibition, exceeding the effects of the individual treatments (BMS-536924: 65.1%; AKTIV: 73.1%). While the temsirolimus (10 µM) and AKTIV (0.1 µM) combination showed the lowest inhibition (61.2%), it still demonstrated improved efficacy compared to single-agent treatments (temsirolimus: −12.9%; AKTIV: 16.4%). Although the synergy metrics yielded high scores for this combination (**Table 4**), those values can be misleading in the 3D context as these calculations assume both drugs retain intrinsic activity, where their combined effect should be greater than the additive expectation. However, temsirolimus showed minimal to no activity in 3D; therefore, this interaction might be better described as enhancement or potentiation of AKTIV activity, which increased growth inhibition from 16.4% with AKTIV alone to 61.2% in combination.

**Table 4:** Summary table of synergy scores in SUM149 2D and 3D cell models.

Drug A	Drug B	Inhibition (%)						ZIP Score		HSA Score		Bliss Score		Loewe Score		CI_mean_ ± CI_sem_	
		2D			3D												
		Com	A	B	Com	A	B	2D	3D	2D	3D	2D	3D	2D	3D	2D	3D
**BMS-536924** **(1 µM)**	**Tyrphostin AG-1478 (0.1 µM)**	79	30	28	79	67	3.7	31	12	49	13	30	11	34	15	0.14 ± 0.01	0.03 ± 0.02
**BMS-536924** **(1 µM)**	**AKTIV** **(1 µM)**	79	27	48	92	65	73	12	6	32	19	17	1.6	19	19	0.45 ± 0.03	0.11 ± 0.02
**AKTIV** **(0.1 µM)**	**Temsirolimus** **(10 µM)**	54	6.5	39	61	16	13	9.6	46	15	45	11	56	16	42	0.14 ± 0.01	0.17 ± 0.01

Data were derived from bar plots generated by SynergyFinder+ software and represent the average of three independent experiments (S6 Fig). The table shows the percent inhibition for the drug combinations (Com), and drugs alone at their respective concentrations, along with the synergy scores calculated using four different models: ZIP, HSA, Bliss, and Loewe for each combination. All synergy scores above five indicate a synergistic effect, with higher values suggesting stronger synergy. The combination index (CI_mean_ ± CI_sem_) values are also reported as another synergy metric. CI < 1 (synergy), CI = 1 (Additive) and CI > 1 (Antagonism).

**Fig 7:**
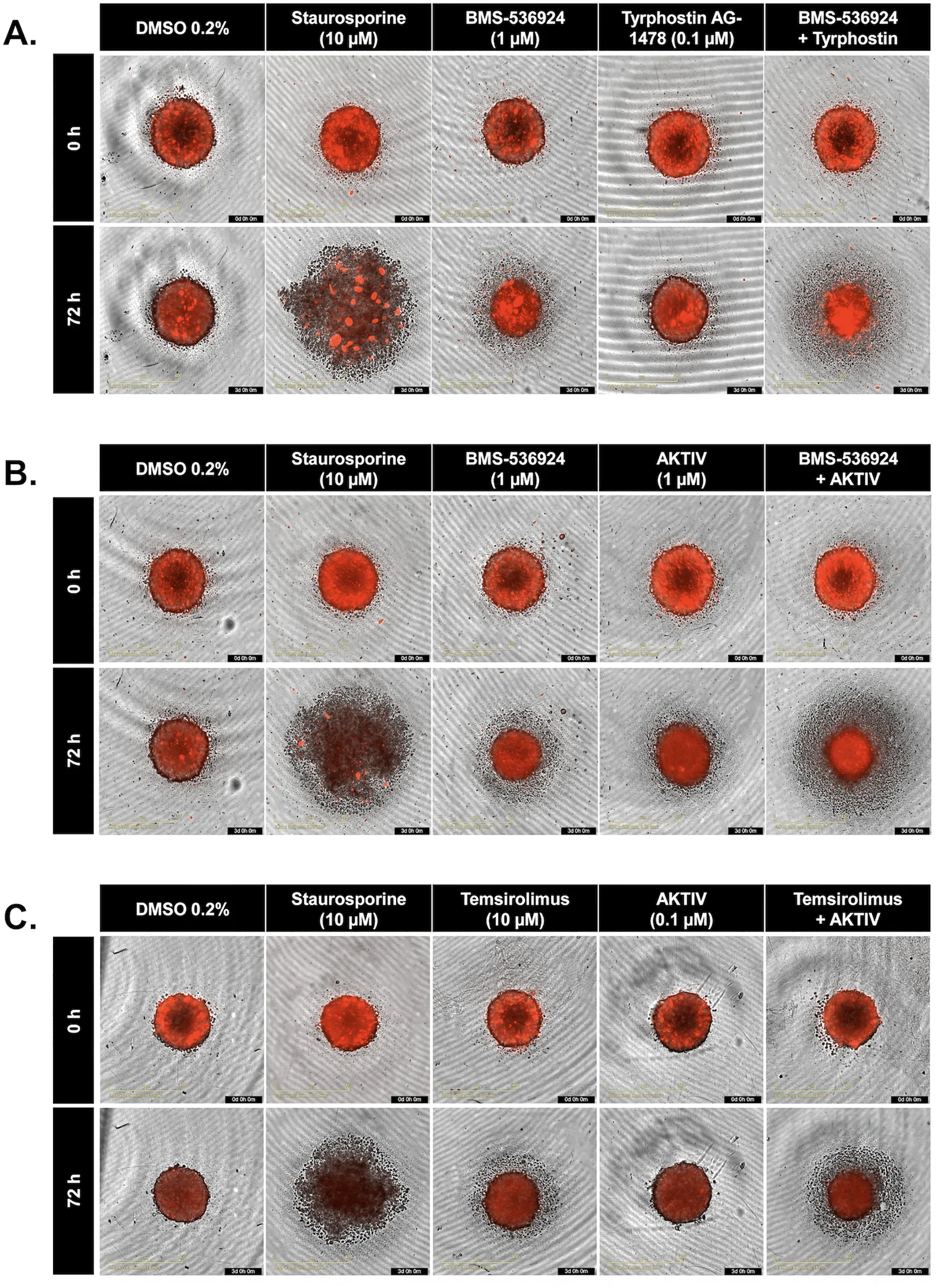
Representative SUM149 spheroids showing the effects of individual drugs and drug combinations. (A) BMS-536924 (1 µM) and tyrphostin AG-1478 (0.1 µM) combination. (B) BMS-536924 (1 µM) and AKTIV (1 µM) combination. (C) Temsirolimus (10 µM) and AKTIV (0.1 µM) combination. In each set, the top row shows spheroids at 0 h, while the bottom row shows the same spheroids after 72 h of treatment. DMSO 0.2% represents a negative control while staurosporine (10 μM) was used as a positive control.

We next used the SYTOX™ Red Dead Cell Stain and IncuCyte imaging system to visualize treatment-induced changes in SUM149 spheroid morphology and integrity. In all experimental panels, 0.2% DMSO was used as a negative (solvent) control, while staurosporine (10 μM) was used as a positive inhibitor control [67], which, as expected, caused significant spheroid disruption after 72 hours of treatment. The effects of individual drugs and their combinations are shown in Fig 7. Some combinations, especially those involving BMS-536924, appear to induce more pronounced changes in spheroid morphology compared to single-agent treatments. The images reveal varying degrees of spheroid integrity and morphological alterations in response to combination treatment.

Taken together, the drugs we identified from both the 2D and 3D combination screening pipeline included inhibitors BMS-536924 [68] targeting IGF-1R, tyrphostin AG-1478 [69, 70] targeting EGFR, AKTIV [71] targeting Akt phosphorylation, and temsirolimus [72] targeting mTOR.

### Assessment of selected GRR-derived drug combinations on SUM149 motility

To evaluate the identified drugs for effects on cell migration, a critical parameter in cancer cell behavior and metastasis, we used the “wound healing” assay, utilizing the precision WoundMaker tool (Essen BioScience). For this assay, a wound is created within a confluent cell monolayer, and cell movement back into the wound measured in real time using automated IncuCyte imaging. Cells were plated overnight and then the “wound” was created, followed by selected combination drug treatments (as listed in Table 4). Relative Wound Density (RWD) %, which considers any effects of cell proliferation, was calculated to quantify cell movement into the wound area relative to initial wound size over 24 h.

**Fig 8:**
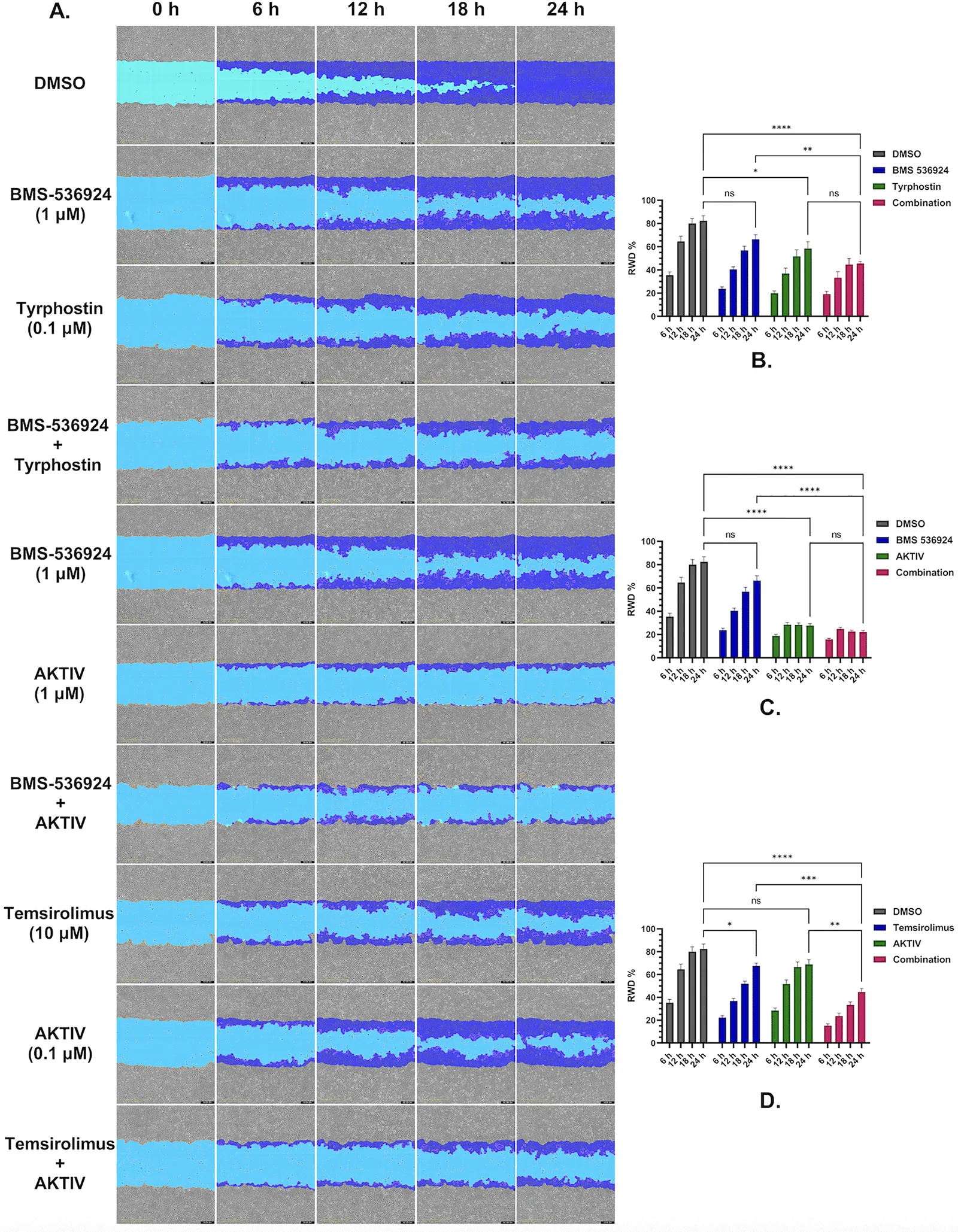
Effects of selected GRR-derived drugs on SUM149 motility. Relative wound density (RWD %) was measured at 6 h intervals from 0 to 24 h. The effects of individual drugs and their combinations are compared to the DMSO control. (A) Representative images of wound closure over 24 h treatment for each condition. (B) to (D) Quantification of the RWD % relative to each combination. (B) BMS 536924 (1 µM) and tyrphostin AG 1478 (0.1 µM). (C) BMS 536924 (1 µM) and AKTIV (1 µM). (D) Temsirolimus (10 µM) and AKTIV (0.1 µM). Statistical significance is indicated by asterisks (*p < 0.05, **p < 0.01, ***p < 0.001, ****p < 0.0001, ns: not significant). Error bars represent mean ± SEM from eight technical replicates.

The DMSO vehicle control demonstrated progressive wound closure over the 24 h observation period (shown in Fig 8). SUM149 treatment with the IGF-1R inhibitor BMS-536924 (1 μM) did not result in any significant effect on migration kinetics compared to control (*p* > 0.05), while treatment with the EGFR inhibitor tyrphostin AG-1478 (0.1 μM) had a significant effect on slowing wound closure with *p* < 0.05. When both BMS-536924 and tyrphostin AG-1478 were combined at those concentrations, migration was significantly reduced by 30% at 24 h (*p* < 0.001). The combination treatment was not significantly different from tyrphostin AG-1478 treatment alone. SUM149 cells treated with AKTIV (1 μM) had significantly impaired wound closure, reducing migration by > 50% compared to control at 24 h (p < 0.001). When both BMS-536924 and AKTIV were combined at these concentrations, migration was reduced by 30% at 24 h (*p* < 0.001). The combination treatment was not significantly different from AKTIV treatment alone. Treatment of SUM149 cells with temsirolimus (10 μM), an mTOR complex 1 inhibitor, significantly reduced migration by ~ 40% relative to control at 24 h (p < 0.05). The combination of temsirolimus and low dose AKTIV (0.1 μM) did produce significant effects (p < 0.001) compared to the control and single treatments. Representative wound healing images and corresponding statistical analyses are shown in Fig 8, with each experimental condition performed in eight replicates (n = 8).

### Selected GRR-derived combinations reduce phosphorylation of pathway proteins

**Fig 9:**
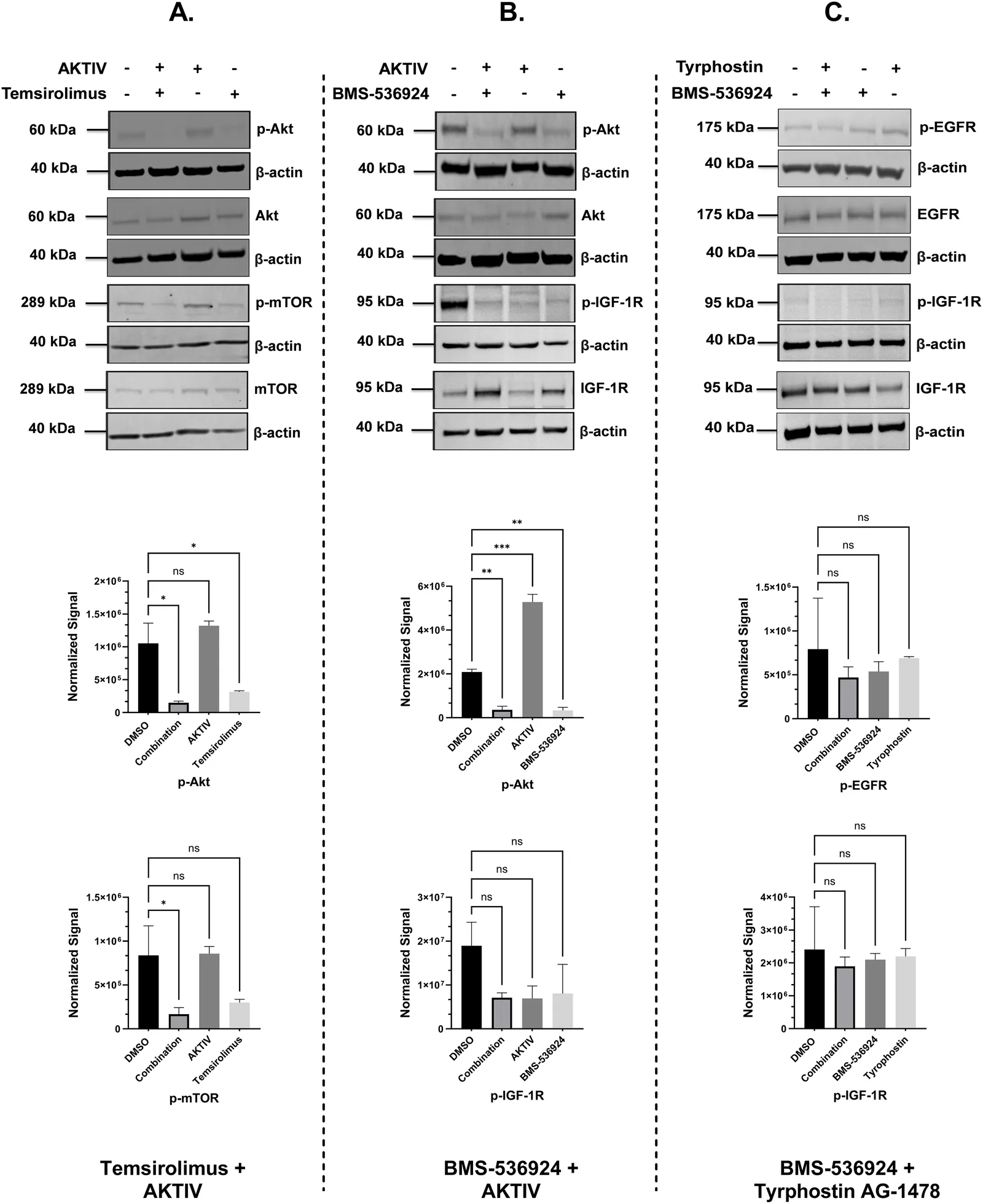
Western blot analysis of protein expression levels in response to the tested GRR-derived drugs and combinations. Representative western blots *and* densitometric *analysis* for (A) Temsirolimus (10 µM) and AKTIV (0.1 µM) combination effects on the expression of p-Akt, total Akt, p-mTOR, and total mTOR; (B) AKTIV (1 µM) and BMS-536924 (1 µM) combination effects on the expression of p-Akt, total Akt, p-IGF-1R, and total IGF-1R; and (C) Tyrphostin AG-1478 (0.1 µM) and BMS-536924 (1 µM) combination effects on the expression of p-EGFR, total EGFR, p-IGF-1R, and total IGF-1R. The presence (+) or absence (-) of each treatment is indicated, with β-actin serving as a loading control. All band intensities were normalized and quantified relative to the control. The uncropped blot images for all western blot experiments in Fig 9 are provided in the Supporting Information as S9 Fig. Representative western blots and densitometric analysis of triplicate samples (n = 3) for the drug combinations are shown in **S7 Fig**.

We next evaluated the effects of the selected three drug combinations on key signaling proteins in SUM149 cells. The analysis focused on expression levels and phosphorylation status of Akt, EGFR, IGF-1R, and mTOR proteins in response to single- and combination-drug treatments. Western blot analysis was performed after 24 h of drug treatment, and densitometric analysis for all treatment conditions, including individual drugs and corresponding combinations, and representative blots are presented in Fig 9 (uncropped blots are in the S9 Fig). Densitometric analyses of triplicate experiments (representative whole blots shown in S7 Fig) confirmed the statistical significance of these phosphorylation changes, with β-actin serving as the loading control across all conditions. The maintenance of total protein levels across treatments suggests that the observed effects were specific to protein phosphorylation rather than overall protein expression. Li-COR fluorescence detection was used for most targets to enable quantitative multiplexing and a broad linear range. However, IGF-1R and p-IGF-1R detection gave suboptimal signal-to-noise under Li-COR conditions; therefore, HRP-tagged secondary antibodies with iBRIGHT imaging was used for these proteins to improve sensitivity and band detection, while maintaining consistent sample preparation and loading controls across experiments (S7 Fig). All four of the proteins targeted by our selected GRR combinations were detectable in the SUM149 IBC cell line by western blot (Fig 9), and the drug combinations effectively suppressed their target pathways (Fig 9 and S7 Fig). The combination of AKTIV (0.1 µM) with temsirolimus (10 µM) demonstrated the strongest inhibitory effects, achieving a significant reduction of both phosphorylated Akt (p-Akt) (*p* < 0.001) and p-mTOR (*p* < 0.01) (Fig 9A). The second combination, AKTIV (1 µM) with BMS-536924 (1 µM), showed a significant decrease in p-Akt levels (*p* < 0.01) (Fig 9B), which appeared to be primarily driven by BMS-536924 rather than AKTIV. The combination of tyrphostin AG-1478 (0.1 µM) and BMS-536924 (1 µM) resulted in a reduction in both phosphorylated EGFR (p-EGFR) levels and phosphorylated IGF-1R (p-IGF-1R), but did not meet statistical significance, while maintaining stable total protein levels for EGFR and IGF-1R (Fig 9C).

### Assessment of differential gene expression changes in SUM149 cells treated with the selected GRR-derived combinations

**Fig 10:**
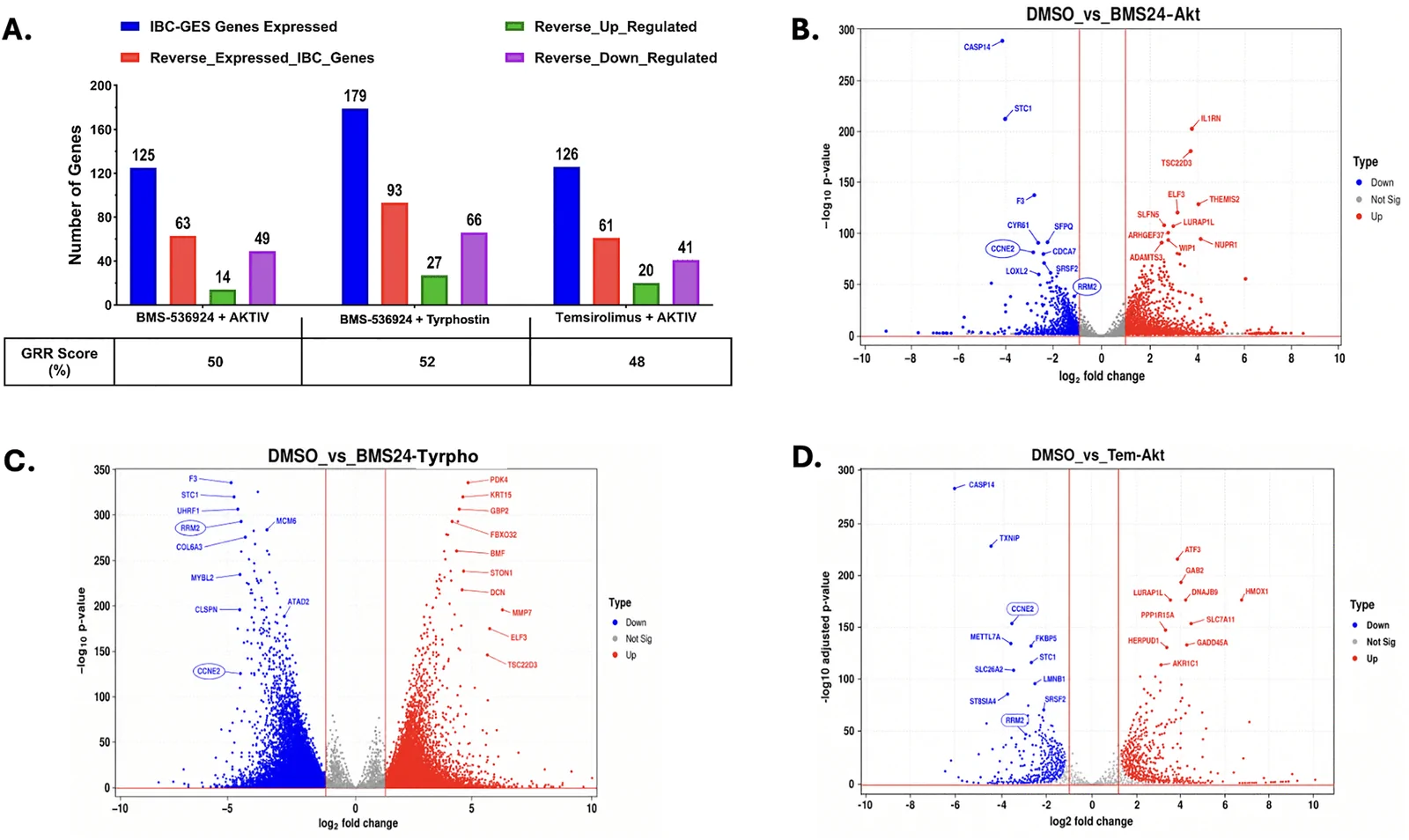
IBC gene expression changes induced by GRR-derived combinations in SUM149 cells. SUM149 cells were incubated with GRR combinations or DMSO alone for 24 h, and RNA isolated for RNA-Seq analysis (n = 3 biological replicates). A. Data were analyzed to identify IBC-GES genes significantly expressed in SUM149 cells (*p*adj < 0.05). Data are plotted as the number of IBC-GES genes: detected in SUM149 cells (blue columns), number of detected IBC-GES genes reversed by the combination treatment (orange columns), and the number reversed up (green columns) and the number reversed down (purple columns). The GRR score is the percentage of genes reversed/genes expressed. Left: DMSO (0.2%) vs (BMS‑536924 (1 μM) + AKTIV (1 μM)); middle: DMSO (0.2%) vs (BMS‑536924 (1 μM)) + tyrphostin AG1478 (0.1 μM)); right: DMSO (0.2%) vs (AKTIV (0.1 μM) + temsirolimus (10 μM)). **B-D***.* Volcano plots displaying log2 fold change (x‑axis) versus −log10 *p* value (y‑axis) for all genes differentially expressed in the combination treatments relative to the DMSO control; vertical red lines mark the fold‑change cutoffs, and the horizontal line denotes the significance threshold (FDR-adjusted *p* < 0.05). Red points indicate upregulated transcripts, and blue points indicate downregulated transcripts; representative top hits are labeled, with several clinically relevant genes highlighted by circles. **B**: DMSO (0.2%) vs (BMS‑536924 (1 μM) + AKTIV (1 μM)); **C**: DMSO (0.2%) vs (BMS‑536924 (1 μM)) + tyrphostin AG1478 (0.1 μM)); **D**: DMSO (0.2%) vs (AKTIV (0.1 μM) + temsirolimus (10 μM)).

To assess whether the selected GRR-derived combinations we identified herein with efficacy and the highest synergy scores (see **Table 4**) also reversed IBC-associated transcription, we performed RNA‑Seq analysis in SUM149 cells. For this, SUM149 cells were cultured and treated for 24 h (n = 3) with the selected GRR combinations, as well as DMSO vehicle control. RNA was isolated and RNA-Seq conducted as in materials and methods. Analysis of the expression of the 297 IBC-GES genes in SUM149 cells treated with the three dual-drug combinations revealed distinct transcriptional reversal patterns. For those IBC-GES genes detected as significantly expressed (*p*adj < 0.05) in SUM149 cells (blue columns in Fig 10A), the combinations BMS-536924 + AKTIV, BMS-536924 + tyrphostin, and temsirolimus + AKTIV, reversed 50%, 52% and 48% of the IBC-GES genes detected, respectively (orange columns Fig 10A). Beyond those genes in the IBC-GES, all three combination treatments induced significant global gene expression changes in SUM149, as shown in heatmaps (S8 Fig) and volcano plots (Fig 10). Relative to DMSO, the BMS-536924 (1 µM) + Tyrphostin AG 1478 (0.1 µM) combination significantly altered a total of 2868 genes (p < 0.05), with 1743 up-regulated (~ 61%) and 1125 down-regulated (~ 39%); the BMS-536924 (1 µM) + AKTIV (1 µM) combination significantly altered a total of 1982 genes with 1381 upregulated (~70%) and 601 downregulated (~30%), and the temsirolimus (10 µM) + AKTIV (0.1 µM) combination significantly altered a total of 2,434 genes with 1377 up-regulated (~ 57%) and 1057 down-regulated (~ 43%) (S8 Fig). Volcano plots highlighted several clinically relevant genes whose expression was significantly changed, e.g., *RRM2* and *CCNE2* were downregulated in all three combination treatments (Fig 10B-D).

## Discussion

Due to the limited availability of therapies targeting IBC, particularly for the triple-negative (TNBC) subtype, new therapeutic targets and drug combinations need to be identified to address its rapid progression and aggressive metastasis [2, 73]. Drug repurposing is one approach to identify therapies for rare diseases such as IBC [23, 25, 74], and we have previously used two computational approaches: text mining (LWAS) and gene expression reversal rate analysis (GRR), as tools for predicting novel drug candidates for IBC [28, 29].

To evaluate the predictive value of our computational approaches, we tested the predicted drugs and compounds for anti-proliferative efficacy in four breast cancer cell lines, including the SUM149 IBC model. The drugs predicted from our previous oncology-focused LWAS study [28], showed a high success rate (70%) *in vitro*, with 17 of 24 compounds demonstrating efficacy in SUM149 cells, and 15 of the 17 also active in the other cell lines. For our previous GRR study, which had identified both non-oncology and oncology drugs [29], the success rate *in vitro* was lower, with 6 out of 16 compounds (~38%) showing efficacy in the cell line models. Among the LWAS-predicted drugs for IBC, paclitaxel, docetaxel, and doxorubicin showed the highest efficacy with IC_50_ potencies for SUM149 in the nanomolar range (Tables 1 and 5). These drugs are FDA-approved for breast cancer and are also among those used as IBC treatment options [15]. All 11 of the LWAS drugs that have been tested in clinical trials for IBC (S1 Table), showed efficacy in our SUM149 nuclear-count assay. Of the other 13 LWAS drugs/compounds not yet tested in clinical trials for IBC (S1 Table), 6 demonstrated activity in SUM149 (46% success), while 7 showed no activity. For the 7 inactive LWAS compounds, we did not identify prior reports of activity in IBC models. Four novel LWAS-predicted drug candidates with *in vitro* efficacy were identified, each of which, to our knowledge, had not previously been investigated for IBC (as determined from a review of PubMed and clinicaltrials.gov (S1 Table)). These included topotecan, a topoisomerase I inhibitor, and cytarabine, an antimetabolite, which had potency across all tested cell lines, including IC_50_ values of 0.008 µM and 0.025 µM in SUM149, respectively. In our initial computational LWAS analysis [28], topotecan appeared among the drugs linked to medulloblastoma, while cytarabine was associated with acute lymphocytic leukemias, both diseases that clustered closely to IBC [28]. This semantic co-occurrence analysis suggested that other drugs targeting these “IBC-clustered” diseases, such as daunorubicin and vincristine, would also have activity in IBC models, and we did observe efficacy for these two drugs in our anti-proliferative assay (Tables 1 and 5).

**Table 5:** Clinical status of LWAS-predicted drug candidates in IBC.

Drug Class	Drug Name ^a^	Activity in SUM149 ^b^	Clinical Trials for IBC ^c^	Treatment Option for IBC ^d^
Vinca Alkaloids	Vincristine	+ + + +	No	No
	Vinorelbine	+ +	Yes	Yes
Antimetabolites	Methotrexate	+	Yes	Yes
	5-Fluorouracil	+	Yes	Yes
	Gemcitabine	+ + + +	Yes	Yes
	Hydroxyurea	–	No	No
	Cytarabine	+ + +	No	No
Alkylating Agents	Carmustine	–	No	No
	Ifosfamide	–	No	No
	Dacarbazine	–	No	No
	Carboplatin	+	Yes	Yes
Anthracyclines	Daunorubicin	+ + + +	No	No
	Doxorubicin	+ + + +	Yes	**Yes**
Topoisomerase Inhibitors	Etoposide	+ + +	Yes	No
	Mitoxantrone	+ + + +	No	No
	Topotecan	+ + + +	No	No
Taxanes	Paclitaxel	+ + + +	Yes	**Yes**
	Docetaxel	+ + + +	Yes	**Yes**
Kinase Inhibitors	Gefitinib	+ + +	No	No
	Imatinib	–	No	No
	Sunitinib	+ +	Yes	No
	Lapatinib	+ +	Yes	Yes
Peptide drug	Octreotide	–	No	No
Glucocorticoid	Prednisone	–	No	No

^a^From Ji et al. 2020 [28]. ^b^ Activity data for drug in SUM149 (based on Table 1 in the current study; from minimal [-] to high activity [++++]. ^c^ Tested in clinical trials (clinicaltrials.gov). ^d^ American Cancer Society, Chainitikun et al. [18], and MD Anderson Cancer Center’s IBC Treatment Algorithm [59].

The tyrosine kinase inhibitors (TKI) lapatinib, gefitinib, and sunitinib showed variable potency and efficacy in SUM149 cells, with gefitinib having the lowest IC_50_ value of 0.4 µM, and lapatinib produced the greatest maximal inhibition (~ 90% at 10 µM). These two drugs inhibit EGFR, which is highly expressed in the SUM149 cell line [75, 76], and along with the finding that EGFR signaling plays a role in IBC progression and metastasis [77], targeting EGFR pathway dependence may contribute to the activity of these agents. While lapatinib has been tested clinically for IBC and showed limited success [78, 79], adverse events and toxicity were observed [18, 80], and there are currently no ongoing clinical trials for lapatinib in IBC.

Our evaluation of the GRR-derived list [29] identified novel effective drugs not previously tested in IBC models, primarily TKIs and pathway-targeting inhibitors. Notably, tipifarnib, a farnesyltransferase inhibitor (FTI), demonstrated the highest potency (IC_50_ = 0.08 μM) in SUM149. This finding aligns with the contributing role of RhoC GTPase, a Ras superfamily member, in IBC pathogenesis [81, 82], since RhoC activation requires farnesylation, a post-translational modification, it can be targeted by FTIs. Tipifarnib has also progressed to Phase II clinical trials in locally advanced breast cancer (LABC), both as monotherapy and in combination with hormonal therapy [81, 82]. These results validate our approach and support the potential of other GRR effective compounds as repurposed therapeutic candidates for IBC treatment.

**Fig 11:**
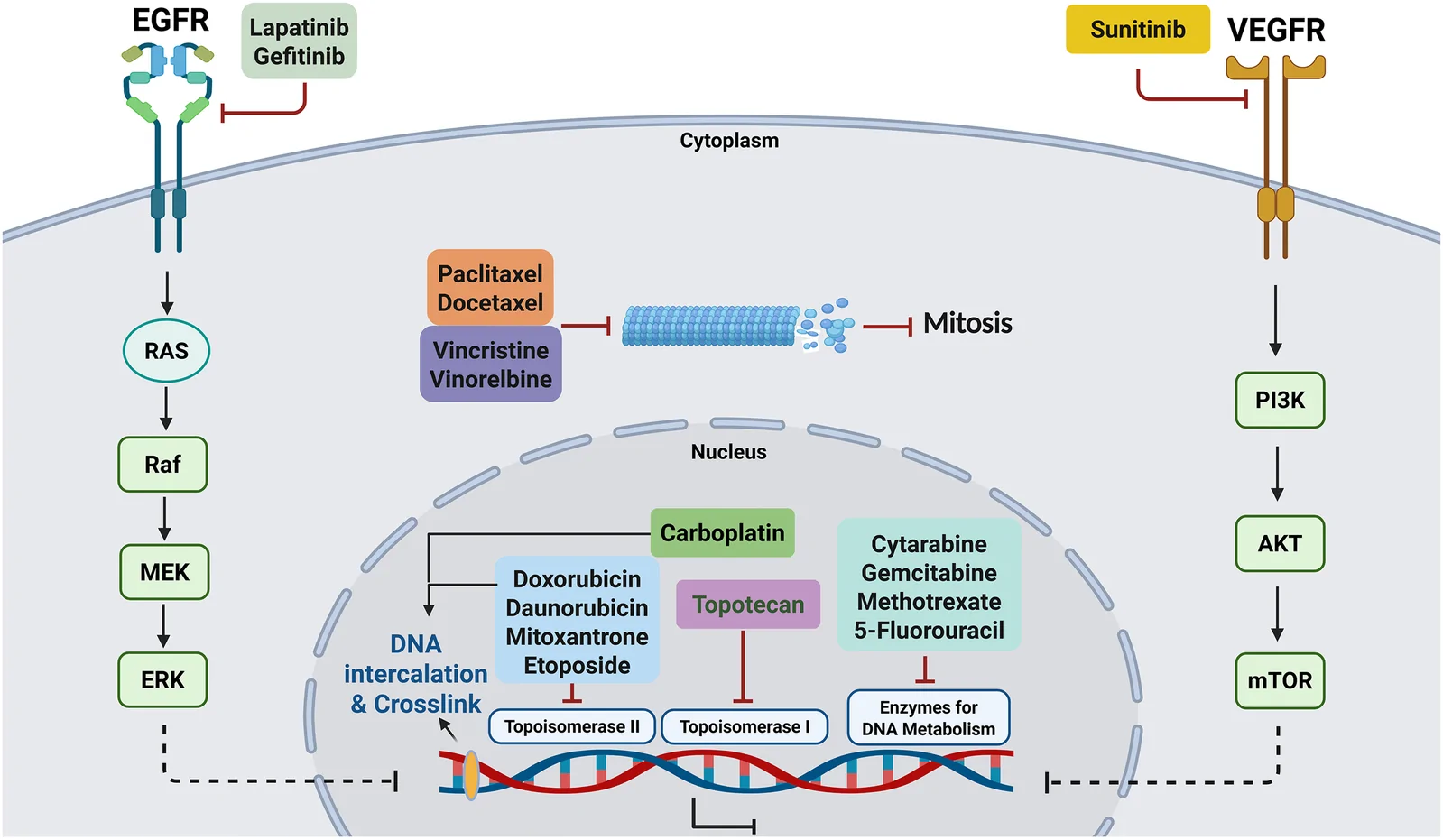
Cellular signaling pathways targeted by the LWAS-predicted drugs with anti-proliferative efficacy. The 17 LWAS-predicted drugs with anti-proliferative efficacy represent diverse drug classes. These include targeted therapies such as TKIs and various chemotherapeutic agents categorized by mechanism: microtubule-targeting drugs, DNA-damaging agents (e.g., topoisomerase I and II inhibitors), platinum-based drugs, and antimetabolites.

**Fig 12:**
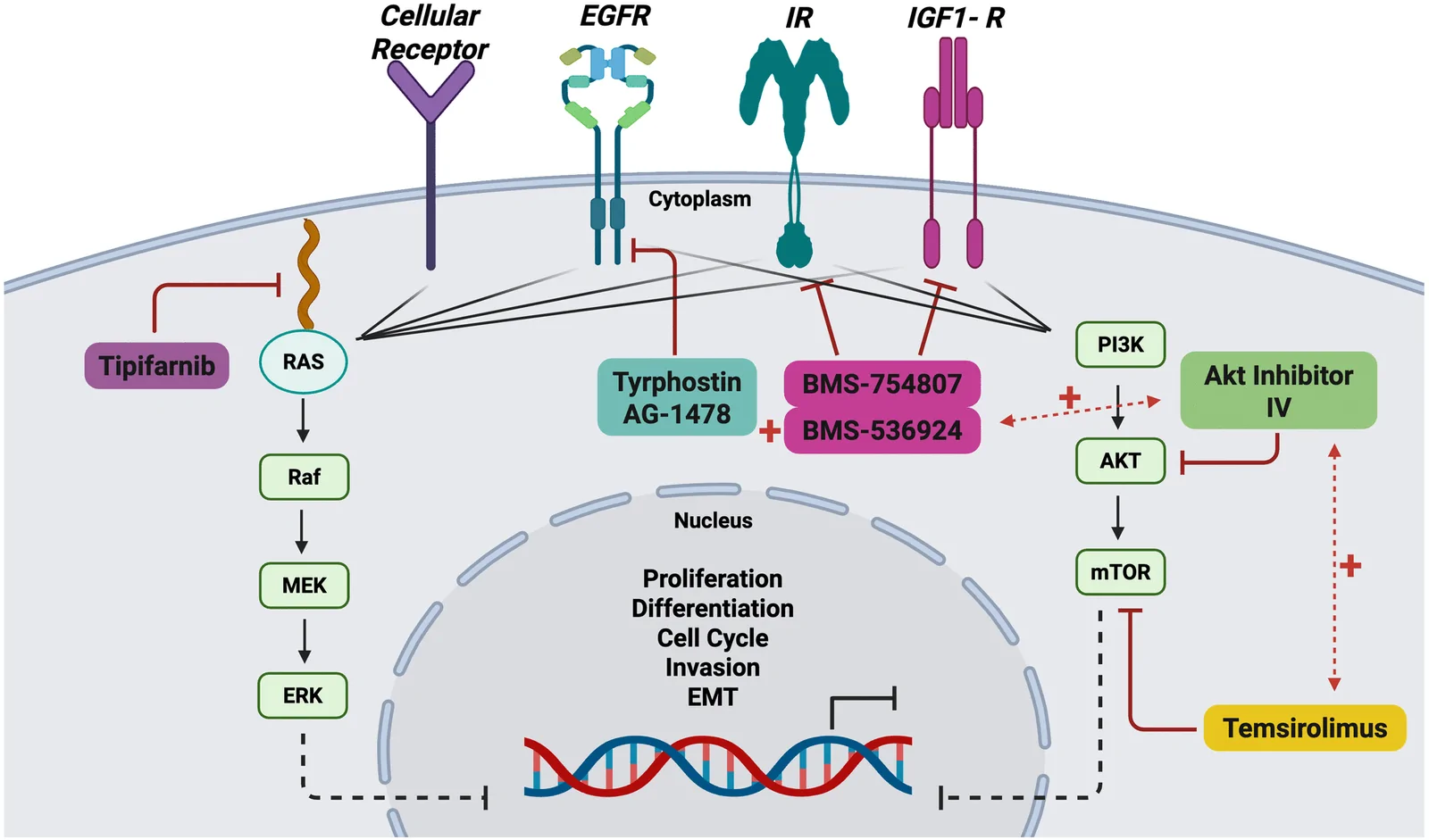
Cellular signaling pathways targeted by the GRR-predicted drugs with anti-proliferative efficacy and the synergistic combinations. The 6 GRR-predicted drugs with anti-proliferative efficacy are all TKIs, targeting various receptors and their downstream signaling pathways such as RAS/RAF/MEK/ERK and PI3K/AKT/mTOR. These pathways regulate critical cellular processes, including proliferation, differentiation, cell cycle, invasion, and epithelial-mesenchymal transition (EMT). The three synergistic combinations (tyrphostin + BMS536924; BMS536924 + AKTIV, and AKTIV + temsirolimus) highlighted with red plus signs and dashed red arrows.

The IGF-1R inhibitors BMS-754807 and BMS-536924 showed IC_50_ values of 0.5 μM and 1.9 μM, respectively in the Hoechst nuclear-count assay. The differences in potency may reflect a more selective binding profile of BMS-536924 compared to the broader BMS-754807 IGF-1R/IR inhibition profile [83, 84]. BMS-536924 has previously been shown to have anti-growth activity in SUM149 and MCF7 cells [85]. While these IGF-1R inhibitors have been tested in preclinical breast cancer models [84, 86] and in clinical trials for breast cancer [87], they remain unexplored in the clinic for IBC, suggesting potential novel therapeutic opportunities. Although there are no FDA-approved small-molecule inhibitors of IGF-1R, there is significant interest in the therapeutic targeting of IGF-1R in TNBC including combination approaches to overcome IGF-1R targeted therapy resistance [88]. In our study, temsirolimus, an mTORC1 inhibitor [72], showed a biphasic response in SUM149 with moderate potency for cytotoxicity (absolute IC_50_ = 4.3 μM), but a high potency for proliferation (relative IC_50_ = 0.1 nM). In both assay formats, temsirolimus was significantly more potent in the other three cell lines tested. Differences in sensitivity across the tested cell lines for temsirolimus may reflect differences in baseline pathway activation, feedback regulation, and the degree to which the target inhibition produces cytostatic versus cytotoxic effects in different cellular contexts. While FDA-approved for renal cell carcinoma and assessed in clinical trials for metastatic breast cancer [89], temsirolimus has not been specifically investigated for IBC treatment, presenting an additional therapeutic avenue worth exploring both as monotherapy or in combinations. Another mTOR inhibitor, everolimus, which did not show up in our GRR analysis, has been FDA-approved for some types of metastatic breast cancer [90, 91]. The clinical activity of everolimus in breast cancer was supported by the BOLERO-2 trial, in which everolimus plus exemestane (an aromatase inhibitor) demonstrated progression-free survival (PFS) in hormone receptor (HR)-positive advanced breast cancer [90]. In our study, tyrphostin AG 1478, an EGFR inhibitor [69, 70], and AKTIV [71, 92] showed comparable potency with IC_50_ values of 0.23 μM in SUM149 cells, yet neither has been evaluated for IBC treatment. These results suggest potential for expanding treatment options in IBC, with the possibility of evaluating additional clinically relevant EGFR and Akt pathway inhibitors, alone and in combination. The mechanisms of action for the effective LWAS and GRR candidates, along with their corresponding pathways, are illustrated in Fig 11 and Fig 12, respectively.

Because resistance to single-agent therapy can arise through compensatory pathway activation in cancer, we also examined drug combinations that targeted complementary signaling nodes. We first evaluated the most promising synergistic drug combinations identified in our initial screening in 2D SUM149 model and then in 3D SUM149 spheroids which provide a more architecture-dependent tumor model for drug response over the 2D model. [67]. We focused on SUM149 as it is a widely used IBC model [44, 45, 55-57], and we have shown it to be amenable to high-throughput 2D and 3D assay formats [44, 45, 67], allowing testing of multiple drug combinations. We used a high-throughput pipeline approach to first screen combinations at single dose and then in a more extensive 8 × 8 matrix (see Fig 3). A comparable approach to identify novel drug combinations with synergy for other cancers has been reported [93]. Our initial combinations were carried out by evaluating each drug at its EC_25_ concentration, with the Bliss method used to calculate Bliss excess scores. Bliss was used as it is typically better for assessing combinations of drugs that act through different pathways [94]. This resulted in identifying 25 combinations from the LWAS list and 9 combinations from the GRR list (*p* < 0.05). Then, we performed an 8 × 8 matrix analysis focusing on combinations with the highest Bliss excess scores. Our synergy analysis using SynergyFinder3 revealed three synergistic combinations meeting our stringent criteria, with scores greater than 5 across all reference models (Bliss, Loewe, HSA, and ZIP) at the MSA level. SynergyFinder+ was used to confirm these results at the micro visualization scale, with detailed bar plots presented in S6 Fig. The GRR-derived combinations produced the strongest synergy under our prespecified criteria (see Table 4). The four targeted agents represented in these combinations are BMS-536924, an IGF-1R/IR inhibitor [68], AKTIV, an Akt inhibitor [71, 92], tyrphostin AG-1478, an EGFR inhibitor [69, 70], and temsirolimus, an mTOR inhibitor [72]. Each of these drugs targets specific nodes within the PI3K/Akt/mTOR signaling cascade, a crucial signaling cascade in several cancers and an active area of clinical drug development [95–98]. Our synergyfinder+ analysis further supported the selection of three GRR-derived combinations prioritized by SynergyFinder3, by identifying specific concentration ranges within the 8 × 8 dose-response matrices that showed strong synergy (e.g., BMS-536924 (1 µM) and tyrphostin AG-1478 (0.1µM), BMS-536924 (1 µM) and AKTIV (1 µM) and temsirolimus (10 µM) and AKTIV (0.1 µM)). These concentration-specific effects guided the selection of the three GRR-derived combinations for subsequent validation.

The first combination of BMS-536924 (1 µM) and tyrphostin AG-1478 (0.1µM), targeting IGF-1R and EGFR, respectively, produced the strongest synergy in the primary screen with Bliss excess score = −0.266 and retained high synergy in the 2D and 3D secondary combination matrix screen (synergy scores summarized in **Table 4**). This combination primarily targets the crosstalk between EGFR and IGF-1R [99]. EGFR is expressed at high levels [100], and is activated in SUM149 cells [101]. High levels of IGF-1R can cause resistance across different cancers [87, 102], and cancer cells can become resistant to IGF-1R inhibitors, by activating PI3K/mTOR pathways through alternative EGFR signaling [103, 104]. It was also reported that the interaction between IGF-1R and different receptor tyrosine kinases (RTKs), such as EGFR, can create compensatory mechanisms that can limit treatment efficacy and promote resistance when targeting individual pathways [105]. These interactions occur through multiple mechanisms, including direct receptor associations and downstream signaling networks [106]. These compensatory mechanisms provide a strong rationale for dual targeting of both EGFR and IGF-1R, including in TNBC [107], with a consideration of reducing the cytotoxicity of the combined drugs by using the lowest concentrations that can achieve high synergy scores.

The IGF-1R/Akt signaling crosstalk represents a promising therapeutic target, addressed by our second identified drug combination of BMS-536924 (1 µM) and AKTIV (1 µM). Akt, also known as protein kinase B, mediates signal transduction from RTKs, including IGF-1R and IR, through the PI3K pathway. This signaling cascade promotes G1/S cell cycle transition, which is crucial for cellular proliferation [97, 108]. The significant overlap between IGF-1R and Akt signaling provides a mechanistic rationale for the simultaneous targeting of both molecules.

Our studies identified a third combination, temsirolimus (10 µM) and AKTIV (0.1 µM), that can simultaneously target mTOR and Akt. mTOR, a downstream effector, functions as a central regulatory kinase that controls multiple essential cellular survival processes through two different protein complexes: mTORC1 and mTORC2. Through these complexes, mTOR can execute its regulatory functions by phosphorylating key downstream proteins, including Akt [109, 110]. We suggest that targeting both proteins through our identified combination of temsirolimus, an mTORC1 inhibitor [72, 111], alongside AKTIV may effectively lower the development of drug resistance, while achieving more complete pathway suppression. The pan-AKT inhibitor, capivasertib was found to improve PFS when combined with fulvestrant in the CAPItello-291 trial and this combination was approved by the FDA for HR-positive/HER2-negative locally advanced or metastatic breast cancer with PIK3CA, AKT1, or PTEN alterations [112–114]. This approval validates Akt as a promising clinical target and supports our findings that Akt inhibitors, which appeared in two of the three identified combinations, could be potentially effective in treating IBC. Some recent clinical developments in breast cancer have targeted upstream of this pathway at PI3Kα, with PI3Kα inhibitors including alpelisib [115], and inavolisib [116], approved for PIK3CA-mutated, HR-positive/HER2-negative advanced breast cancer. For patients with this type of breast cancer, alpelisib showed clinical benefit in combination with fulvestrant in the phase 3 SOLAR-1 trial [117], and in a more recent trial, inavolisib plus palbociclib and fulvestrant improved PFS and overall survival [116]. Interestingly, SUM149 cells have been reported to be relatively resistant to alpelisib with an IC_50_ of ~ 3 µM [118].

We also tested these three selected combinations in 3D cell models. Although all these combinations showed synergistic effects at the proliferation level in 2D, the temsirolimus and AKTIV combination showed a significant shift in 3D cell models, where temsirolimus loses its single-agent efficacy. This difference in response may represent a mechanistic transition from classical synergy observed in 2D cultures (where both agents demonstrate independent activity) to a potentiator or enhancer effect in 3D models, as described previously [66]. While temsirolimus alone appeared to be ineffective, it significantly sensitized cells to AKTIV, enhancing the growth inhibition from 16.4% with AKTIV as a single agent, compared to 61.2% in the combination. Mechanistically, this transition may reflect fundamental aspects of 3D tumor biology, where cells in spheroids can activate survival pathways (e.g., mTOR), which has been reported to have a greater effect on survival in 3D than in 2D breast cancer cell cultures and may contribute to drug resistance [119]. This shift in response highlights the need for accurate modeling of tumor behavior and drug responses, making 3D cultures a valuable tool in cancer research and drug development. This could potentially be translated to enhance therapeutic efficacy in clinical applications, particularly in IBC, where these pathways are known to drive tumor growth and survival [2, 17].

To investigate how these drug combinations might affect IBC cell migration, we performed wound-healing assays to assess drug effects on cell motility. Our results demonstrated that the combinations of (BMS-536924 + tyrphostin AG-1478) and (temsirolimus + AKTIV) enhanced the inhibitory effects of the individual drugs on cell motility and migration. This aligns with studies reporting the role of the PI3K/Akt/mTOR pathway in cell motility/invasion, and its crosstalk with IGF-1R and EGFR signaling, either in SUM149 [100, 120] or in TNBC [121–124]. In our study, the combination co-targeting Akt upstream (by AKTIV) and mTORC1 downstream (by temsirolimus) may more completely attenuate the PI3K/Akt/mTOR axis and reduce any compensatory rebound activation. These results suggest that suppression of SUM149 motility may require inhibition of multiple signaling pathways.

We also assessed the GRR-derived combinations for direct effects on signaling pathways in SUM149 cells, and we observed that the combination of AKTIV and temsirolimus suppressed phosphorylation of both Akt and mTOR (Fig 9A). While temsirolimus appeared to be the primary driver of this suppression, the combination showed enhanced suppressive effects compared to control, indicating potential synergistic interactions within the Akt/mTOR signaling pathway. The combination of AKTIV and BMS-536924 also reduced Akt phosphorylation, with BMS-536924 appearing to contribute most strongly to this effect (Fig 9B). While, with the same combination, IGF-1R phosphorylation showed a downward trend, the change did not reach significance. This was also complicated by our observation that BMS-536924 appeared to increase total IGF-1R expression, perhaps indicating the activation of compensatory mechanisms in response to pathway inhibition, as reported previously [125, 126]. This compensatory signaling through alternative RTKs and/or changes in receptor abundance and localization is well described as a resistance mechanism for IGF-axis targeting and is relevant for dual IGF-1R/IR inhibitors [102, 106, 125]. In the specific BMS-536924 context, prior work has shown that HER-family signaling can cause resistance and provides a rationale for combining IGF-1R/IR inhibition with EGFR/HER2-targeted agents [99, 104]. Moreover, therapy-associated increases in IGF-1R expression have been observed in breast tumors, where membranous IGF-1R was upregulated in a subset of patients after neoadjuvant treatment and associated with poorer outcome [127]. This IGF-1R upregulation has been shown to sustain AKT/mTOR signaling and drive resistance to PI3K-pathway inhibition [128]. These findings suggest that the increase in total IGF-1R after BMS-536924 treatment may reflect a clinically relevant adaptive mechanism that could limit the durability of single-node inhibition and further supports rational combination strategies that block parallel RTKs and/or downstream PI3K–Akt–mTOR nodes [99, 104, 126]. While in cell-based efficacy assays, AKTIV demonstrated anti-proliferative effects with an IC_50_ of 0.2 µM, AKTIV at 0.1 µM or 1 µM did not appear to reduce p-Akt(Ser473) expression, perhaps due to differences in assay timing (24 h for western blot compared to 72 h for the cell assays), or Akt-independent effects. For example, AKTIV has been reported to accumulate in mitochondria and disrupt cellular bioenergetics, raising the possibility that some anti-proliferative effects may be mediated, at least in part, through Akt-independent mechanisms [92]. Further, phosphorylation dynamics are time-dependent, and compensatory feedback can restore pathway phosphorylation after initial inhibition. For example, Akt pathway inhibition can induce feedback upregulation/phosphorylation of multiple RTKs (including IGF-1R/IR) within 24 h [129]. Future studies using short- and long-term drug treatments, assessment of Akt Thr308 and Ser473 phosphorylation, downstream Akt substrates such as PRAS40 and GSK3β, and broader phospho-RTK/phospho-kinase profiling would help distinguish on-target pathway inhibition from adaptive rebound and/or off-target mechanisms.

The gene reversal approach involves identifying drugs that can reverse disease-associated gene expression signatures back to a ‘normal/healthy’ transcriptomic state [32]. In our previous computational GRR study [29], we had first generated a 387 IBC-gene expression signature (IBC-GES) compiled from published studies using IBC patient samples (see citations in [29]). 297 of these 387 genes had drug data in the LINCS database, and our GRR analysis [29] identified drug perturbations that significantly reversed the expression of genes in the IBC-GES list. For example, tyrphostin AG-1478 had a GRR score of 59% [29], indicating 175 of the 297 genes were reversed by that drug perturbation in LINCS. In our RNA-seq study herein, we observed that ~50% of the IBC-GES genes were reversed by the GRR combinations tested on SUM149, suggesting some reprogramming of IBC-associated transcriptional profiles. Without significant follow-up mechanistic and *in vivo* studies, it is challenging to assess the clinical significance of this *in vitro* reversal rate. A limitation of our GRR-based reversal scoring is that the gene coverage of the reference perturbational transcriptomic resource constrains it. As mentioned, of the 387 IBC-GES genes, 297 were represented in LINCS/L1000, a platform centered on 978 landmark transcripts [41, 42, 130], thus, reversal scoring is necessarily computed on this gene subset, which could introduce a selection bias toward pathways and genes that are well represented among these L1000 landmark/inferred genes.

The convergence of the GRR-derived hits on the IGF-1R/EGFR/PI3K-Akt-mTOR axis (Fig 12) may reflect both IBC-related pathway biology and potential LINCS/L1000 drug/target bias. EGFR and IGF-1R/IR can activate PI3K/Akt/mTOR signaling, pathways previously linked to IBC proliferation, migration, survival, and therapeutic resistance [2,17,67,100,120,131], and our phenotypic, protein-level, and RNA-seq data are consistent with drug effects on pathway-level activity. As our GRR scoring was limited to the 297 IBC-GES genes [29] and the LINCS drug perturbation data associated with these genes, our hits may be enriched for those targeting well-characterized, druggable canonical signaling pathways, including kinase pathways, which are a major focus of LINCS [130]. The availability of a broader drug-gene expression platform, in combination with our GRR analysis, may allow the identification of drugs targeting less well-characterized pathways.

To begin addressing these limitations, we also analyzed global transcriptional changes induced by the drug combination treatments. Analyzing global gene expression changes in our RNA-Seq data (Fig 10 and S8 Fig) found that compared with DMSO control, the BMS‑536924 + tyrphostin AG1478 group showed the strongest transcriptional response, with 2868 differentially expressed genes (DEGs), followed by AKTIV + temsirolimus with 2434 DEGs, and BMS‑536924 + AKTIV with 1982 DEGs (S8 Fig). Volcano (Fig 10B-D) and heatmap (S8 Fig) representations identified strong induction of *PDK4*, *MMP7*, *ELF3*, and *TSC22D3*, with marked repression of *RRM2*, *CCNE2*, *F3*, *UHRF1*, *RRM2*, and *STC1*. The BMS‑536924 + tyrphostin (Fig 10C) combination elicited the most extensive transcriptional response among the three drug combinations, reflecting the broad impact of dual IGF-1R/EGFR blockade. For example, both BMS-536924 + tyrphostin and the BMS-536924 + AKTIV treatments reduced expression of *RRM2*, a key driver of DNA synthesis and proliferation. *RRM2*, which encodes ribonucleotide reductase, is overexpressed in aggressive basal-like and TNBC, where elevated levels correlate with adverse clinical features including larger tumor burden, lymph node involvement, and disease recurrence [132, 133]. The common therapeutic target in these combinations is IGF-1R, which is inhibited by BMS-536924. Previous studies have reported that inhibition of IGF-1R induces replication stress by downregulating RRM2 expression through AKT/MEK-ERK signaling, thereby compromising ribonucleotide reductase activity and dNTP pools, a phenotype rescued by RRM2 overexpression [133, 134]. These combinations also downregulated the *F3* gene that encodes for tissue factor (TF), a pro-coagulant protein linking cancer to thrombosis and inflammation [135, 136]. TF usually accumulates at invasive tumor fronts and promotes hematogenous metastasis and tumor-associated coagulopathy [137, 138]. Its suppression may attenuate the microembolic phenotype distinctive to IBC, in which clusters of tumor cells (tumor emboli) are found in blood vessels [139], and more distinctly within dermal lymphatics [140]. Additionally, the BMS-536924 + tyrphostin combination downregulated *UHRF1*, an epigenetic modifier controlling DNA methylation [141]. UHRF1 is usually upregulated in high-grade breast cancers and helps silence tumor suppressors via DNA methylation (e.g., promoting cell cycle progression and evasion of apoptosis) [142, 143]. The observed downregulation of this gene in drug-treated SUM149 cells suggests an effective shutdown of proliferative signaling in IBC.

Cyclin E2 (*CCNE2*) was downregulated by two combinations: BMS-536924 + AKTIV (Fig 10B) and AKTIV + temsirolimus (Fig 10D). CCNE2, an S phase cyclin, is frequently overexpressed in breast cancer [144], and prior studies have reported the overexpression of Cyclin E, including CCNE2, in IBC, and associated this pattern with poor prognosis in IBC patients [145]. In our RNA-Seq study, *CASP14* (Caspase 14) was also downregulated by both of these combinations. CASP14 was previously found to be highly expressed in basal A breast cancer cell lines [146], and has also been reported to be a marker of aggressive tumor types and poor survival in TNBC patients [147, 148]. Both CCNE2 and CASP14 are linked to Akt, the common target in both these drug combinations, and active Akt signaling promotes cell-cycle progression by inducing expression of cyclins such as cyclin D and cyclin E [149]. Although direct regulatory links between Akt and CASP14 are not fully understood, the association of CASP14 with highly proliferative, Akt-hyperactive tumors suggests a mechanistic connection. The observed reduction in *CASP14* upon Akt inhibition in our study supports an hypothesis that sustained Akt signaling is required to maintain the pro-tumorigenic CASP14 phenotype.

A number of genes were upregulated in response to combination treatments in SUM149 cells. We found that *TSC22D3*, also known as GILZ (Glucocorticoid-Induced Leucine Zipper) [150, 151], was upregulated by the BMS-536924 + tyrphostin and BMS-536924 + AKTIV combinations (Fig 10). Blocking IGF-1R/EGFR and AKT for 24 h with these drug combinations would reduce Akt activity (and downstream mTOR signaling). A major consequence of PI3K/AKT pathway inhibition is activation of the FOXO family of transcription factors, which translocate to the nucleus and initiate transcription of target genes [152]. We suggest that cells respond to this sudden loss of growth signals by activating alternative transcriptional programs via FOXO and NF-κB, which can upregulate genes involved in stress adaptation, inflammatory feedback, and differentiation. *TSC22D3* (GILZ) is a FOXO-responsive gene that is suggested to be connected to AKT/PI3K suppression [150]. Other reports have also implicated a direct relation between GILZ and Akt, whereby GILZ promotes apoptosis by inhibition of the mTORC2/AKT pathway [153, 154]. PI3K/AKT inhibition (achieved here indirectly through IGF-1R inhibition) has also been shown to induce apoptosis of multiple myeloma cells through increased GILZ expression [154, 155], suggesting a feedback loop mechanism between GILZ and Akt.

Taken together, our RNA Seq analysis showed that the combination treatments suppressed the molecular machinery driving IBC progression, highlighted by significant downregulation of cell cycle regulators, DNA replication enzymes, and survival factors across all treatment groups, indicating robust growth arrest, resulting in the phenotypic effects on reduced motility and 3D spheroid growth that we observed with the drug combinations.

Although we profiled single-agent activity across multiple breast cancer cell lines, the comprehensive assessment and testing of drug combinations were performed primarily in SUM149, as it is a triple-negative/basal-like IBC model (ER/PR negative, HER2 not activated) [54, 156] that represents the most aggressive forms of IBC. This focus may limit the generalizability of our results as IBC is heterogeneous across receptor subtypes [140] and patient tumors; and thus, at this stage, our results should be interpreted as defining synergistic interactions and pathways in SUM149 rather than more broadly across all IBC subtypes. Nonetheless, we suggest that our findings support as proof-of-concept, that co-targeting the IGF-1R/EGFR/PI3K-Akt-mTOR signaling axis can yield strong anti-proliferative and anti-motility effects in the SUM149 TN-IBC model, and future work will test the prioritized combinations (or clinically tractable analogs) in additional IBC models (e.g., HER2 + SUM190 and MDA-IBC-3), as molecular subtype differences may influence the degree to which co-targeting EGFR/IGF-1R and downstream PI3K/Akt/mTOR signaling produces synergy. These combinations can also be assessed in patient-derived xenografts and/or primary patient material to more fully assess translational potential in models that better preserve IBC tissue architecture, tumor emboli, and microenvironmental ligand signaling. While our 3D spheroid results serve as an intermediate level of complexity, they do not capture stromal/immune contributions or systemic exposure constraints.

While we did not directly test our selected drug combinations in other IBC subtypes, we can propose how they might act. HER2 + disease represents a substantial subset of IBC and these tumors frequently signal through PI3K/AKT/mTOR pathways that overlap with the targets of our prioritized combinations [157]. HER2 + IBC biopsied at progression on HER2-targeted therapy reported mTOR activation in 83% of cases, and also noted frequent co-activation of HER2 and mTOR signaling, consistent with PI3K/AKT/mTOR functioning as a bypass/resistance axis [158]. Hence, we anticipate that the downstream co-targeting combinations we identified (e.g., Akt + mTOR inhibition, or IGF-1R/IR + Akt inhibition) may remain relevant in HER2 + IBC, particularly where PI3K pathway activation is present. However, efficacy is expected to be context-dependent (e.g., EGFR dependence and HER-family expression) [159], and translation to HER2 + disease will likely require further investigation. IGF-1R can crosstalk with HER2 signaling; in trastuzumab-resistant HER2 + breast cancer models, co-targeting IGF-1R and HER2 reduced invasion and produced modest but significant growth effects, supporting IGF-1R/IR inhibition as a rational partner node when HER2 signaling is persistent [159]. These data suggest that our downstream-node combinations (e.g., Akt + mTOR or IGF-1R/IR + Akt) may be likely to retain activity in HER2 + IBC models, whereas the IGF-1R/IR + EGFR pair may be more context-dependent (e.g., influenced by EGFR/HER-family expression and compensatory HER2 signaling). In the HER2 + IBC cell line SUM190, mTORC1 inhibitors (rapamycin/everolimus) show only modest single-agent growth inhibition, supporting the need for combination strategies rather than mTOR blockade alone [158]. In the same SUM190 IBC model, the allosteric Akt inhibitor MK-2206 (1 µM) reduced Akt phosphorylation [160], while in another study, the IGF-axis–targeting compound KW-2450 inhibited growth with an IC₅₀ of 0.22 µM [161].

From a translational perspective, two compounds from the top GRR combinations (tyrphostin AG‑1478 and AKTIV) are widely used laboratory inhibitors and should be viewed primarily as chemical probes that help validate pathway co-targeting strategies, rather than as immediate clinical combination partners. Tyrphostin AG‑1478 has been described as discontinued from clinical development and used mainly as an experimental EGFR inhibitor, with preclinical studies reporting systemic effects (e.g., hypomagnesemia and cardiac dysfunction) with chronic exposure, underscoring the need for careful safety assessment when translating EGFR-pathway inhibition [162–164]. AKTIV also has limited clinical development information and as mentioned previously, is reported to accumulate in mitochondria and disrupt cellular bioenergetics, raising the possibility of off-target effects [92]. For both compounds, the established human pharmacokinetic and safety data are limited, which further constrains direct translational interpretation of these compounds. BMS-536924 is an orally active preclinical IGF-1R/IR inhibitor [68], but the broader class of dual IGF-1R/IR kinase inhibitors has been difficult to translate clinically because insulin receptor co-inhibition can produce metabolic toxicity, and later agents such as linsitinib [165] and BMS-754807 [166] did not advance successfully in breast cancer/solid tumor development. For our identified combinations, other inhibitors for the same targets are clinically advanced, including FDA-approved Akt inhibitor capivasertib [114], and approved mTOR inhibitor temsirolimus [167]. Future studies should therefore evaluate whether the synergy observed here can be reproduced with clinically tractable pathway inhibitors, while accounting for pharmacokinetic compatibility and overlapping mechanism-based toxicities common to EGFR and PI3K/Akt/mTOR axis inhibition [168].

An additional important translational consideration is whether the combination effects we observed *in vitro* can be achieved at clinically relevant drug exposures. Approved mTOR and Akt inhibitors achieve clinically relevant exposures at standard doses: temsirolimus (25 mg i.v. weekly) reaches a mean C_max_ of ~585 ng/mL (~sub-µM) in whole blood, and capivasertib, recently approved for HR + /HER2 − advanced breast cancer, achieves steady-state C_max_ values in the low-µM range (1,371 ng/mL (30%)) [113, 169]. Safety is also important for translating PI3K/Akt/mTOR-targeted combinations. Capivasertib was mainly associated with diarrhea and rash [117, 169], while everolimus was associated with stomatitis and other toxicities in BOLERO-2 [90]. Clinical studies of PI3Kα inhibitors such as alpelisib and inavolisib reported pathway-related adverse effects, including hyperglycemia, rash, diarrhea, and/or stomatitis [115, 169]. These findings highlight the need to optimize dose, schedule, and drug selection in any future IBC studies, especially when combining agents that may have overlapping toxicities. Future studies will employ clinically relevant agents (e.g., capivasertib, ipatasertib, and approved EGFR/HER-family inhibitors) and evaluate efficacy and tolerability in *in vivo* IBC and patient-derived models.

Together, our results highlight the therapeutic promise of the identified combinations for targeting IBC, and, to our knowledge, these specific combinations have not been reported previously in IBC. Because the three most effective combinations all came from the GRR-derived list, these findings also support the use of complementary computational approaches to identify effective novel combinations.

While these initial findings are promising, it should be noted that all efficacy data were generated *in vitro* using cell line models, and our findings should currently be considered only as proof-of-concept observations. Additionally, these combinations first need to be validated across additional IBC cell lines. Further, prior to any consideration of clinical translation, combinations would need to be tested in animal models to assess *in vivo* efficacy, pharmacokinetics, and toxicity.

## Conclusions

This study provides experimental in vitro validation of our computational LWAS- and GRR-derived drug repurposing predictions for a rare form of breast cancer, IBC. Among the LWAS-predicted drugs, we identified several agents with known relevance to breast cancer and IBC. For the GRR-predicted drugs, we identified targeted agents and combinations that showed activity in the SUM149 2D and 3D IBC models, with the most active GRR-derived combinations affecting IGF-1R/IR, EGFR, Akt, and mTOR signaling, and partially reversing the IBC-associated gene-expression signature in SUM149 cells. These findings support further testing of clinically tractable inhibitors for these pathways in IBC *in vivo* models and patient-derived systems to more broadly assess efficacy, dosing, resistance mechanisms, and toxicity.

This study bridges the gap between computational predictions and experimental validation in drug repurposing. Our approach of using LWAS-based text mining and gene-reversal analysis to predict potential drugs, then assessing them in lab-based models for efficacy, should be a generally applicable strategy for identifying possible therapies for a range of understudied rare diseases with limited treatment options.

## Supporting information

S1 Fig Representative dose response curves for the LWAS drug candidates with efficacy in breast cancer cell lines using the Hoechst assay. (A) SUM159, (B) MDA-MB-231, and (C) MCF-7. Each graph shows the relative cell count (%) plotted against drug concentration (μM) on a logarithmic scale. Plots are grouped by drug mechanisms: antimetabolites, microtubule inhibitors, topoisomerase inhibitors, and tyrosine kinase inhibitors. Error bars indicate ± SD from replicate experiments.
(TIF)

S2 Fig Representative dose response curves of the GRR effective compounds with efficacy in different breast cancer cell lines using the Hoechst assay. (A) SUM159, (B) MDA-MB-231 and (C) MCF-7s. The x-axis represents the concentration of the compound in µM while the y-axis represents the cell count % normalized to DMSO control. All the experiments were done in technical and independent experiments (n = 3). Error bars indicate ± SD from replicate experiments.
(TIF)

S3 Fig Drug sensitivity heatmap profiles and dose response curves for LWAS drugs in IBC and non-IBC breast cancer cell line panel by MTT assay. (A) Drug response heatmaps across all the breast cancer cell lines. The x-axis represents the concentration of each drug delivered in a dose-response, while the y-axis lists the compounds. The color gradient indicates the level of inhibition, with red indicating higher inhibition and green indicating lower inhibition. Representative dose response curves grouped by drug class: antimetabolites, microtubule inhibitors, topoisomerase inhibitors, and TKIs, for (B) SUM149, (C) SUM159, (D) MDA-MB-231 and (E) MCF-7. Inhibition data plotted as the percentage (%) of cell proliferation relative to control versus drug concentration (µM). Error bars indicate ± SD from replicate experiments.
(PDF)

S4 Fig Drug sensitivity heatmap profiles and dose response curves for GRR drugs in IBC and non-IBC breast cancer cell line panel by MTT assay. (A) Drug response heatmaps across all the breast cancer cell lines. The x-axis represents the concentration of each drug delivered in a dose-response, while the y-axis lists the compounds. The color gradient indicates the level of inhibition, with red indicating higher inhibition and green indicating lower inhibition. Representative dose response curves for each drug: BMS-536924, BMS-754807, Akt Inhibitor IV, temsirolimus, tipifarnib, and tyrphostin AG 1478 in the four breast cancer cell lines with (B) SUM149, (C) SUM159, (D) MDA-MB-231 and (E) MCF-7. Inhibition data plotted as the percentage (%) of cell proliferation relative to control versus drug concentration (µM). Error bars indicate ± SD from replicate experiments.
(PDF)

S5 Fig Contour Plots of selected LWAS drug combinations with their synergy scores across Bliss, ZIP, Loewe and HSA reference models obtained from SynergyFinder3. Synergy scores across Bliss, ZIP, Loewe and HSA reference models were obtained from SynergyFinder3. (A) Lapatinib + doxorubicin (B) Lapatinib + carboplatin (C) Carboplatin + etoposide (D) Carboplatin + topotecan (E) Vincristine + docetaxel, (F) Gefitinib + carboplatin and (G) Carboplatin + cytarabine. In each plot, x and y axes represent drug concentrations (µM). Red areas indicate synergy, while green areas indicate antagonism. Black boxes highlight the MSA, with scores shown. Red circles in the top right corner of each plot indicate the overall δ score. Color scales on the right of each plot show the range of synergy scores. The data represented the average of three independent experiments (n = 3).
(PDF)

S6 Fig Quantitative synergy analysis of GRR drug combinations in SUM149 2D and 3D SUM149 cell models. Synergy scores were computed using SynergyFinder+ across four established reference models (ZIP, HSA, Bliss, and Loewe). Results are shown as bar plots for each combination. (A) BMS-536924 and Tyrphostin AG-1478 combination, (B) BMS-536924 and Akt Inhibitor IV combination, and (C) Temsirolimus and Akt Inhibitor IV combination. The upper figure for each combination represents the results from the 2D experiments, while the lower figure represents the results from the 3D experiments. Bar plots represent the drug combination landscape, where red bars indicate positive synergy scores, and green bars indicate antagonistic effects. Blue bars highlight the optimal combinations achieving maximal synergy scores and growth inhibition while minimizing individual drug concentration, which were: (1 µM BMS-536924 + 0.1 µM tyrphostin AG-1478), (1 µM BMS-536924 + 1 µM Akt Inhibitor IV), and (10 µM temsirolimus + 0.1 µM Akt Inhibitor IV). The data shown represents the average of three independent experiments.
(PDF)

S7 Fig Western blot analysis of protein expression levels in response to the selected GRR drugs and combinations. Representative whole-blot western blots for replicate DMSO and combination incubations with SUM149 cells. (A) Temsirolimus (10 µM) and AKTIV (0.1 µM) combination effects on the expression of p-Akt, total Akt, p-mTOR, and total mTOR; (B) Akt Inhibitor IV (1 µM) and BMS-536924 (1 µM) combination effects on the expression of p-Akt, total Akt, p-IGF-1R, and total IGF-1R; and (C) Tyrphostin AG-1478 (0.1 µM) and BMS-536924 (1 µM) combination effects on the expression of p-EGFR, total EGFR, p-IGF-1R, and total IGF-1R. β-actin served as a loading control. Right panels show densitometric band quantification of DMSO relative to each treatment. The Li-COR system was used for all proteins except for IGF-1R and p-IGF-1R, for which we used HRP-tagged secondary antibodies and iBRIGHT imaging. Statistical significance set as *P < 0.05, **P < 0.01, ***P < 0.001. All blots shown in S7 are original uncropped and unadjusted images.
(TIF)

S8 Fig Global gene expression changes induced by GRR combinations in SUM149 cells. SUM149 cells were incubated with GRR combinations or DMSO alone for 24 h, and RNA isolated for RNA-Seq (n = 3 biological replicates). RNA-Seq data were analyzed to identify genes significantly differentially expressed in SUM149 cells (padj < 0.05). (A) Differential gene expression (DGE) analysis. The number of genes differentially expressed in the combination treatment relative to the DMSO control. Total (black columns), up-regulated (red columns) and down-regulated (blue columns). (B) Heat maps show normalized expression values (log scale; blue = lower, red = higher) for the top 25 up‑regulated and top 25 down‑regulated protein-coding genes across biological triplicates. (i) to (iii) Treatment comparison: (i) DMSO (0.2%) vs BMS‑536924 (1 μM) + tyrphostin AG1478 (0.1 μM) (BMS24-Tyrpho). (ii) DMSO (0.2%) vs BMS‑536924 (1 μM) + AKTIV (1 μM) (BMS24-Akt). (iii) DMSO (0.2%) vs AKTIV (0.1 μM) + temsirolimus (10 μM) (Tem-Akt).
(TIF)

S9 Fig Raw images for all Western blots. This file includes the unaltered, uncropped blot images underlying all western blot images in Figures 9 and S7. Western blot experiments and samples are detailed in the Figure 9 legend. Each whole blot shows replicate experiments. The samples used for the figure panels in Figure 9 are indicated by the black rectangles. All blots shown in S7 Fig are the whole blots, and are also included in this file.
(PDF)

S1 Table The 24 LWAS identified drugs for IBC (from Ji et al., 2020). The table presents the corresponding drug class, PubMed entry, Clinical Trials entry, and NCT number.
(DOCX)

S2 Table The 19 GRR identified drugs/compounds (from Ji et al., 2023). The table shows with the corresponding drug class for each drug and its mechanism of action.
(DOCX)

S3 Table Efficacy of LWAS and GRR predicted drugs and compounds in four breast cancer cell lines using the MTT assay. The table lists the IC_50_ values for each drug determined in the MTT assay across the cell lines, with values reported as the mean ± SD from 2 independent experiments.
(DOCX)

S4 Table Selected drugs and compounds from LWAS and GRR active lists for combination studies. The table shows their corresponding EC_25_ values and the highest concentration used for the 8 x 8 matrix.
(DOCX)
