# Supplementary material for: From in silico prediction to experimental validation: Identification of drugs and novel synergistic combinations that inhibit growth of inflammatory breast cancer cells
Source: PLoS One. 2026 Sep 16;21(9):e0339543. doi: 10.1371/journal.pone.0339543 (PMC13581016; doi:10.1371/journal.pone.0339543)
Supplement: Supplementary file 1 [file pone.0339543.s003.pdf]

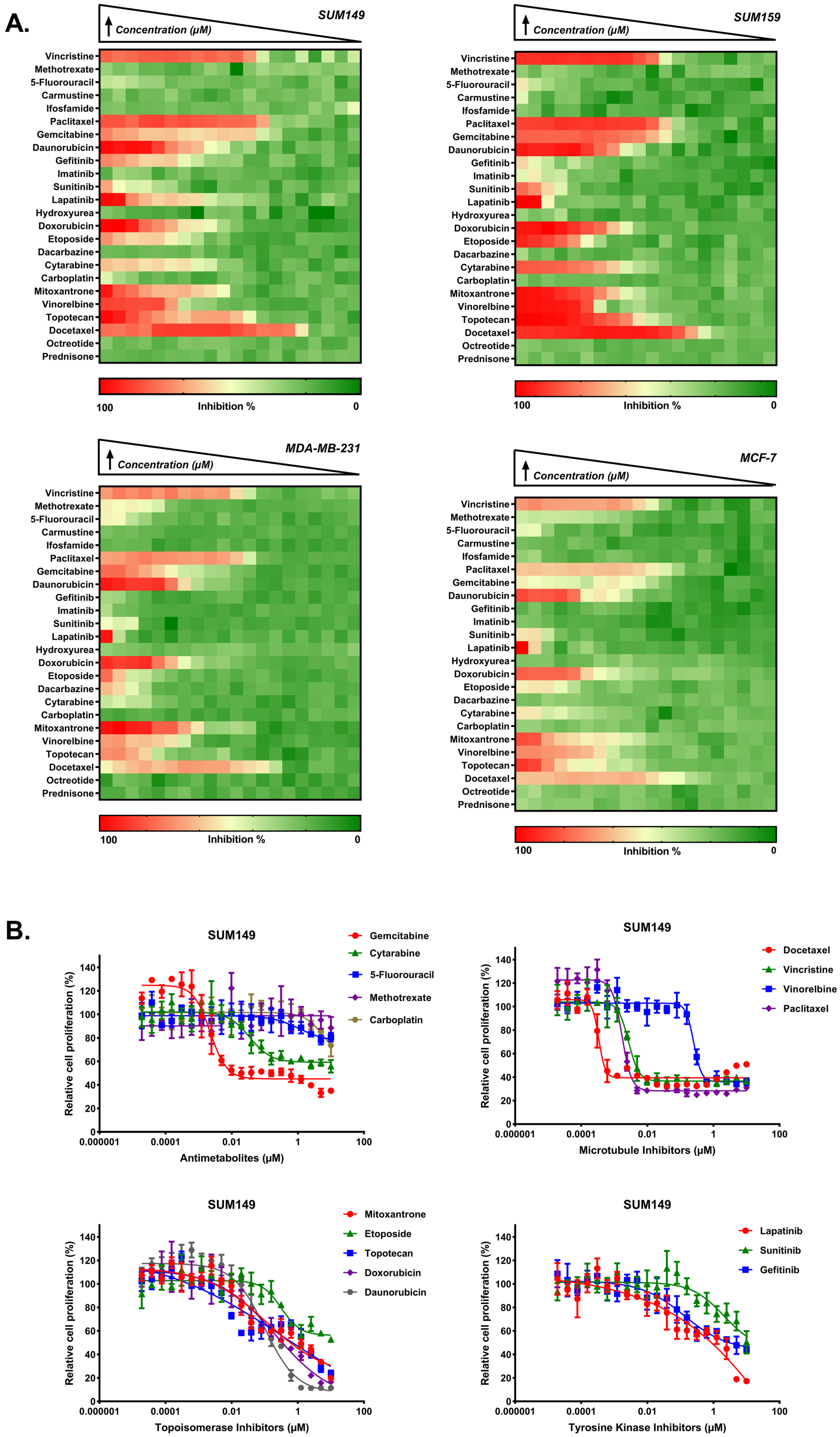
A.
SUM149
Concentration (μM)
Vincristine
Methotrexate
5-Fluorouracil
Carmustine
Ifosfamide
Paclitaxel
Gemcitabine
Daunorubicin
Gefitinib
Imatinib
Sunitinib
Lapatinib
Hydroxyurea
Doxorubicin
Etoposide
Dacarbazine
Cytarabine
Carboplatin
Mitoxantrone
Vinorelbine
Topotecan
Docetaxel
Octreotide
Prednisone
100
Inhibition %
0
SUM159
Concentration (μM)
100
Inhibition %
0
MDA-MB-231
Concentration (μM)
100
Inhibition %
0
MCF-7
Concentration (μM)
100
Inhibition %
0
B.
SUM149
Gemcitabine
Cytarabine
5-Fluorouracil
Methotrexate
Carboplatin
Relative cell proliferation (%)
Antimetabolites (μM)
SUM149
Docetaxel
Vincristine
Vinorelbine
Paclitaxel
Relative cell proliferation (%)
Microtubule Inhibitors (μM)
SUM149
Mitoxantrone
Etoposide
Topotecan
Doxorubicin
Daunorubicin
Relative cell proliferation (%)
Topoisomerase Inhibitors (μM)
SUM149
Lapatinib
Sunitinib
Gefitinib
Relative cell proliferation (%)
Tyrosine Kinase Inhibitors (μM)
0.000001
0.0001
0.01
1
100

C.

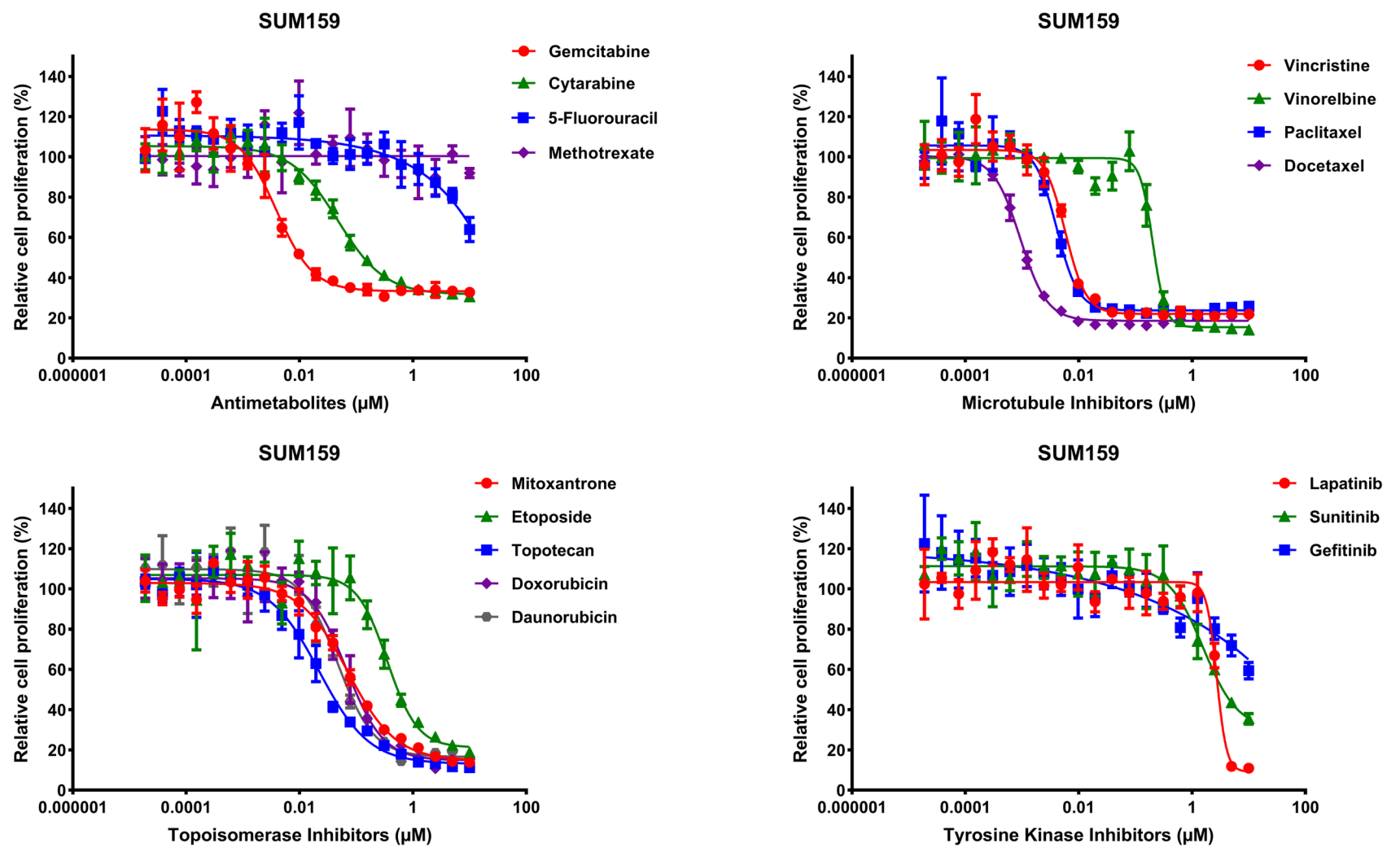
SUM159
Gemcitabine
Cytarabine
5-Fluorouracil
Methotrexate
Relative cell proliferation (%)
Antimetabolites (µM)
SUM159
Vincristine
Vinorelbine
Paclitaxel
Docetaxel
Relative cell proliferation (%)
Microtubule Inhibitors (µM)
SUM159
Mitoxantrone
Etoposide
Topotecan
Doxorubicin
Daunorubicin
Relative cell proliferation (%)
Topoisomerase Inhibitors (µM)
SUM159
Lapatinib
Sunitinib
Gefitinib
Relative cell proliferation (%)
Tyrosine Kinase Inhibitors (µM)
0.000001
0.0001
0.01
1
100

D.

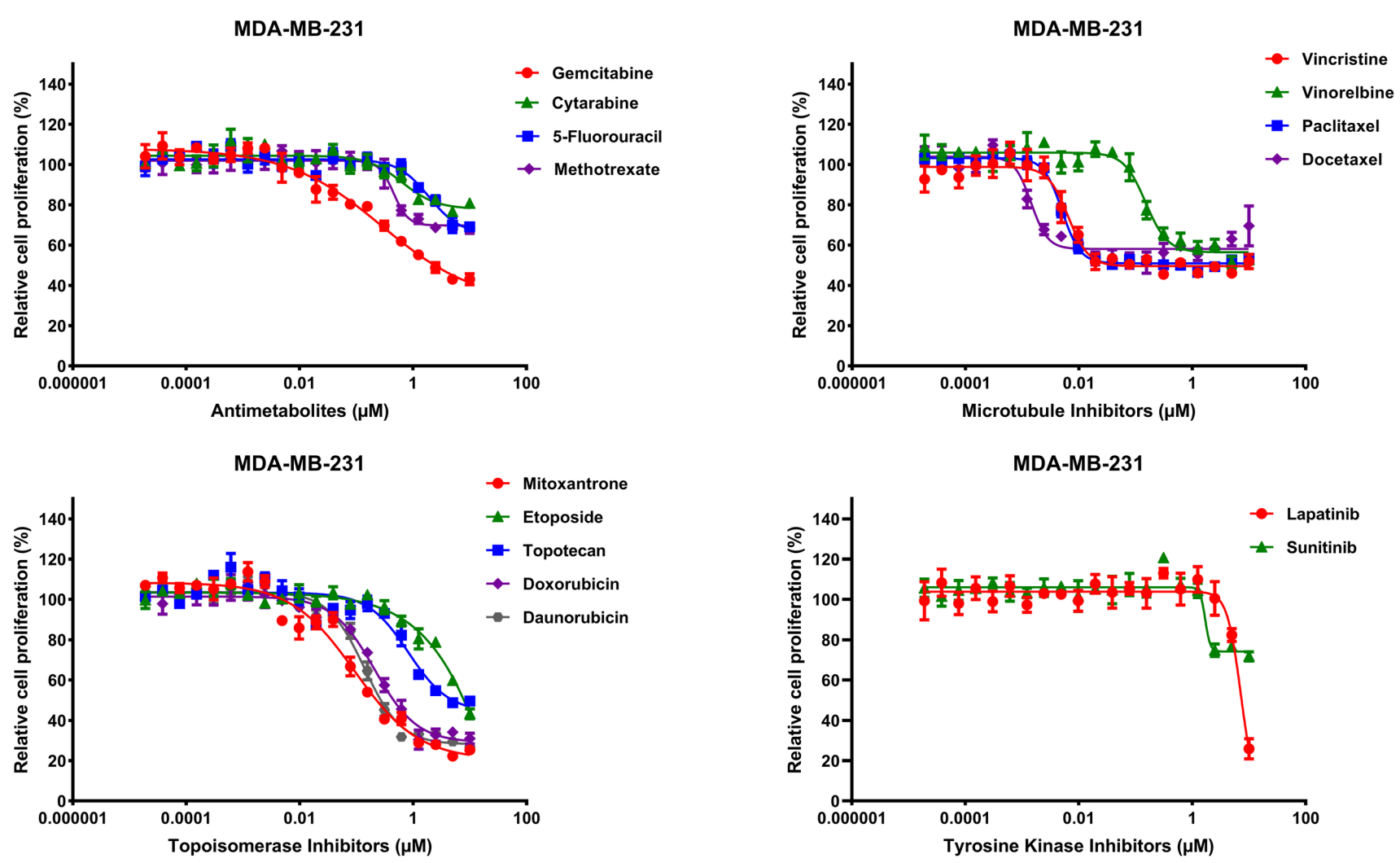
MDA-MB-231
Gemcitabine
Cytarabine
5-Fluorouracil
Methotrexate
Relative cell proliferation (%)
Antimetabolites (µM)
MDA-MB-231
Vincristine
Vinorelbine
Paclitaxel
Docetaxel
Relative cell proliferation (%)
Microtubule Inhibitors (µM)
MDA-MB-231
Mitoxantrone
Etoposide
Topotecan
Doxorubicin
Daunorubicin
Relative cell proliferation (%)
Topoisomerase Inhibitors (µM)
MDA-MB-231
Lapatinib
Sunitinib
Relative cell proliferation (%)
Tyrosine Kinase Inhibitors (µM)

E.

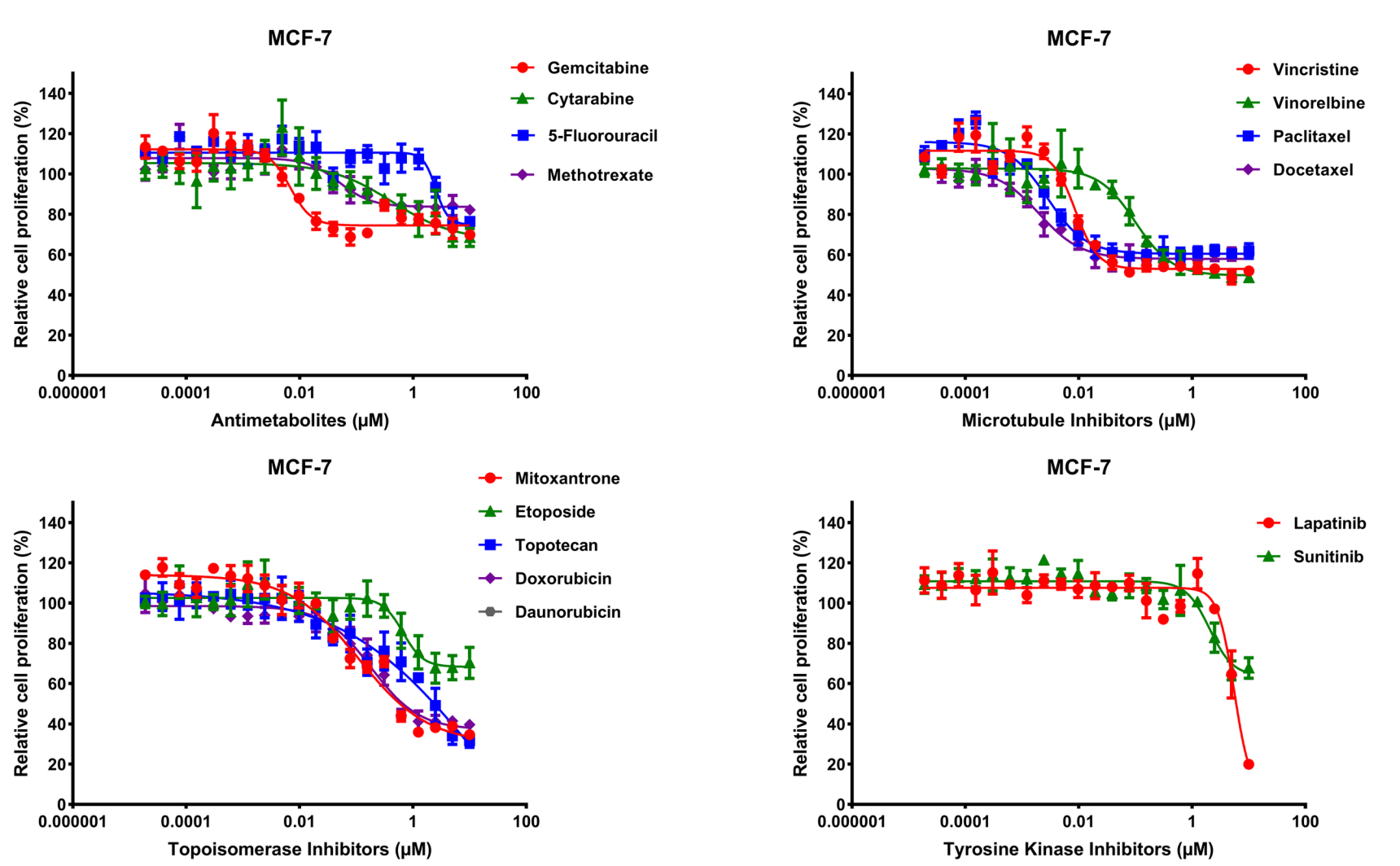
MCF-7
Gemcitabine
Cytarabine
5-Fluorouracil
Methotrexate
Relative cell proliferation (%)
Antimetabolites (µM)
MCF-7
Vincristine
Vinorelbine
Paclitaxel
Docetaxel
Relative cell proliferation (%)
Microtubule Inhibitors (µM)
MCF-7
Mitoxantrone
Etoposide
Topotecan
Doxorubicin
Daunorubicin
Relative cell proliferation (%)
Topoisomerase Inhibitors (µM)
MCF-7
Lapatinib
Sunitinib
Relative cell proliferation (%)
Tyrosine Kinase Inhibitors (µM)
