# Supplementary material for: From in silico prediction to experimental validation: Identification of drugs and novel synergistic combinations that inhibit growth of inflammatory breast cancer cells
Source: PLoS One. 2026 Sep 16;21(9):e0339543. doi: 10.1371/journal.pone.0339543 (PMC13581016; doi:10.1371/journal.pone.0339543)
Supplement: Supplementary file 2 [file pone.0339543.s004.pdf]

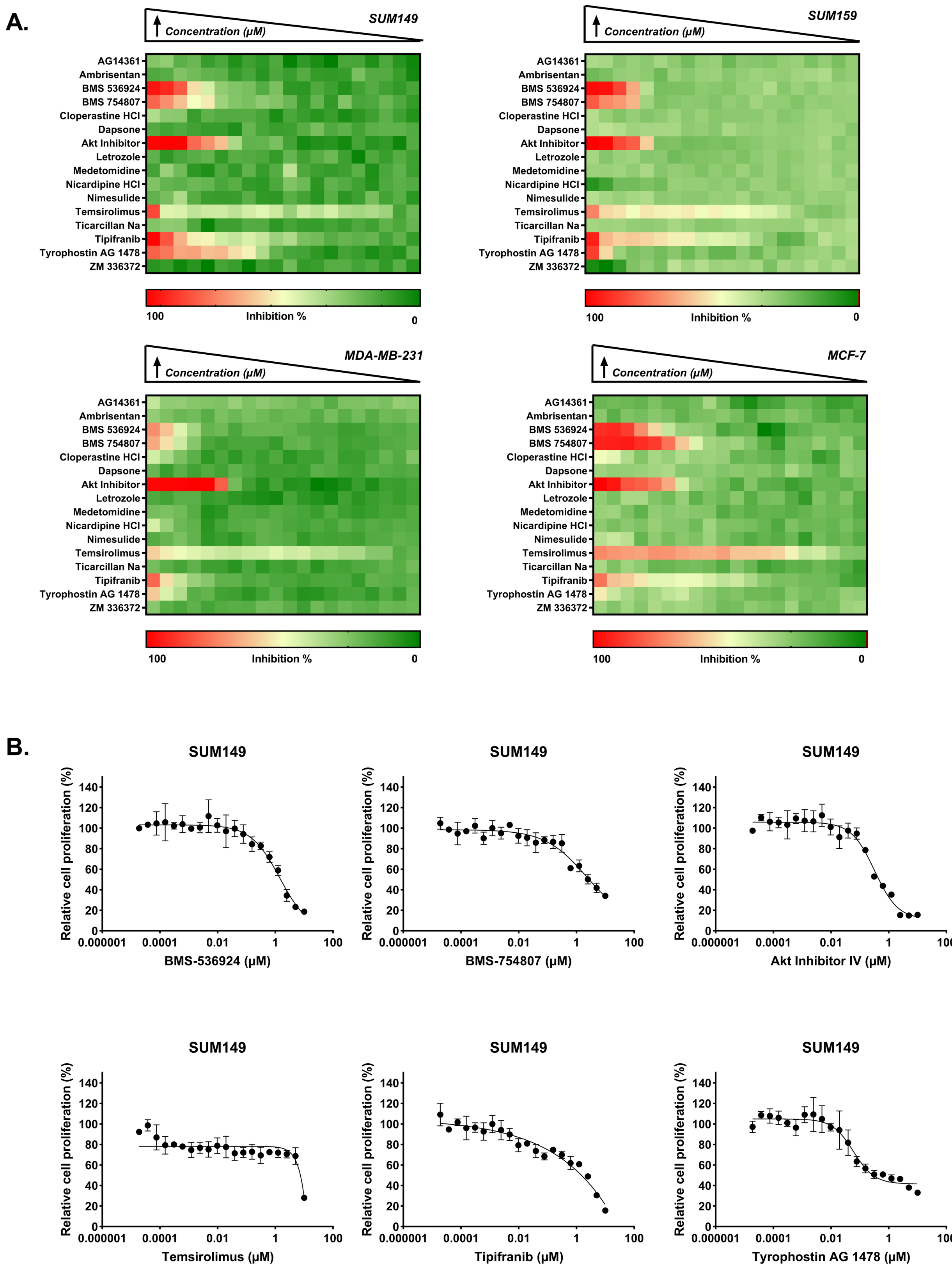
A.
SUM149
SUM159
MDA-MB-231
MCF-7
Concentration (μM)
AG14361
Ambrisentan
BMS 536924
BMS 754807
Cloperastine HCl
Dapsone
Akt Inhibitor
Letrozole
Medetomidine
Nicardipine HCl
Nimesulide
Temsirolimus
Ticarcillan Na
Tipifranib
Tyrophostin AG 1478
ZM 336372
100
Inhibition %
0
B.
SUM149
Relative cell proliferation (%)
BMS-536924 (μM)
BMS-754807 (μM)
Akt Inhibitor IV (μM)
Temsirolimus (μM)
Tipifranib (μM)
Tyrophostin AG 1478 (μM)
0.000001
0.0001
0.01
1
100

C.

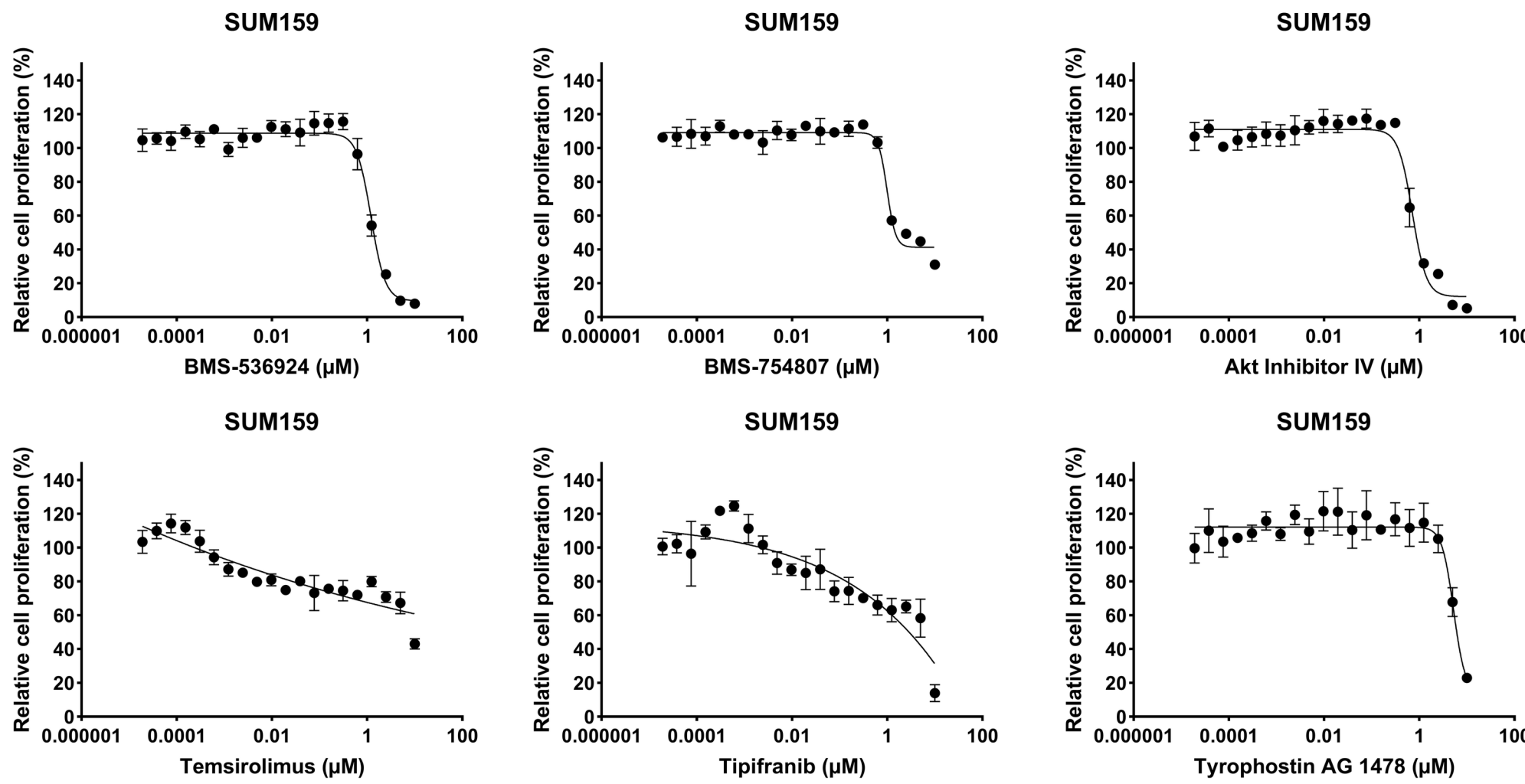
SUM159
Relative cell proliferation (%)
BMS-536924 (μM)
BMS-754807 (μM)
Akt Inhibitor IV (μM)
Temsirolimus (μM)
Tipifranib (μM)
Tyrophostin AG 1478 (μM)

D.

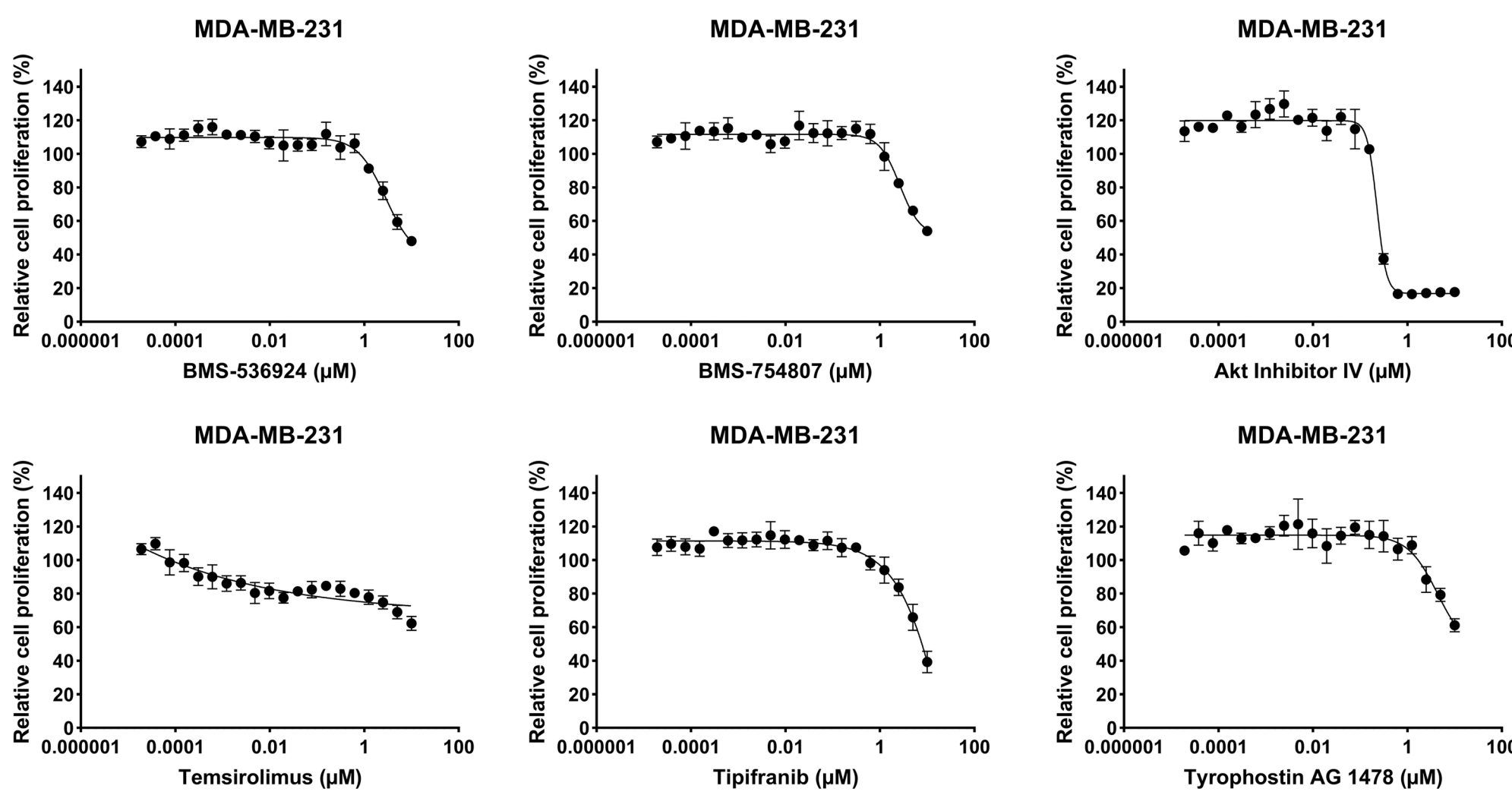
MDA-MB-231
Relative cell proliferation (%)
BMS-536924 (μM)
BMS-754807 (μM)
Akt Inhibitor IV (μM)
Temsirolimus (μM)
Tipifranib (μM)
Tyrophostin AG 1478 (μM)

E.

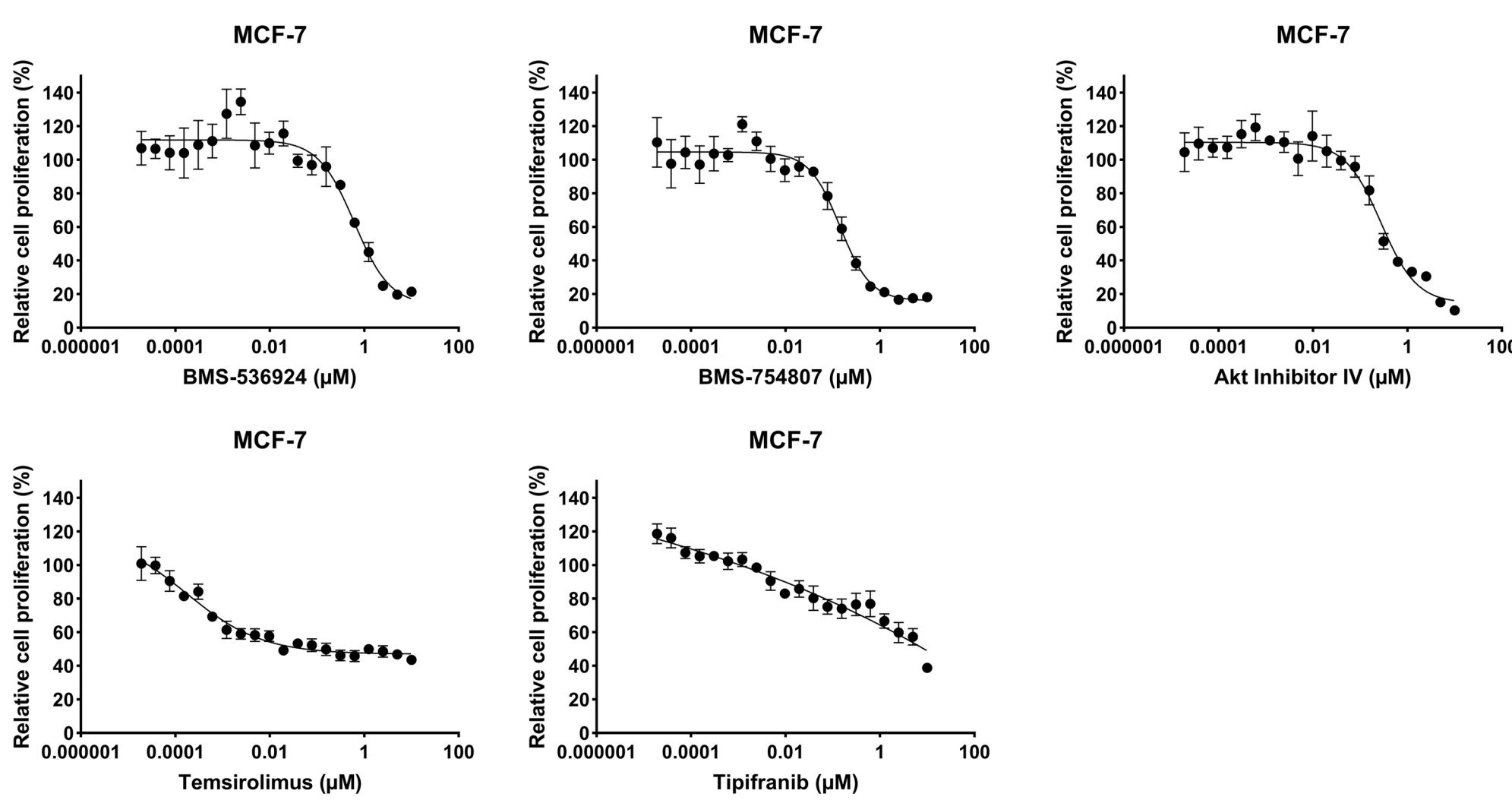
MCF-7
Relative cell proliferation (%)
BMS-536924 (μM)
BMS-754807 (μM)
Akt Inhibitor IV (μM)
Temsirolimus (μM)
Tipifranib (μM)
