# Supplementary material for: From in silico prediction to experimental validation: Identification of drugs and novel synergistic combinations that inhibit growth of inflammatory breast cancer cells
Source: PLoS One. 2026 Sep 16;21(9):e0339543. doi: 10.1371/journal.pone.0339543 (PMC13581016; doi:10.1371/journal.pone.0339543)
Supplement: Supplementary file 3 [file pone.0339543.s005.pdf]

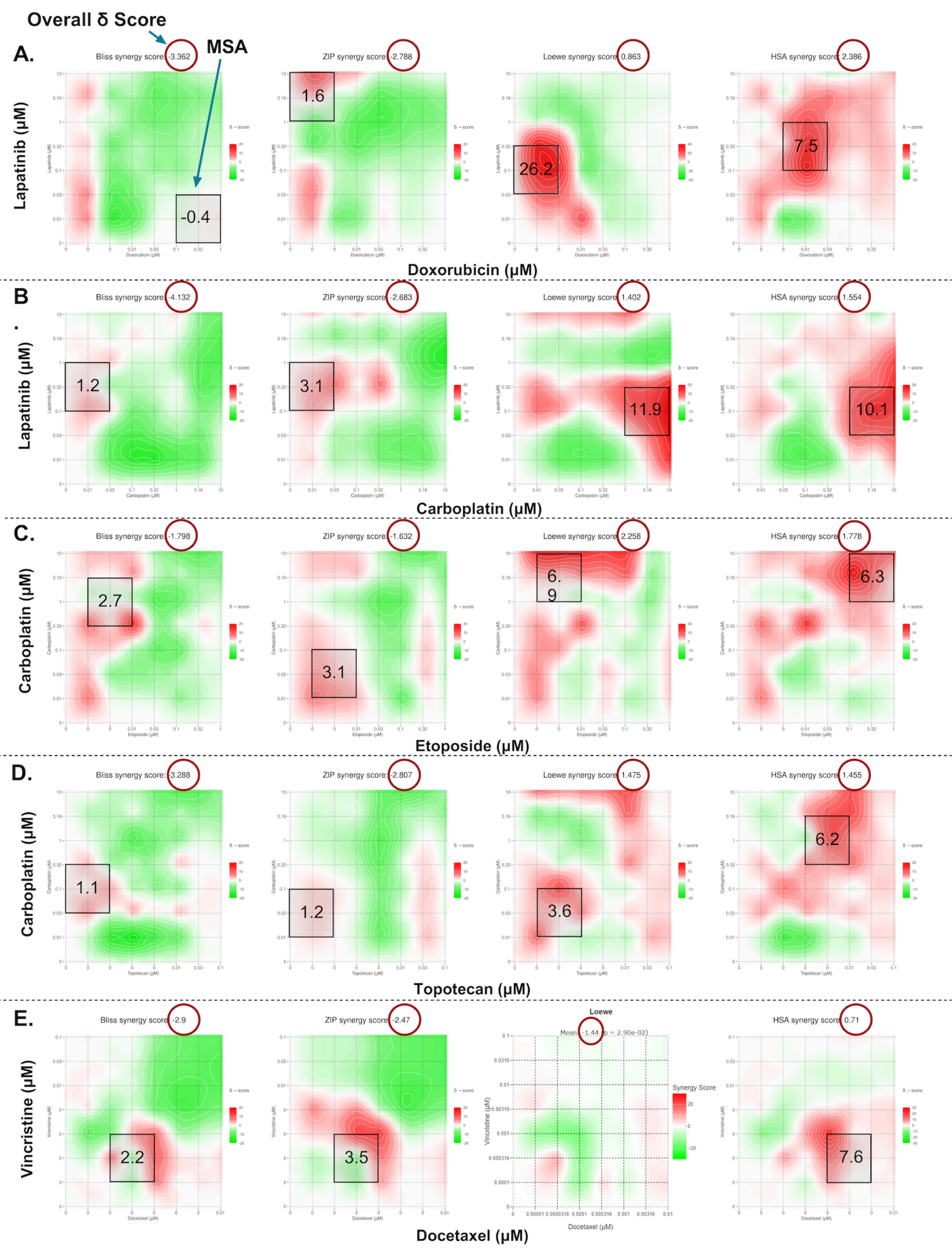

Overall δ Score
MSA
A.
Lapatinib (μM)
Bliss synergy score -3.362
-0.4
ZIP synergy score -2.788
1.6
Loewe synergy score 0.863
26.2
HSA synergy score 2.386
7.5
Doxorubicin (μM)
B
Lapatinib (μM)
Bliss synergy score -4.132
1.2
ZIP synergy score -2.683
3.1
Loewe synergy score 1.402
11.9
HSA synergy score 1.554
10.1
Carboplatin (μM)
C.
Carboplatin (μM)
Bliss synergy score -1.798
2.7
ZIP synergy score -1.632
3.1
Loewe synergy score 2.258
6.
9
HSA synergy score 1.778
6.3
Etoposide (μM)
D.
Carboplatin (μM)
Bliss synergy score -3.288
1.1
ZIP synergy score -2.807
1.2
Loewe synergy score 1.475
3.6
HSA synergy score 1.455
6.2
Topotecan (μM)
E.
Vincristine (μM)
Bliss synergy score -2.9
2.2
ZIP synergy score -2.47
3.5
Loewe
Mean: 1.44 (p = 2.90e-02)
Synergy Score
HSA synergy score 0.71
7.6
Docetaxel (μM)

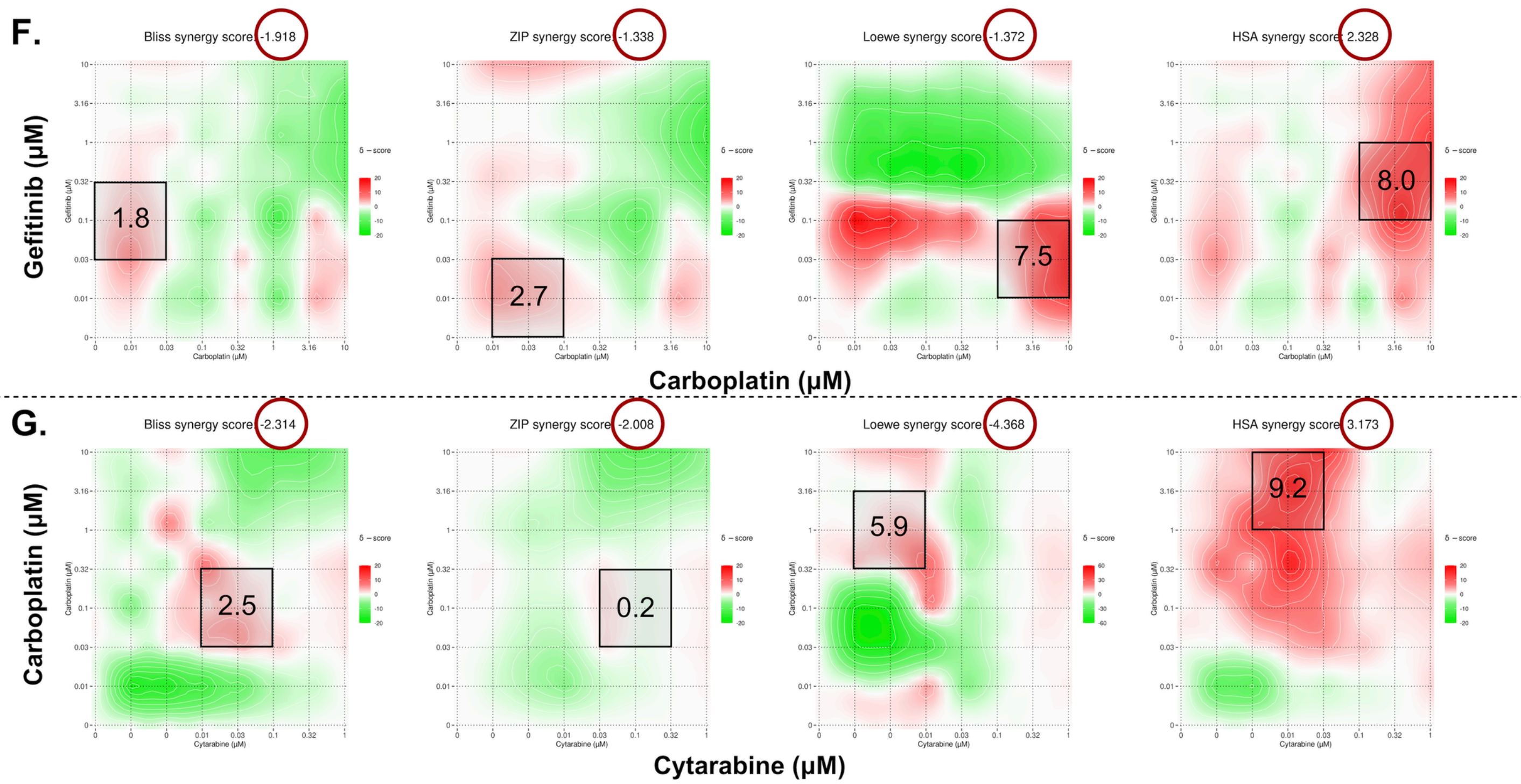
F.
Bliss synergy score: -1.918
ZIP synergy score: -1.338
Loewe synergy score: -1.372
HSA synergy score: 2.328
Gefitinib (μM)
1.8
2.7
7.5
8.0
Carboplatin (μM)
G.
Bliss synergy score: -2.314
ZIP synergy score: -2.008
Loewe synergy score: -4.368
HSA synergy score: 3.173
Carboplatin (μM)
2.5
0.2
5.9
9.2
Cytarabine (μM)
