# Supplementary material for: From in silico prediction to experimental validation: Identification of drugs and novel synergistic combinations that inhibit growth of inflammatory breast cancer cells
Source: PLoS One. 2026 Sep 16;21(9):e0339543. doi: 10.1371/journal.pone.0339543 (PMC13581016; doi:10.1371/journal.pone.0339543)
Supplement: Supplementary file 4 [file pone.0339543.s006.pdf]

A.

2D

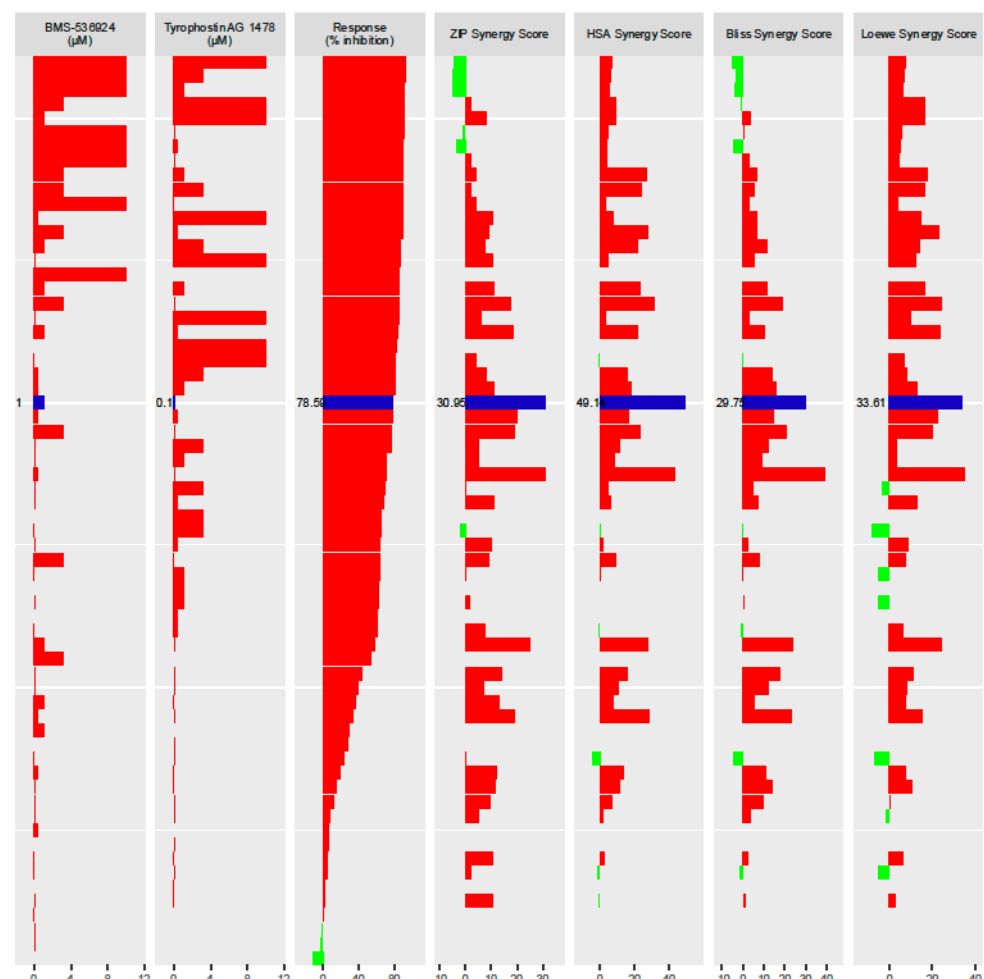
BMS-536924
(μM)
Tyrophostin AG 1478
(μM)
Response
(% inhibition)
ZIP Synergy Score
HSA Synergy Score
Bliss Synergy Score
Loewe Synergy Score
1
0.1
33.61
0 4 8 12
0 4 8 12
0 40 80
-10 0 10 20 30
0 20 40
-10 0 10 20 30 40
0 20 40

3D

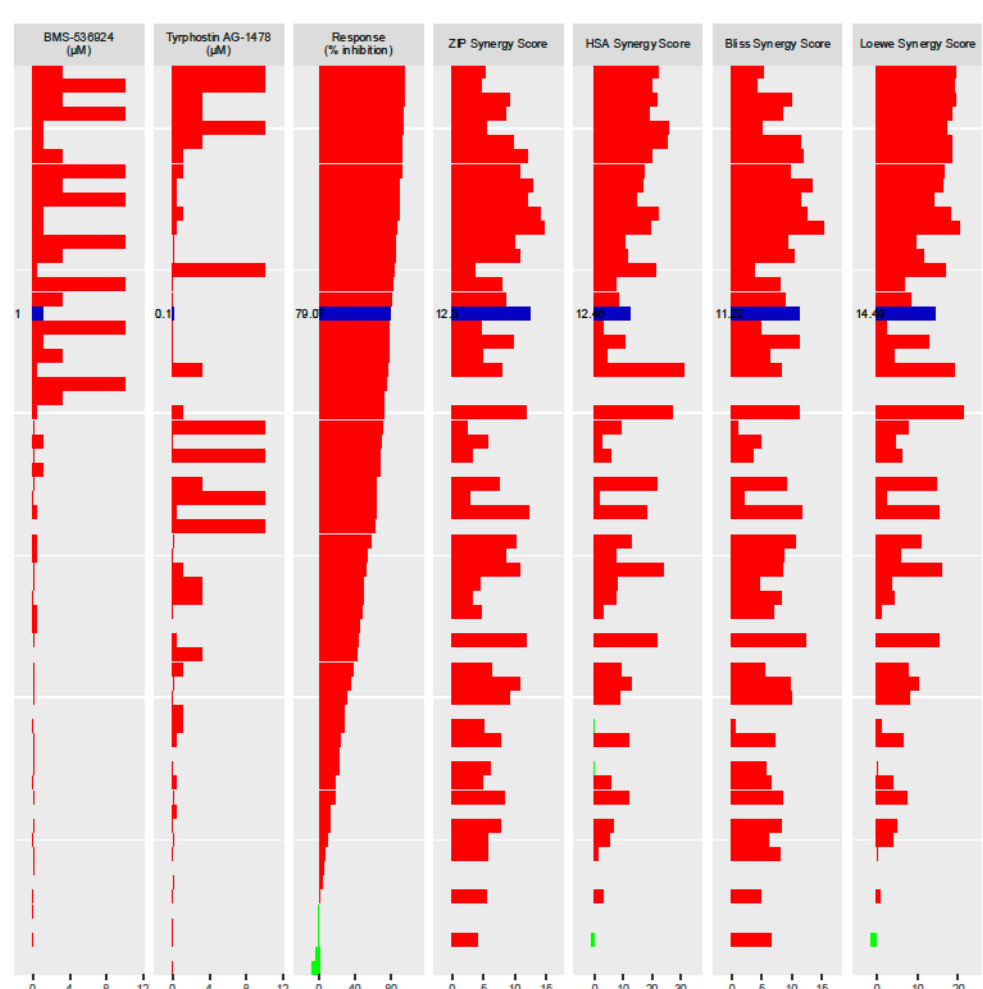
BMS-536924
(μM)
Tyrphostin AG-1478
(μM)
Response
(% inhibition)
ZIP Synergy Score
HSA Synergy Score
Bliss Synergy Score
Loewe Synergy Score
1
0.1
0 4 8 12
0 4 8 12
0 40 80
0 5 10 15
0 10 20 30
0 5 10 15
0 10 20

B.

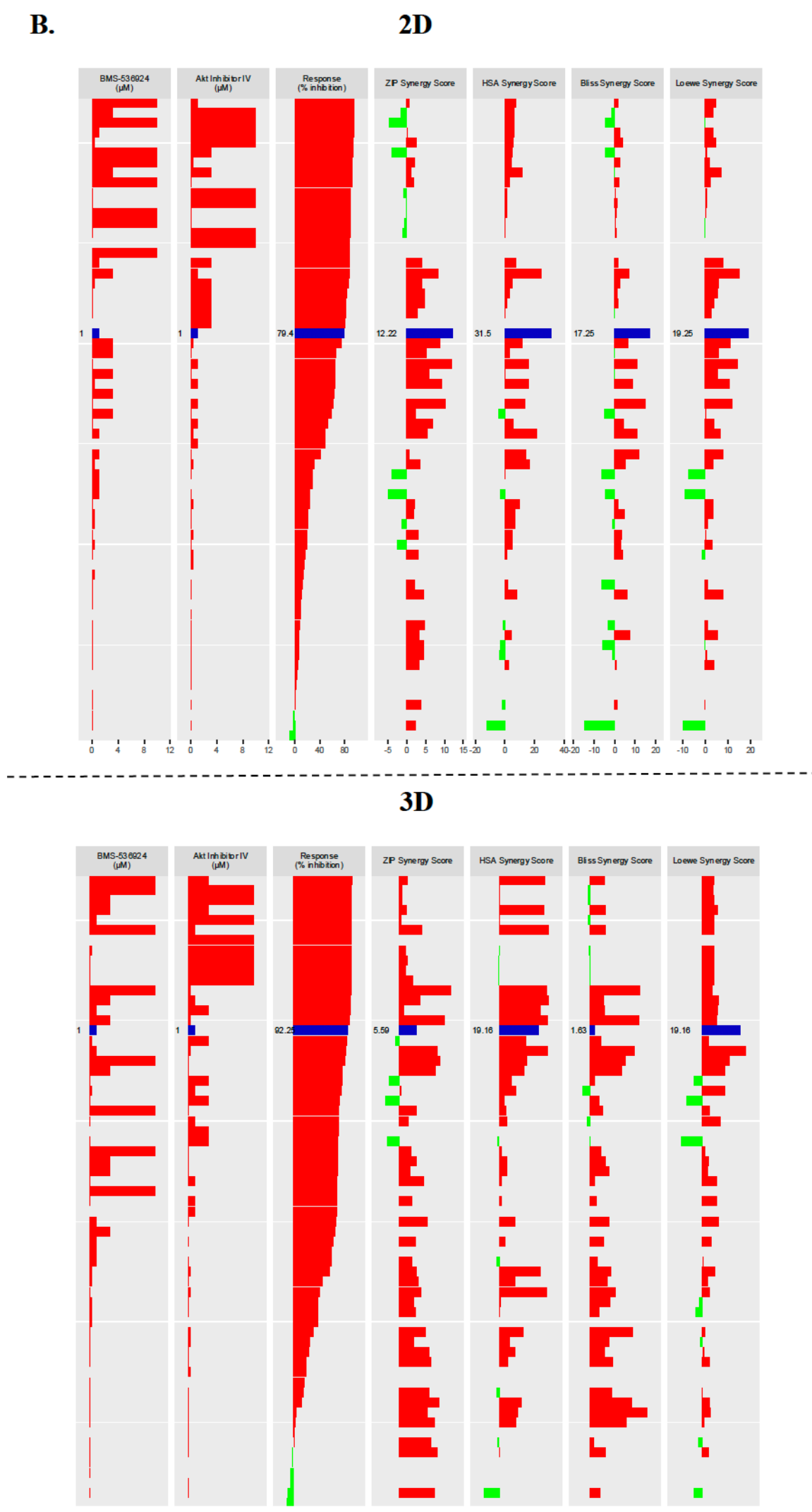
2D
BMS-536924 (μM)
Akt Inhibitor IV (μM)
Response (% inhibition)
ZIP Synergy Score
HSA Synergy Score
Bliss Synergy Score
Loewe Synergy Score
79.4
12.22
31.5
17.25
19.25
3D
BMS-536924 (μM)
Akt Inhibitor IV (μM)
Response (% inhibition)
ZIP Synergy Score
HSA Synergy Score
Bliss Synergy Score
Loewe Synergy Score
92.2
5.59
19.16
1.63
19.16

C.

2D

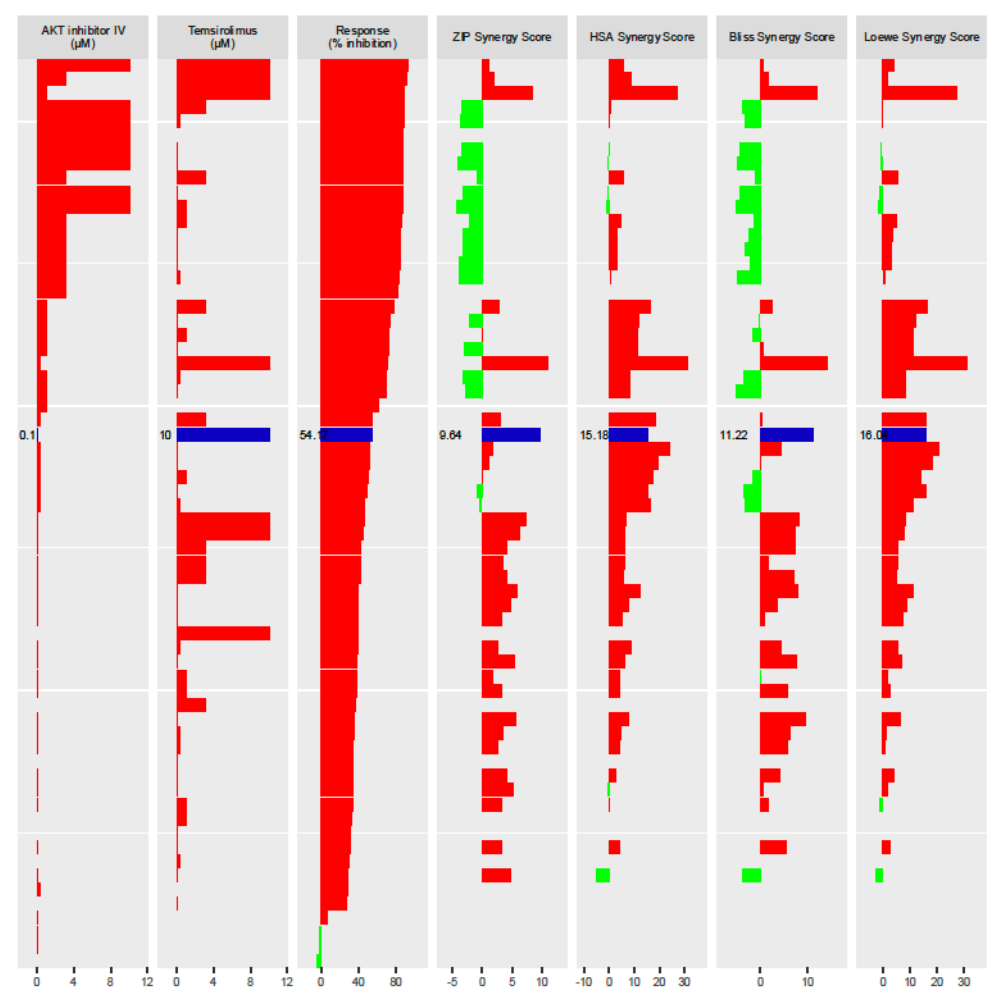

AKT inhibitor IV (μM)
Temsirolimus (μM)
Response (% inhibition)
ZIP Synergy Score
HSA Synergy Score
Bliss Synergy Score
Loewe Synergy Score
0.1
10
9.64
11.22
0 4 8 12
0 4 8 12
0 40 80
-5 0 5 10
-10 0 10 20 30
0 10
0 10 20 30

3D

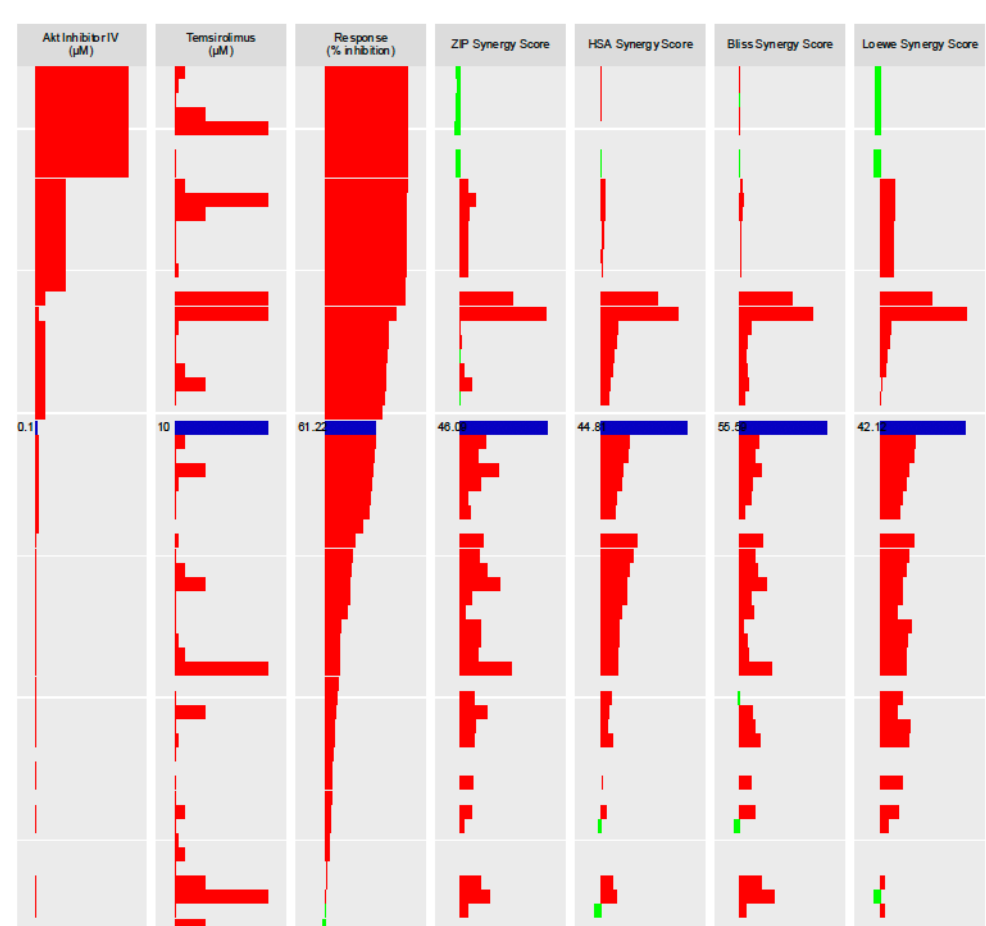

Akt Inhibitor IV (μM)
Temsirolimus (μM)
Response (% inhibition)
ZIP Synergy Score
HSA Synergy Score
Bliss Synergy Score
Loewe Synergy Score
0.1
10
