# Supplementary material for: From in silico prediction to experimental validation: Identification of drugs and novel synergistic combinations that inhibit growth of inflammatory breast cancer cells
Source: PLoS One. 2026 Sep 16;21(9):e0339543. doi: 10.1371/journal.pone.0339543 (PMC13581016; doi:10.1371/journal.pone.0339543)
Supplement: Supplementary file 5 [file pone.0339543.s009.pdf]

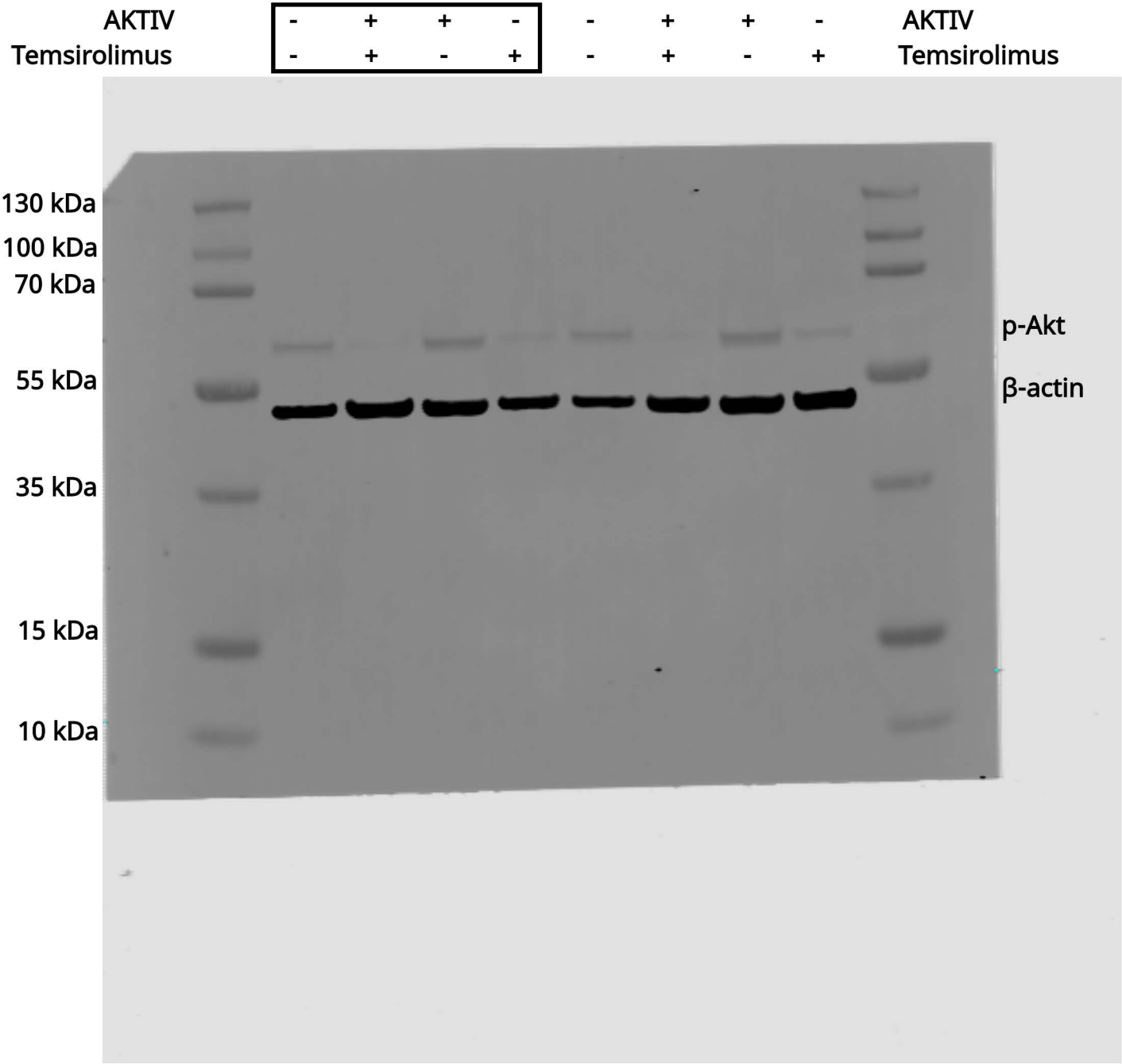

Figure 9A. p-Akt in AKTIV + Temsirolimus

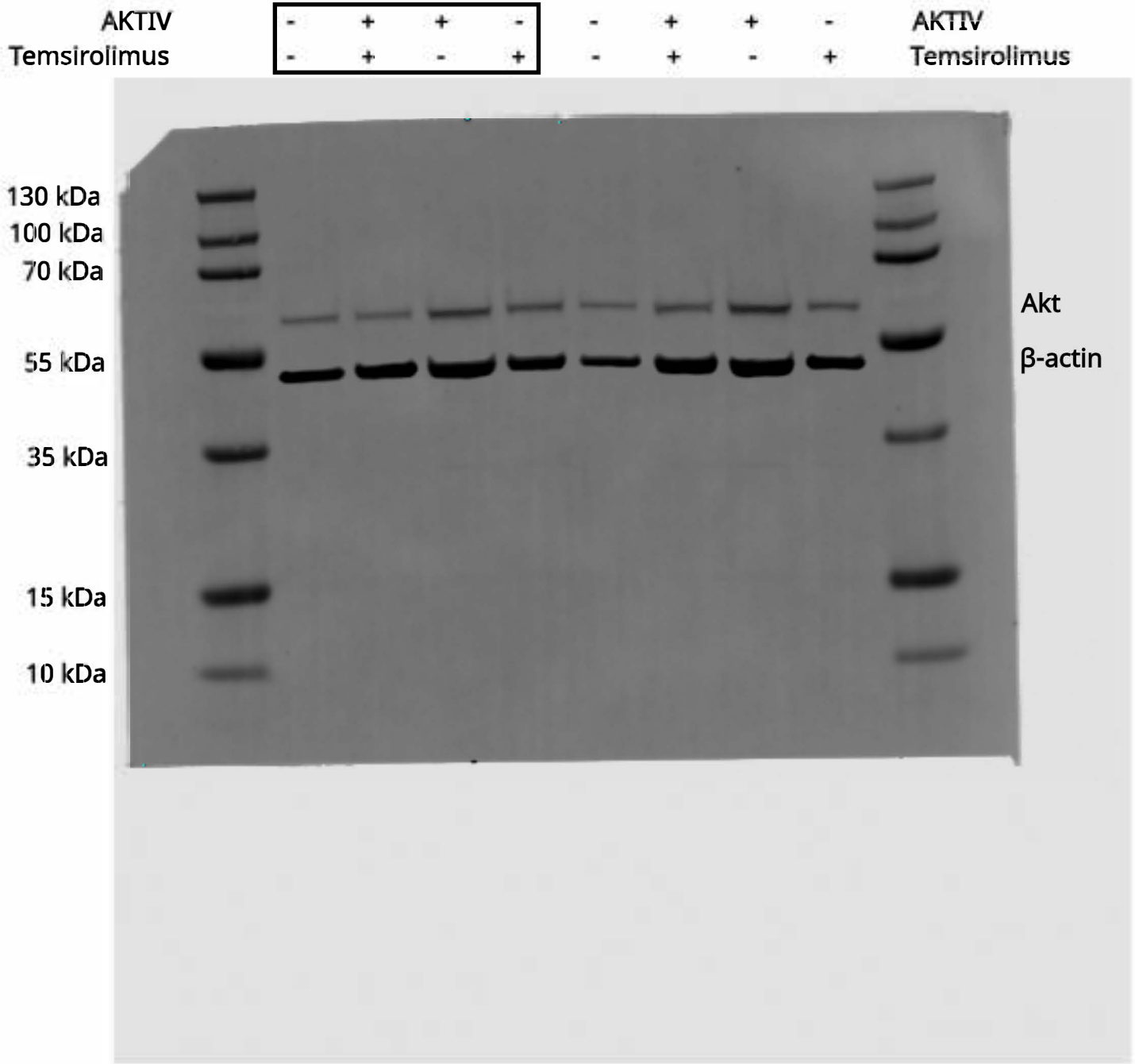

Figure 9A. Akt in AKTIV + Temsirolimus

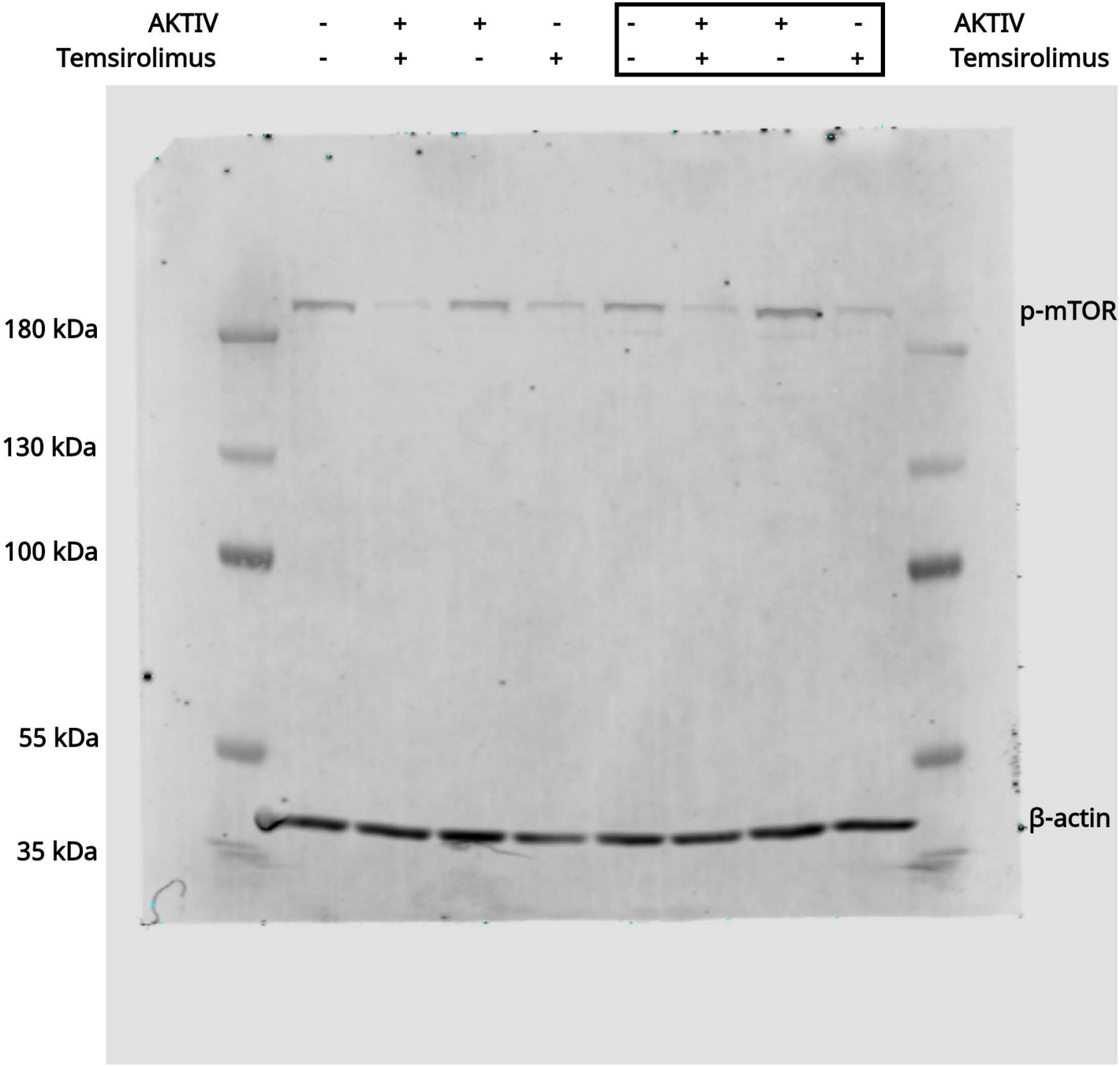

Figure 9A. p-mTOR in AKTIV + Temsirolimus

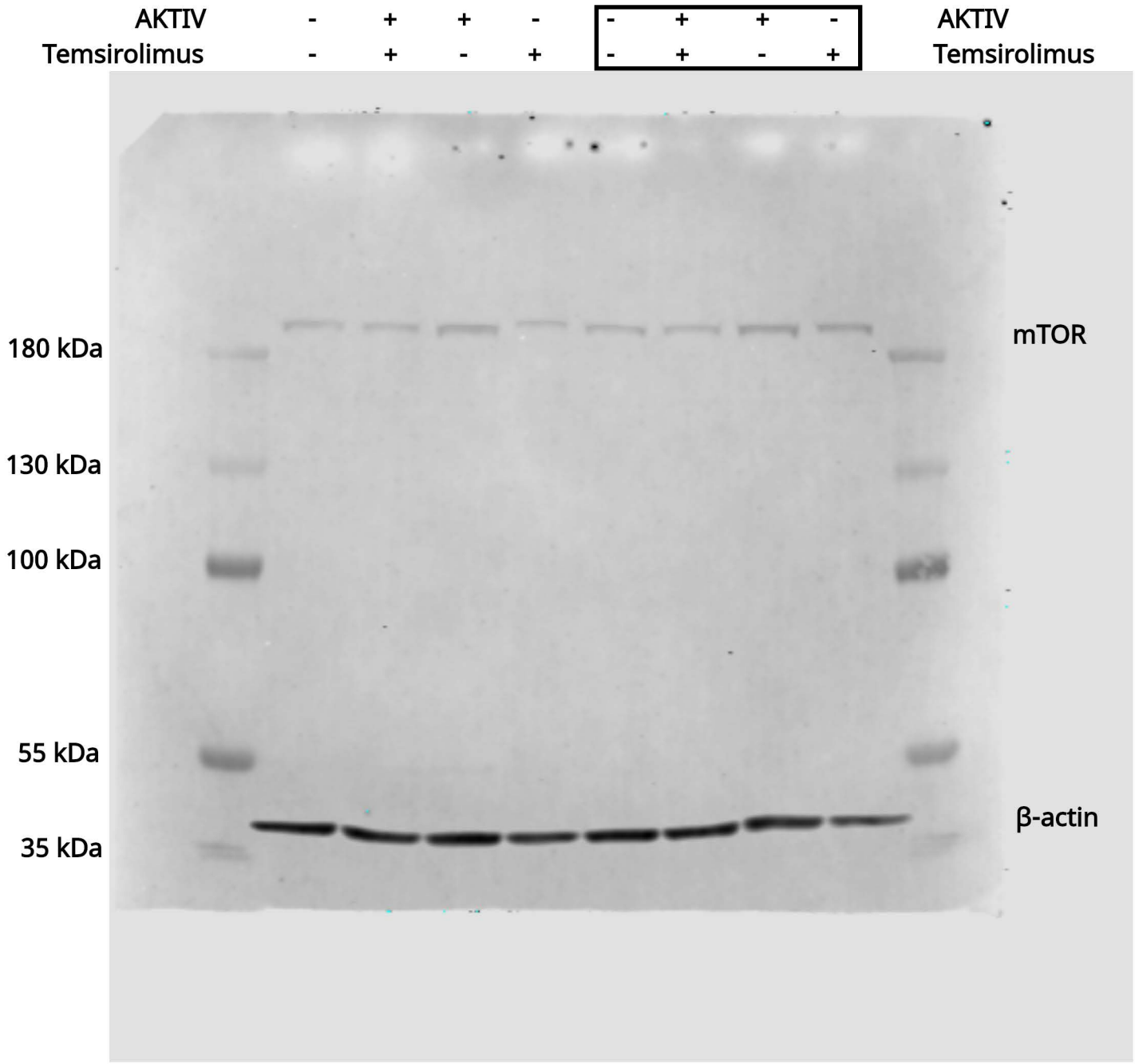

Figure 9A. mTOR in AKTIV + Temsirolimus

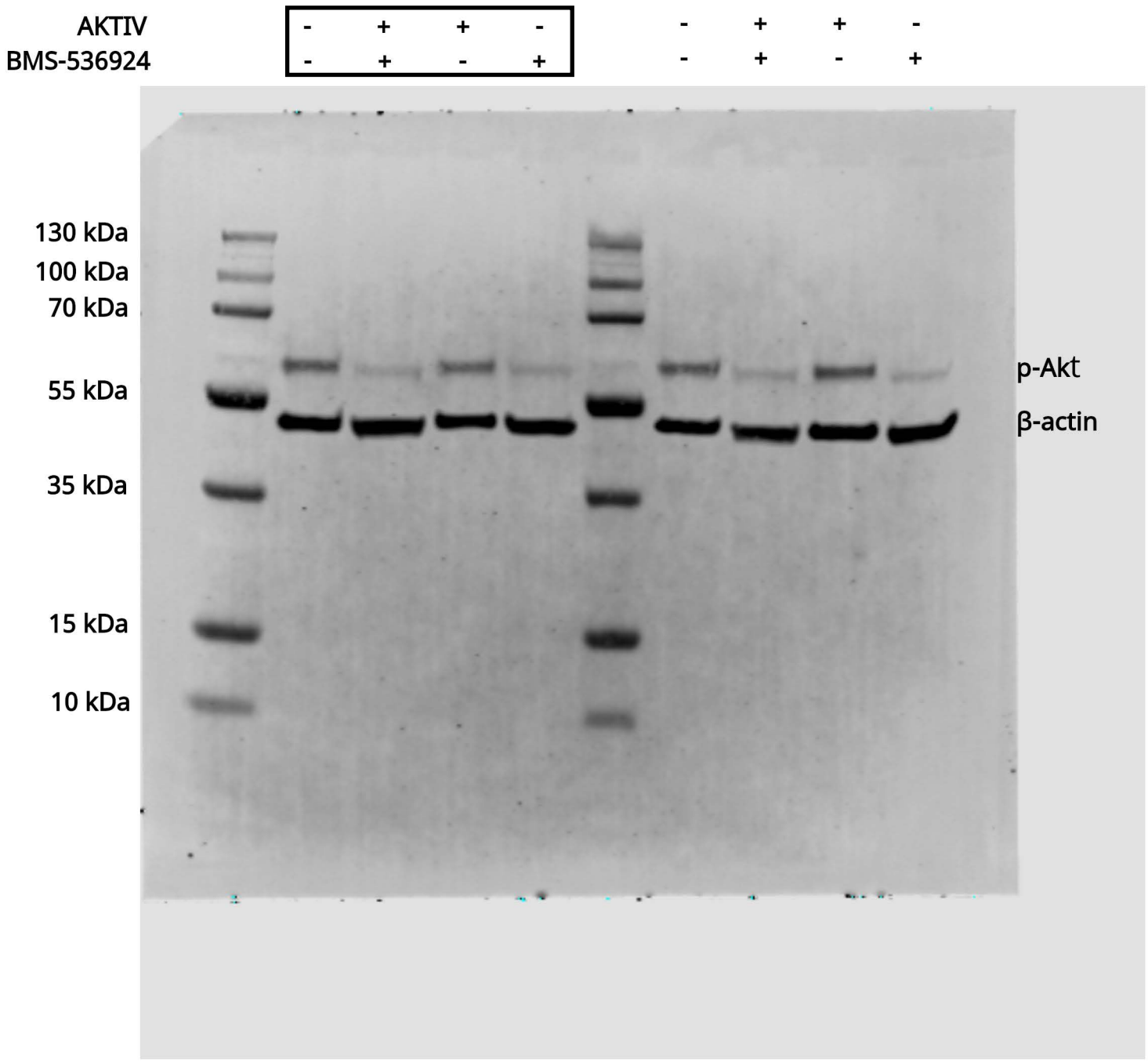

Figure 9B. p-Akt in AKTIV + BMS-536924

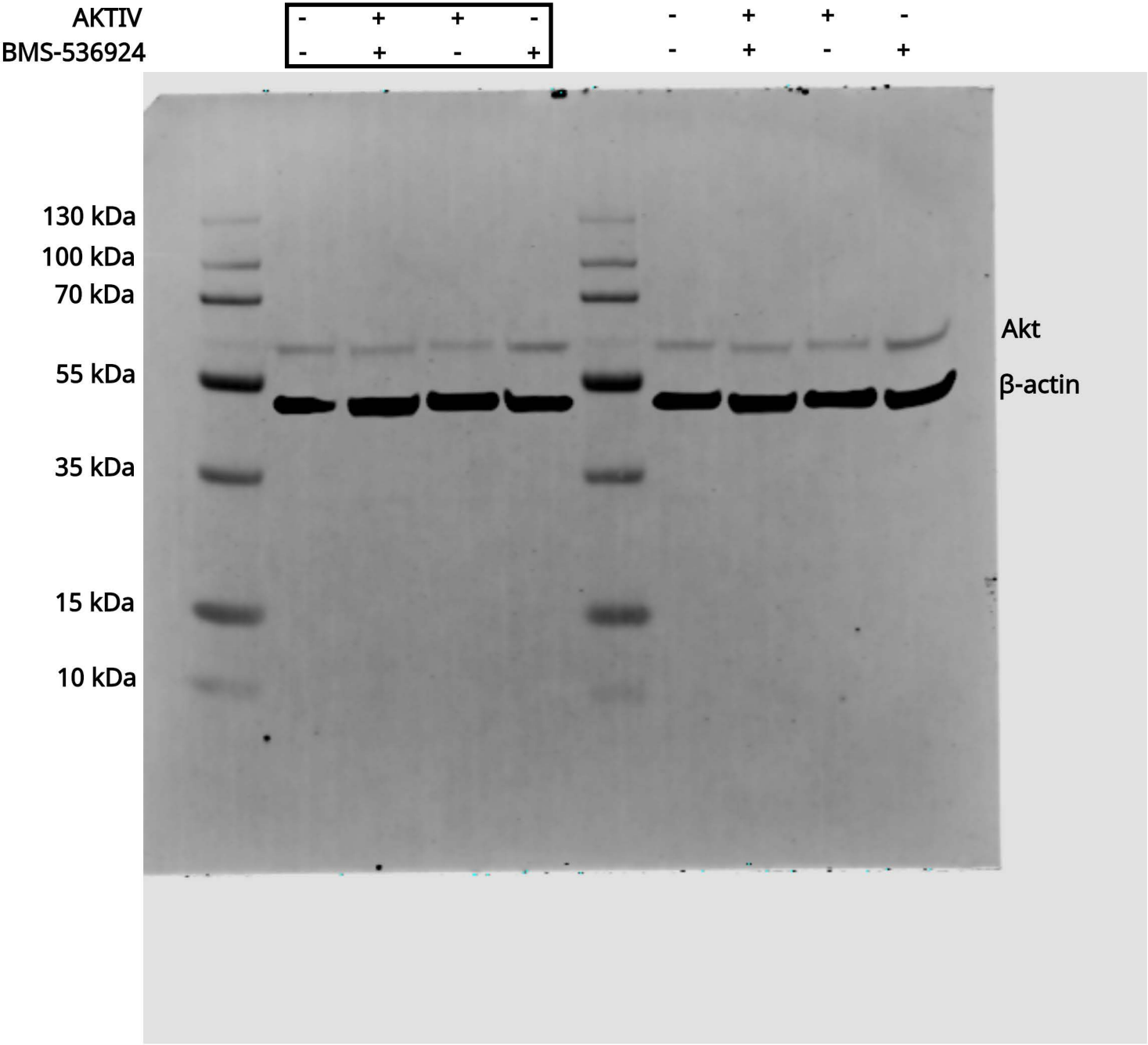

Figure 9B. Akt in AKTIV + BMS-536924

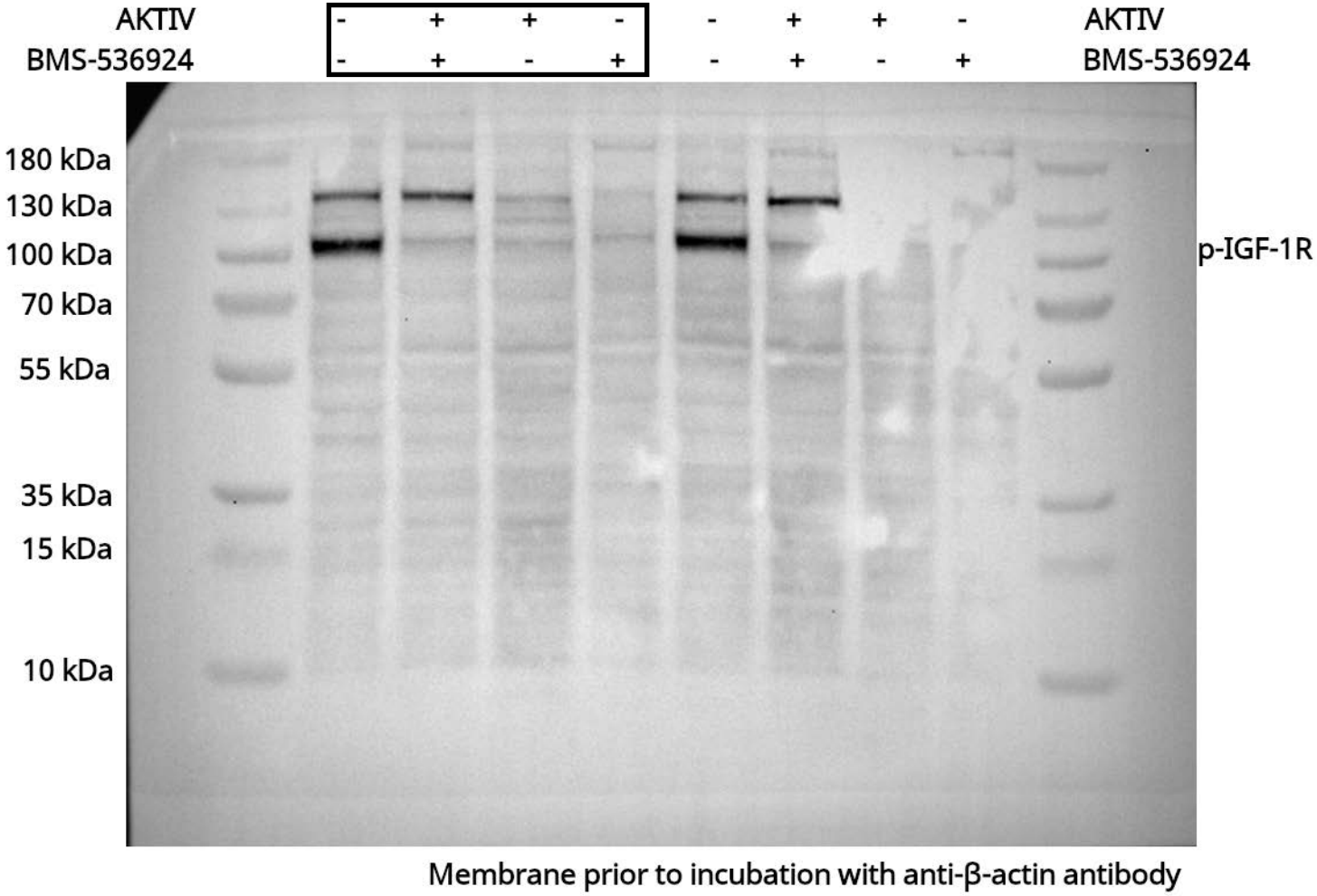

Figure 9B. p-IGF-1R in AKTIV + BMS-536924_before actin

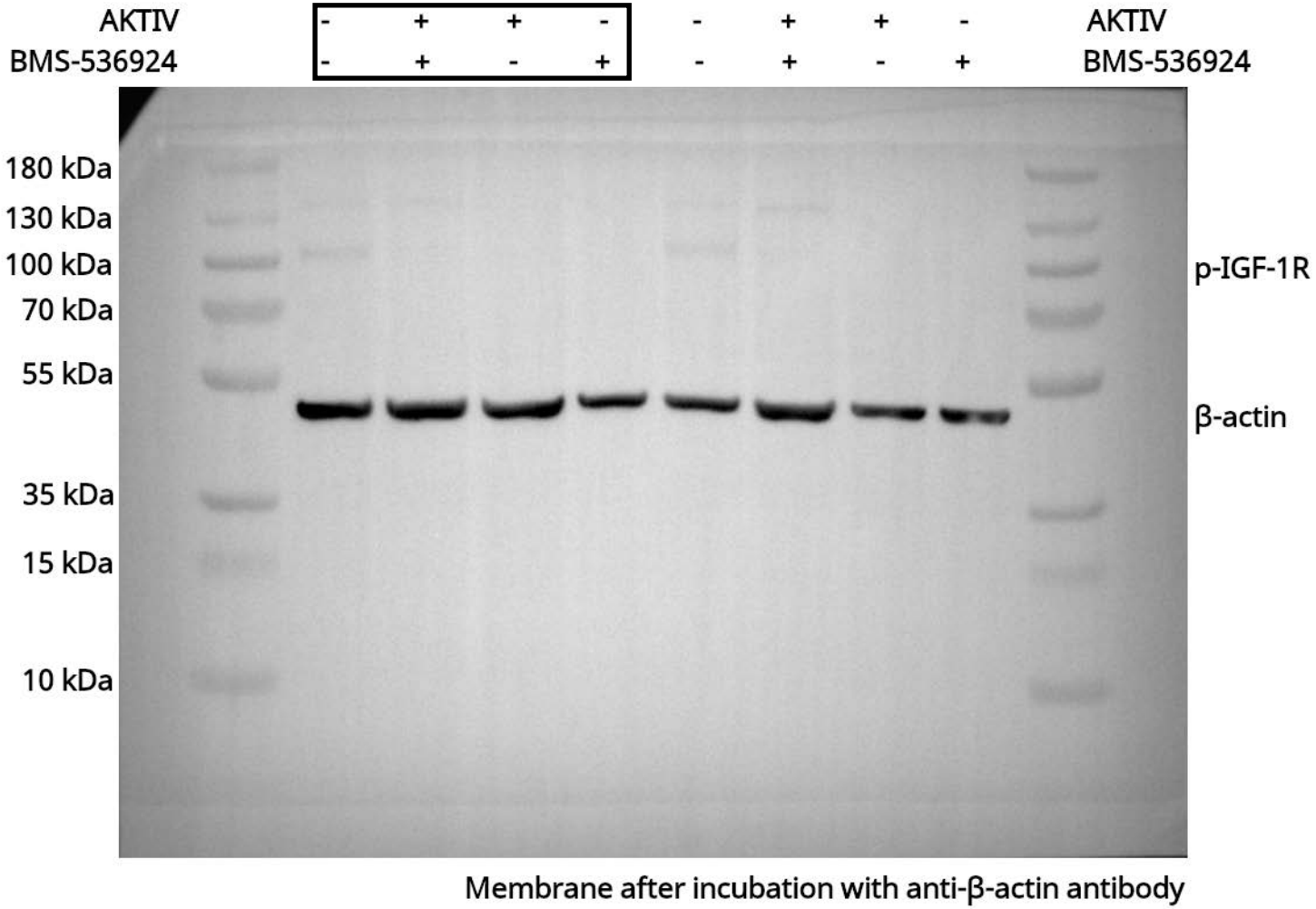

Figure 9B. p-IGF-1R in AKTIV + BMS-536924_after actin

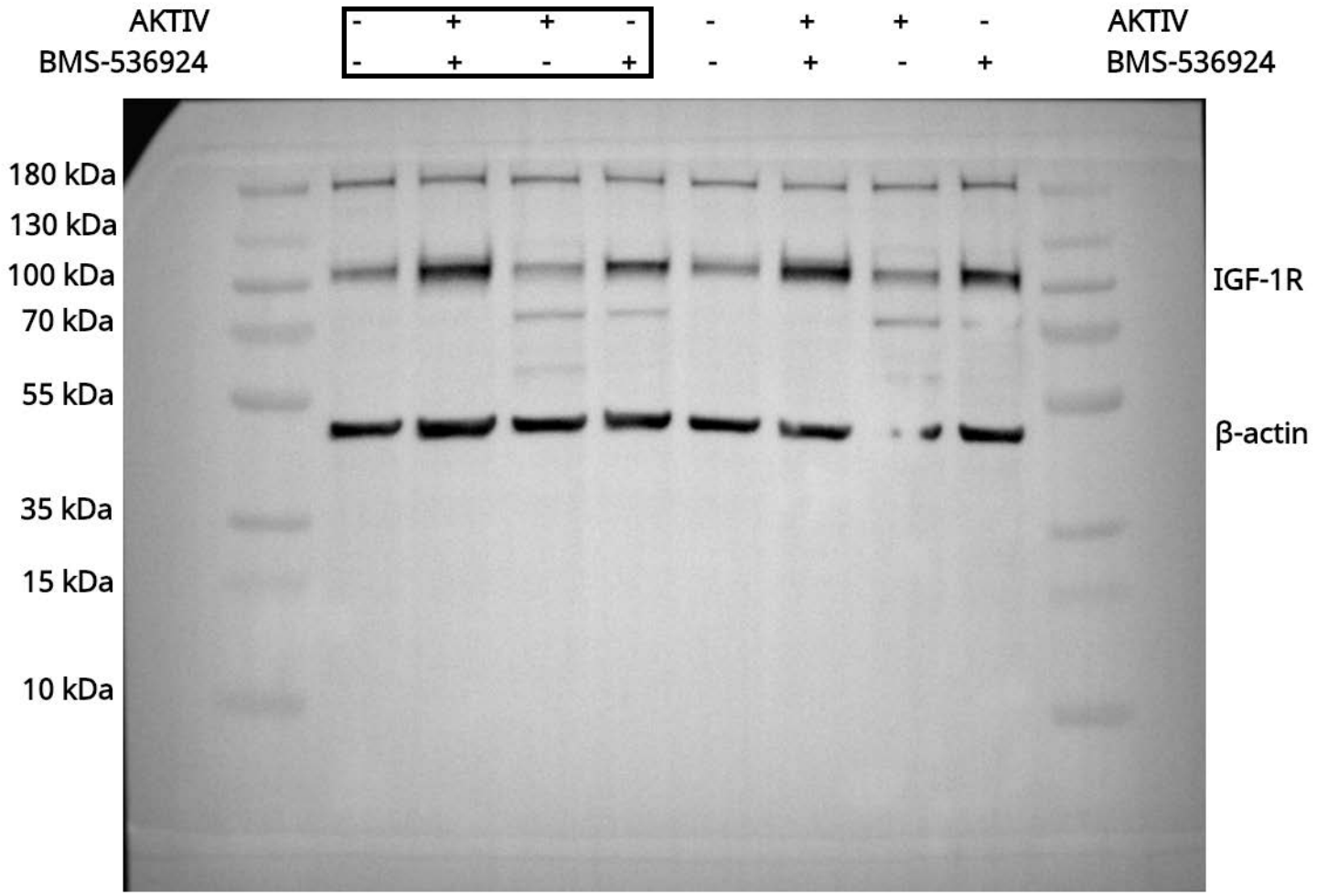

Figure 9B. IGF-1R in AKTIV + BMS-536924

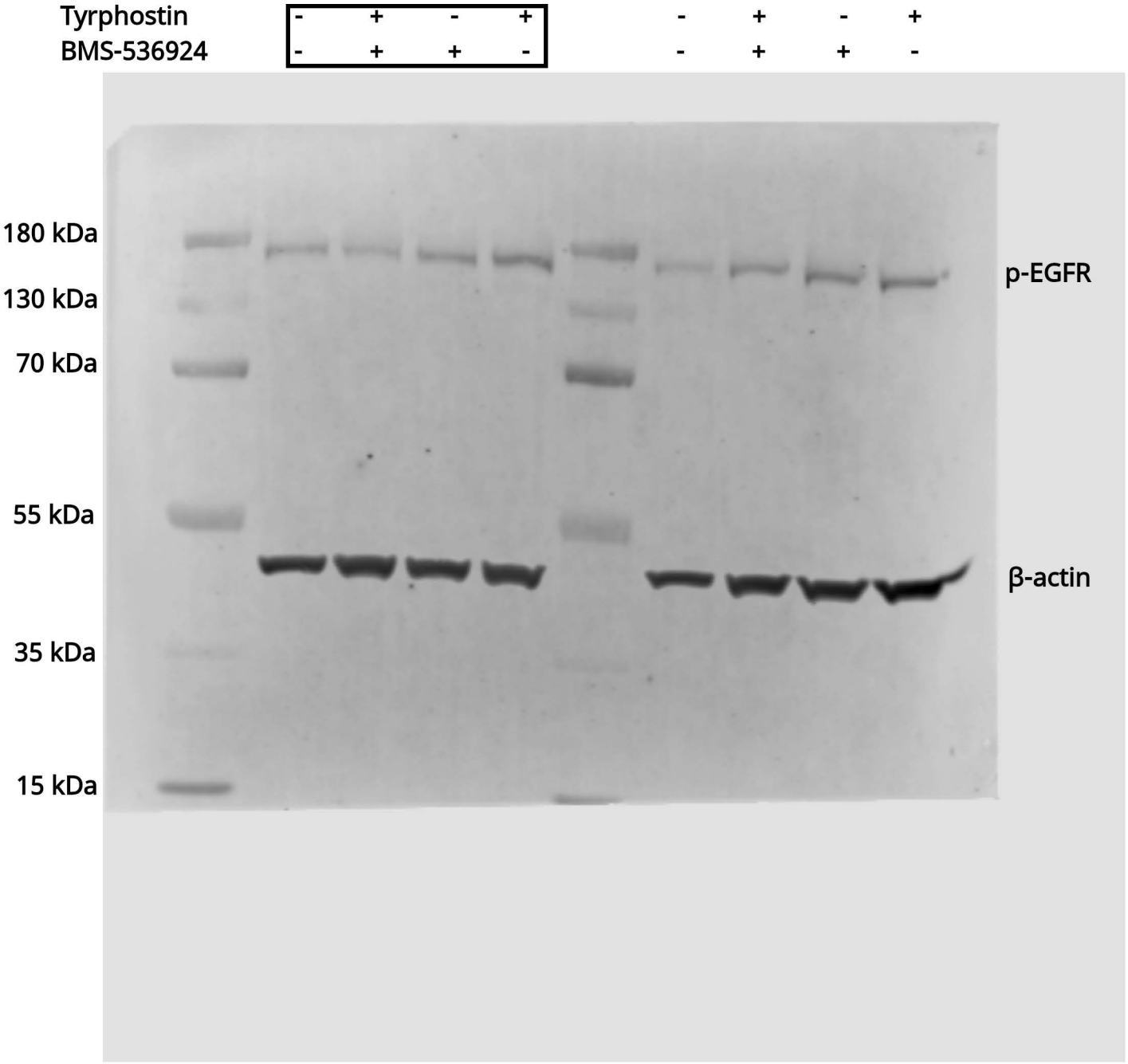

Figure 9C. p-EGFR in Tyrphostin + BMS-536924

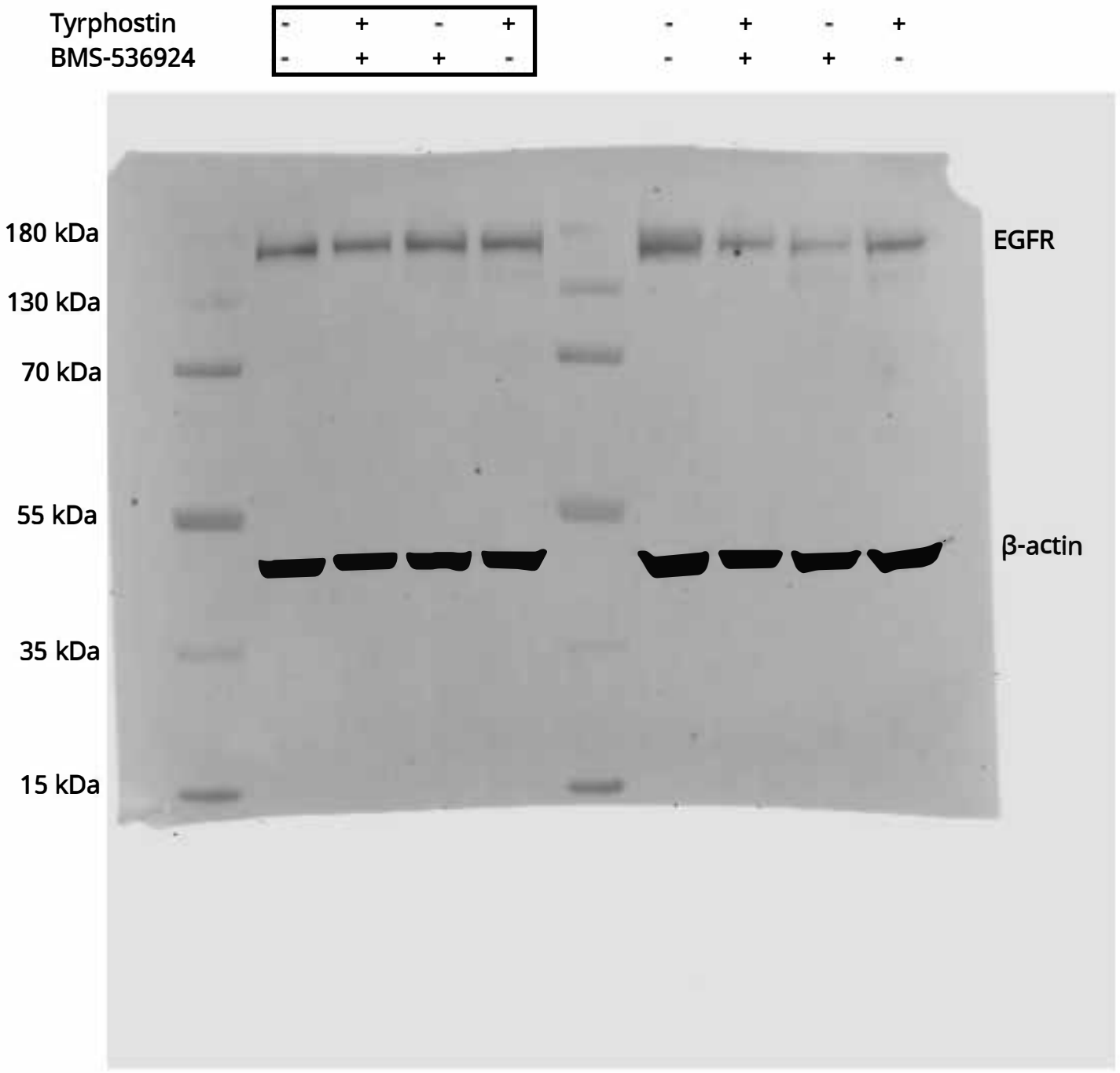

Figure 9C. EGFR in Tyrphostin + BMS-536924

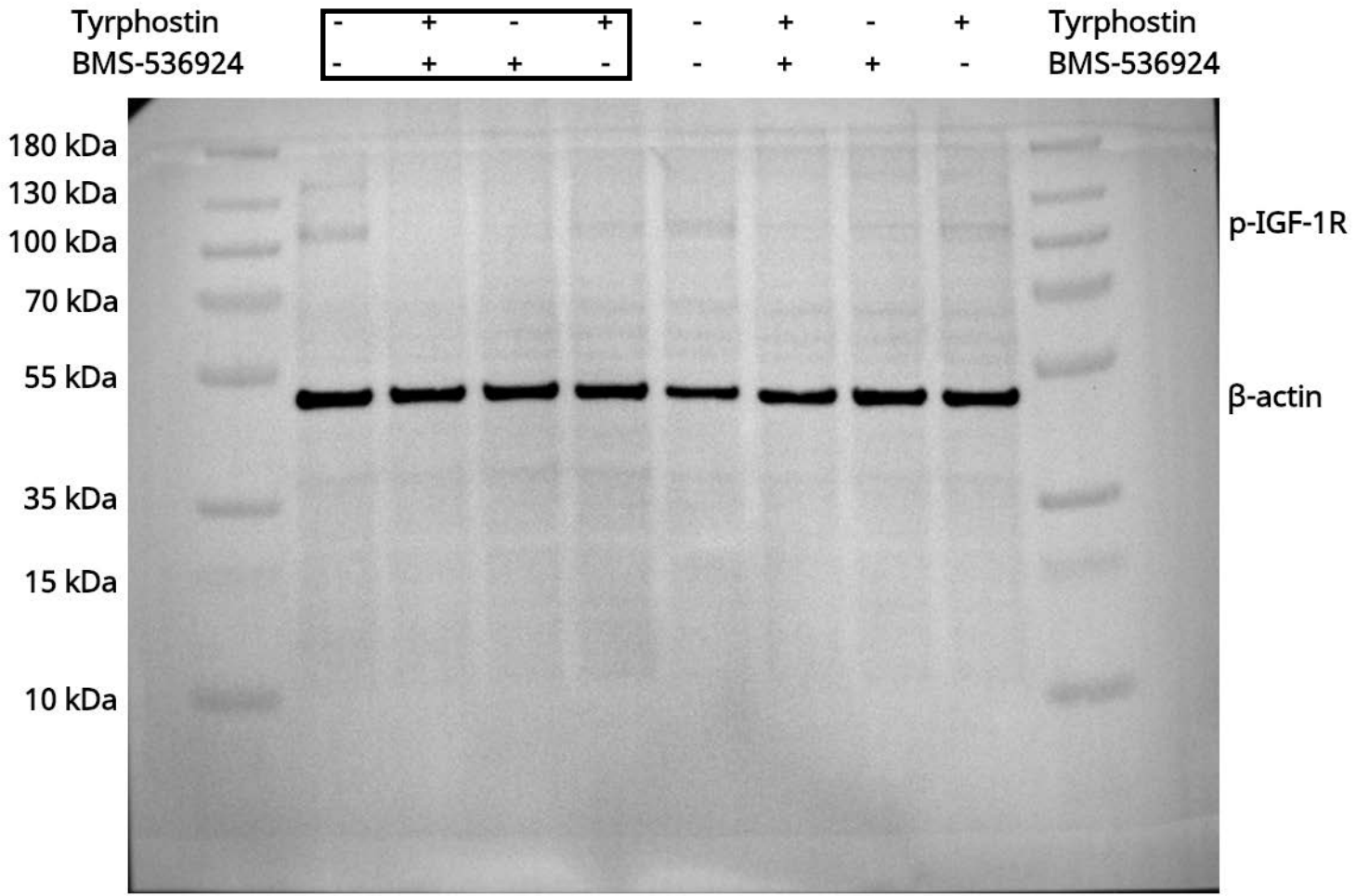

Figure 9C. p-IGF-1R in Tyrphostin + BMS-536924

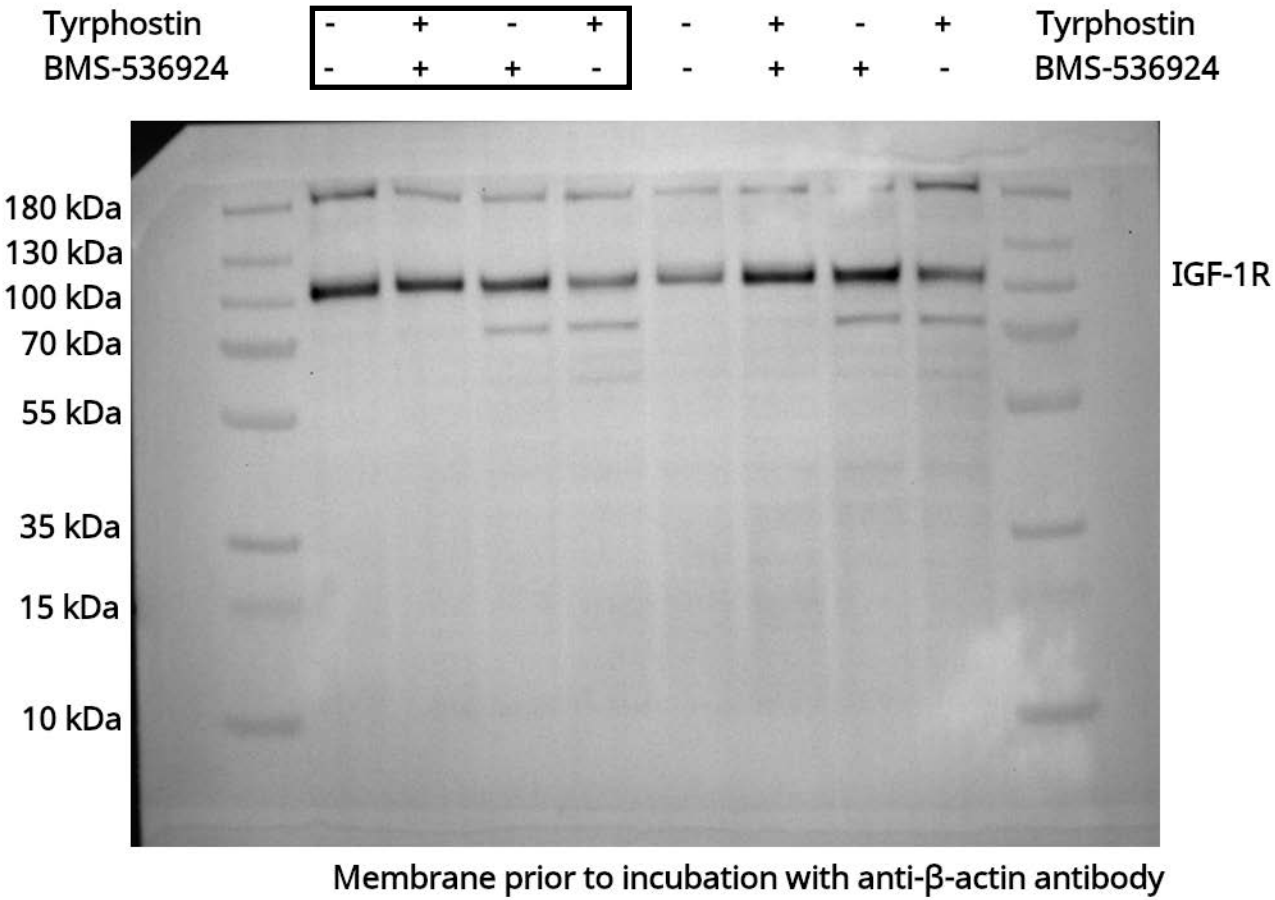

Figure 9C. IGF-1R in Tyrphostin + BMS-536924_before actin

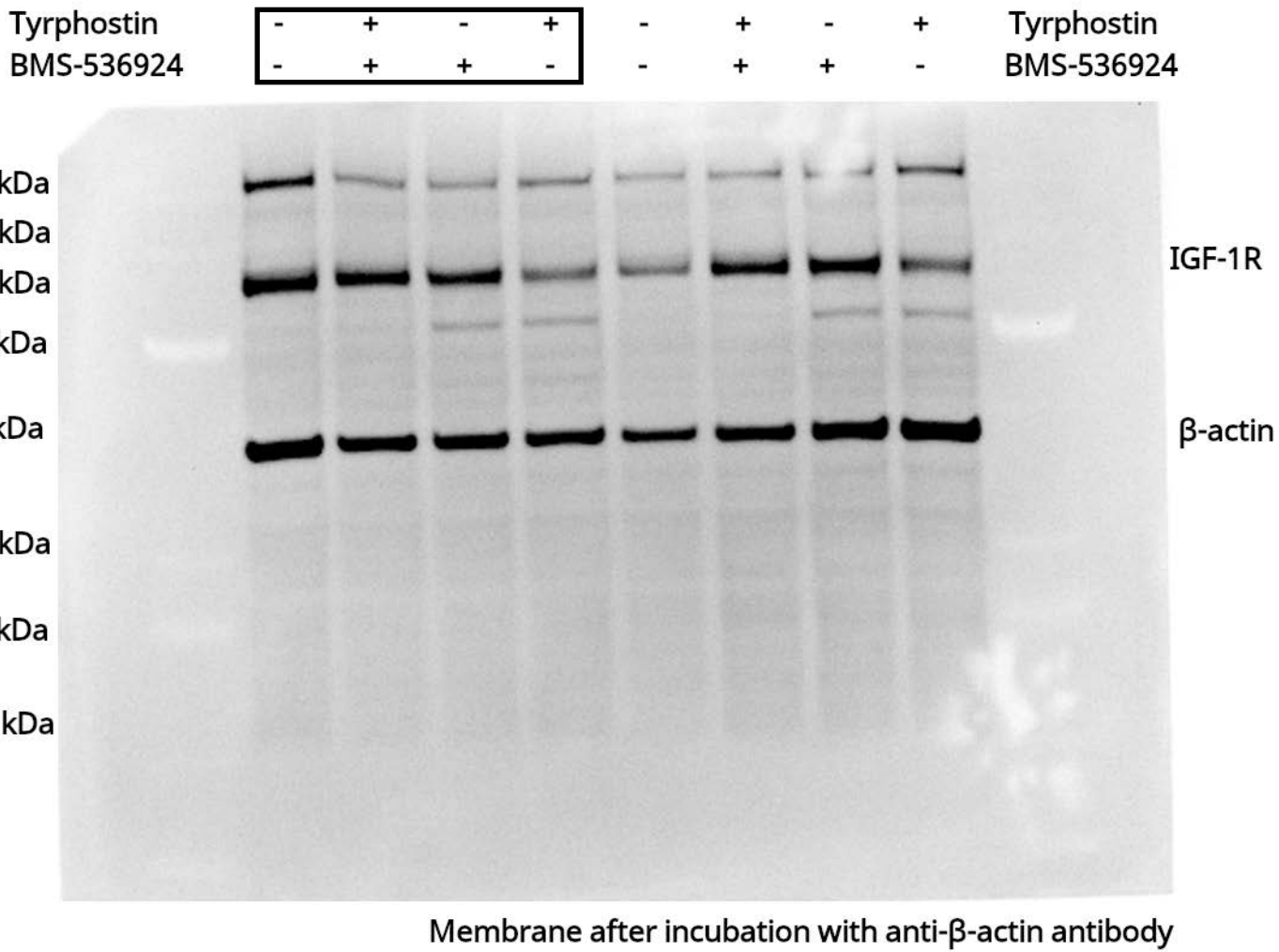

Figure 9C. IGF-1R in Tyrphostin + BMS-536924_after actin

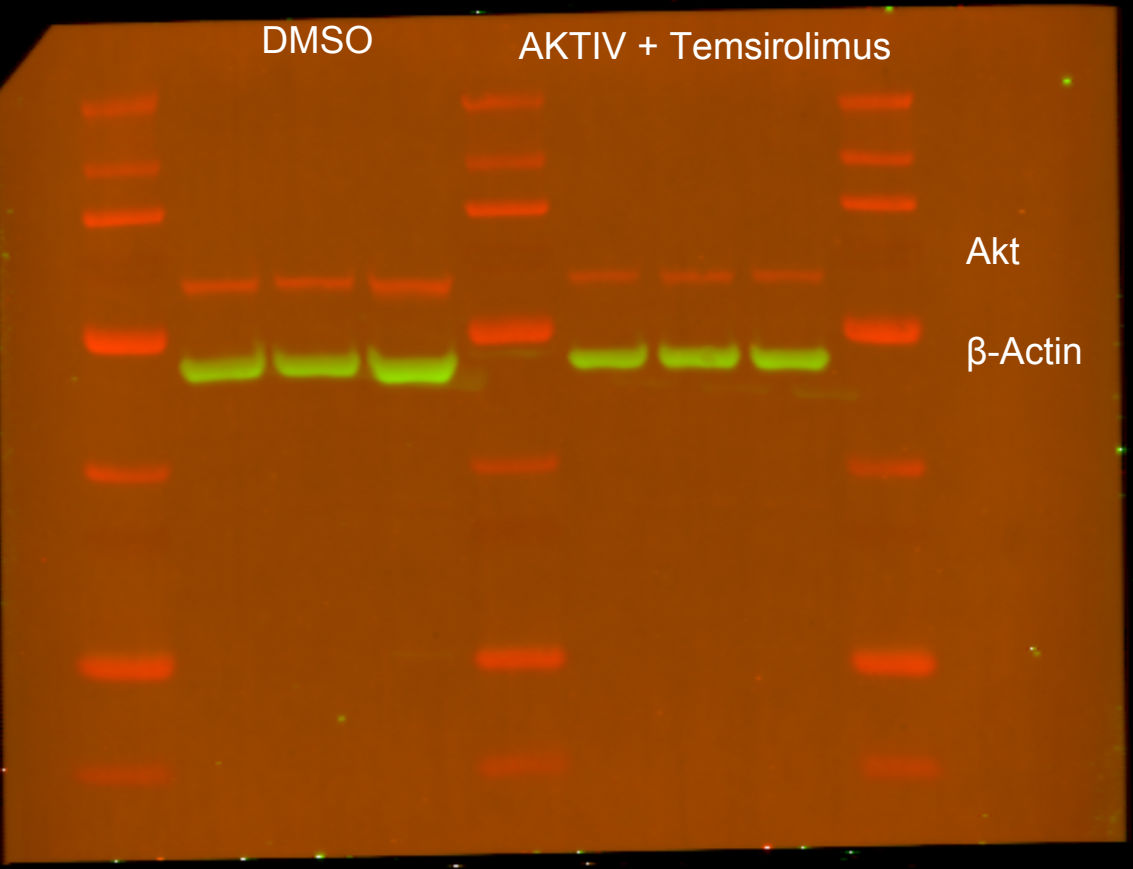

S7 Fig (i). Akt in AKTIV + Temsirolimus

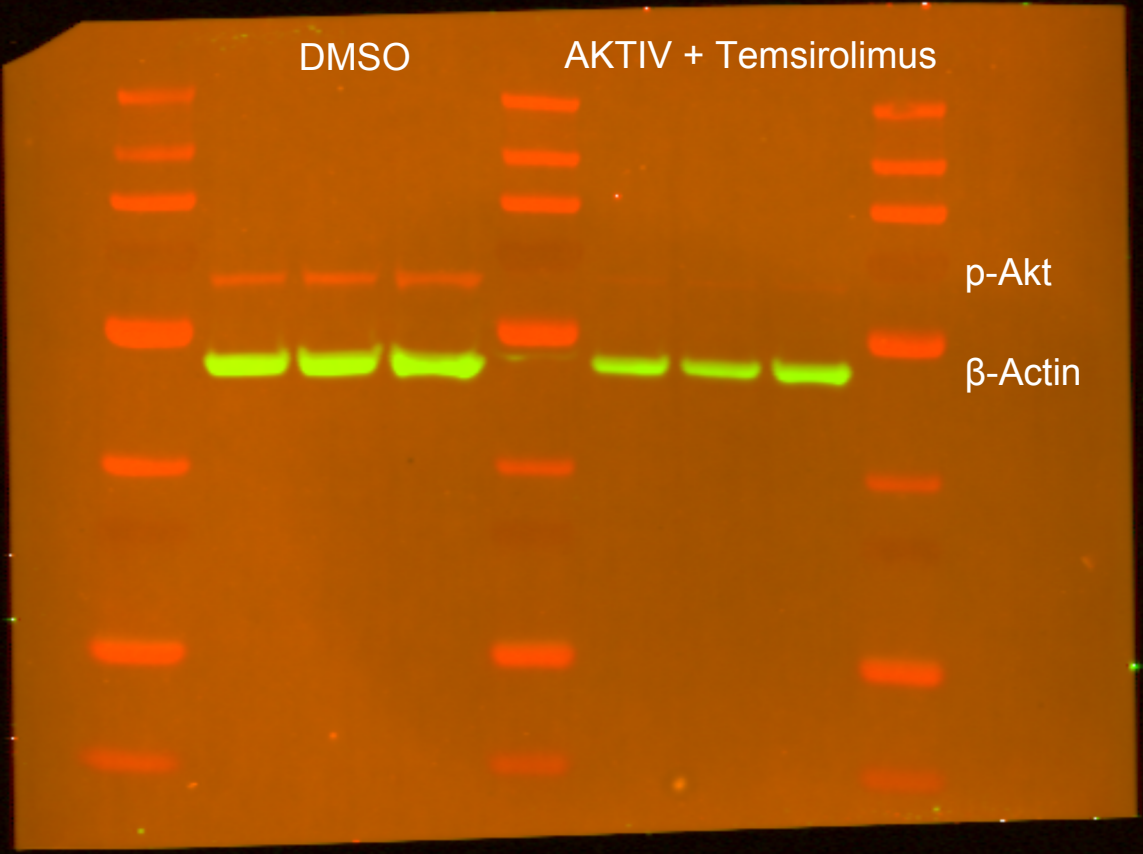

S7 Fig (i). pAkt in AKTIV + Temsirolimus

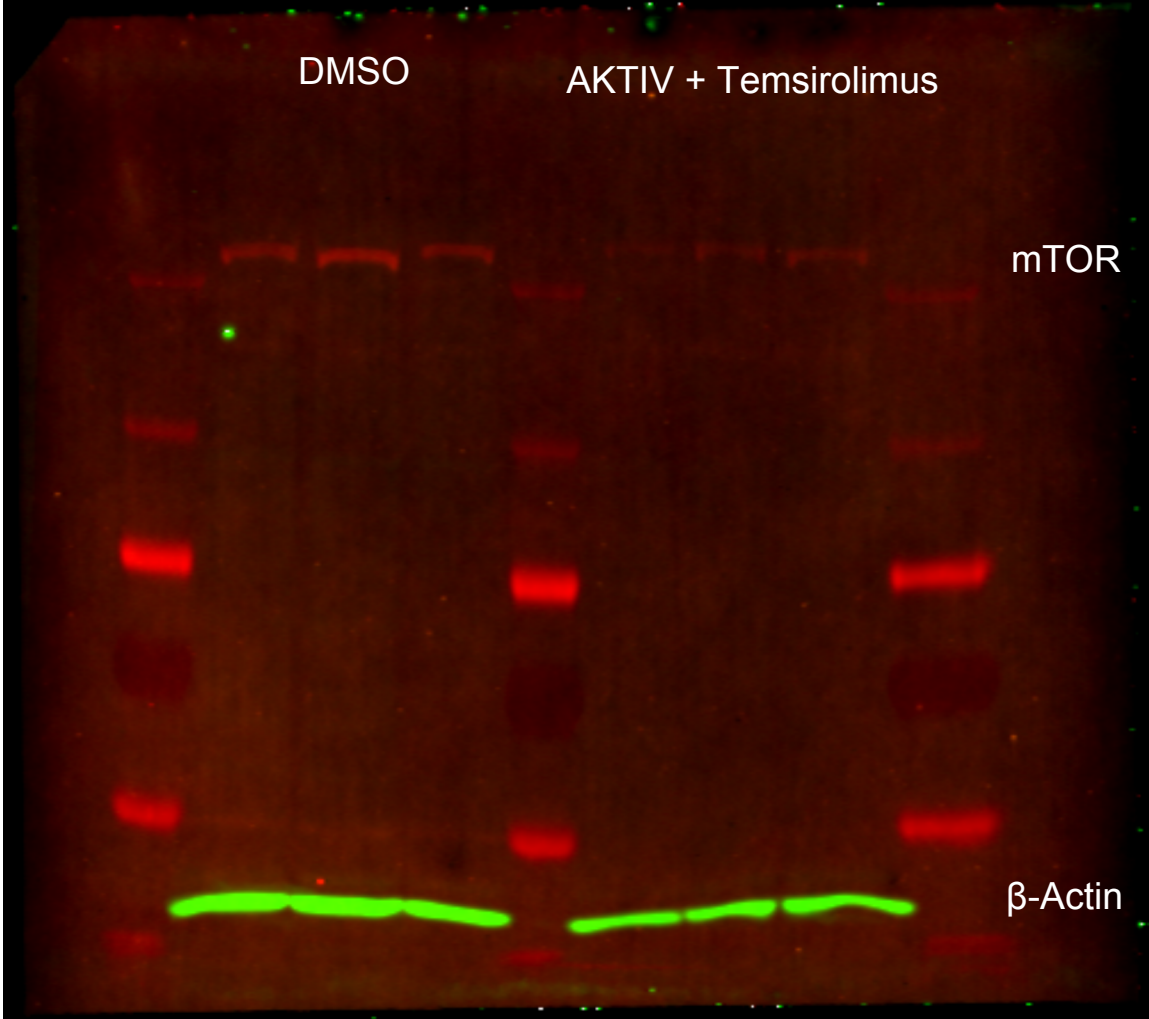

S7 Fig (i). mTOR in AKTIV + Temsirolimus

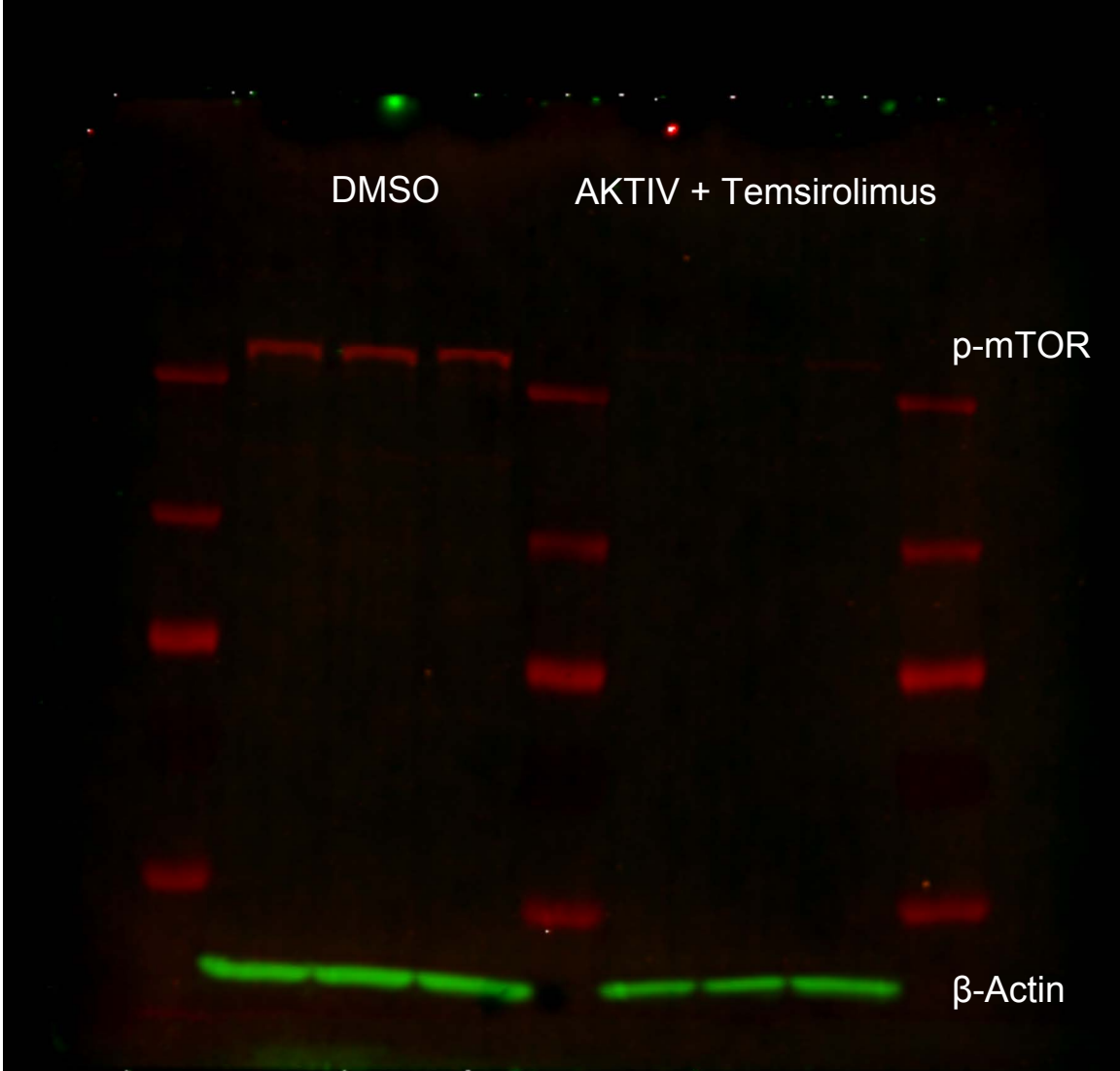

S7 Fig (i). pmTOR in AKTIV + Temsirolimus

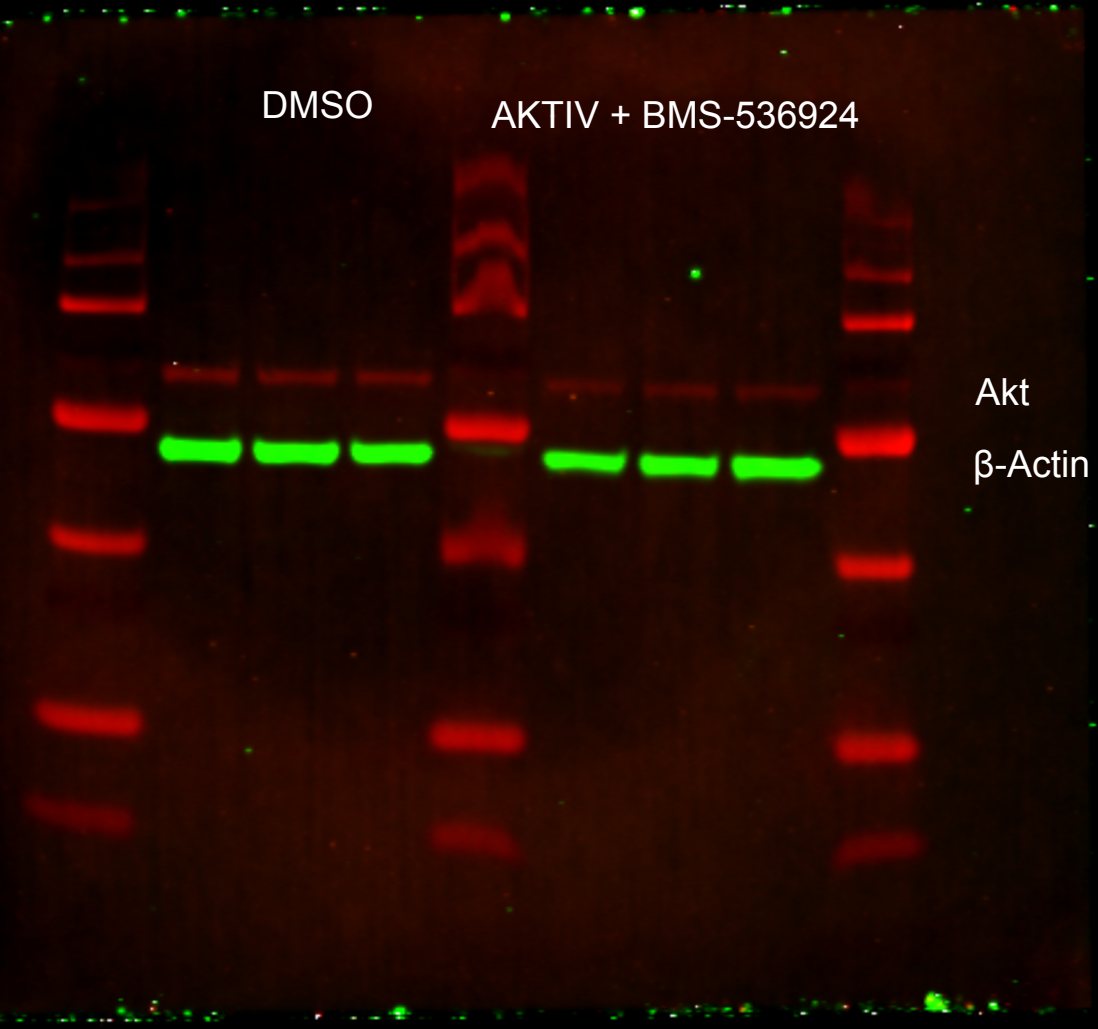

S7 Fig (ii). Akt in AKTIV + BMS-536924

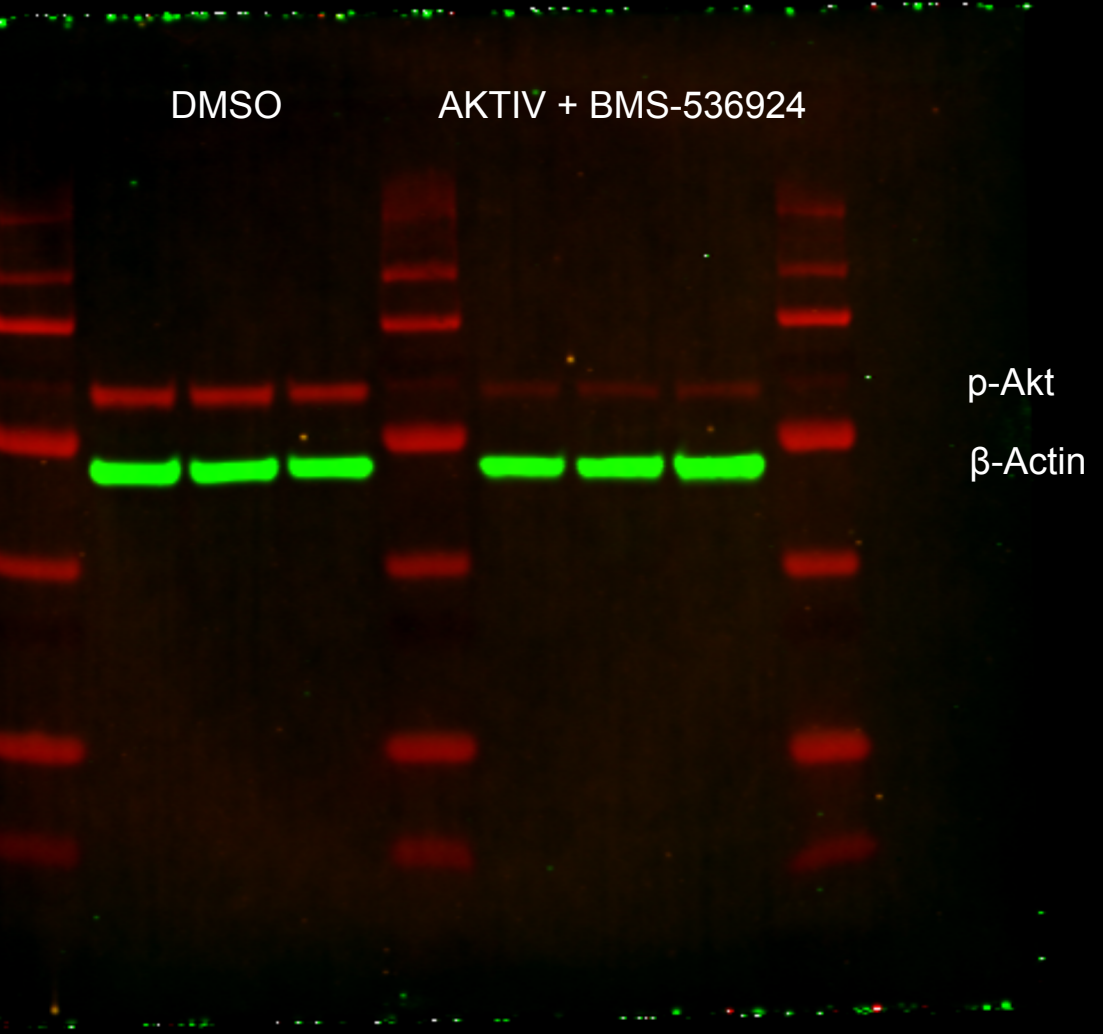

S7 Fig (ii). pAkt in AKTIV + BMS-536924

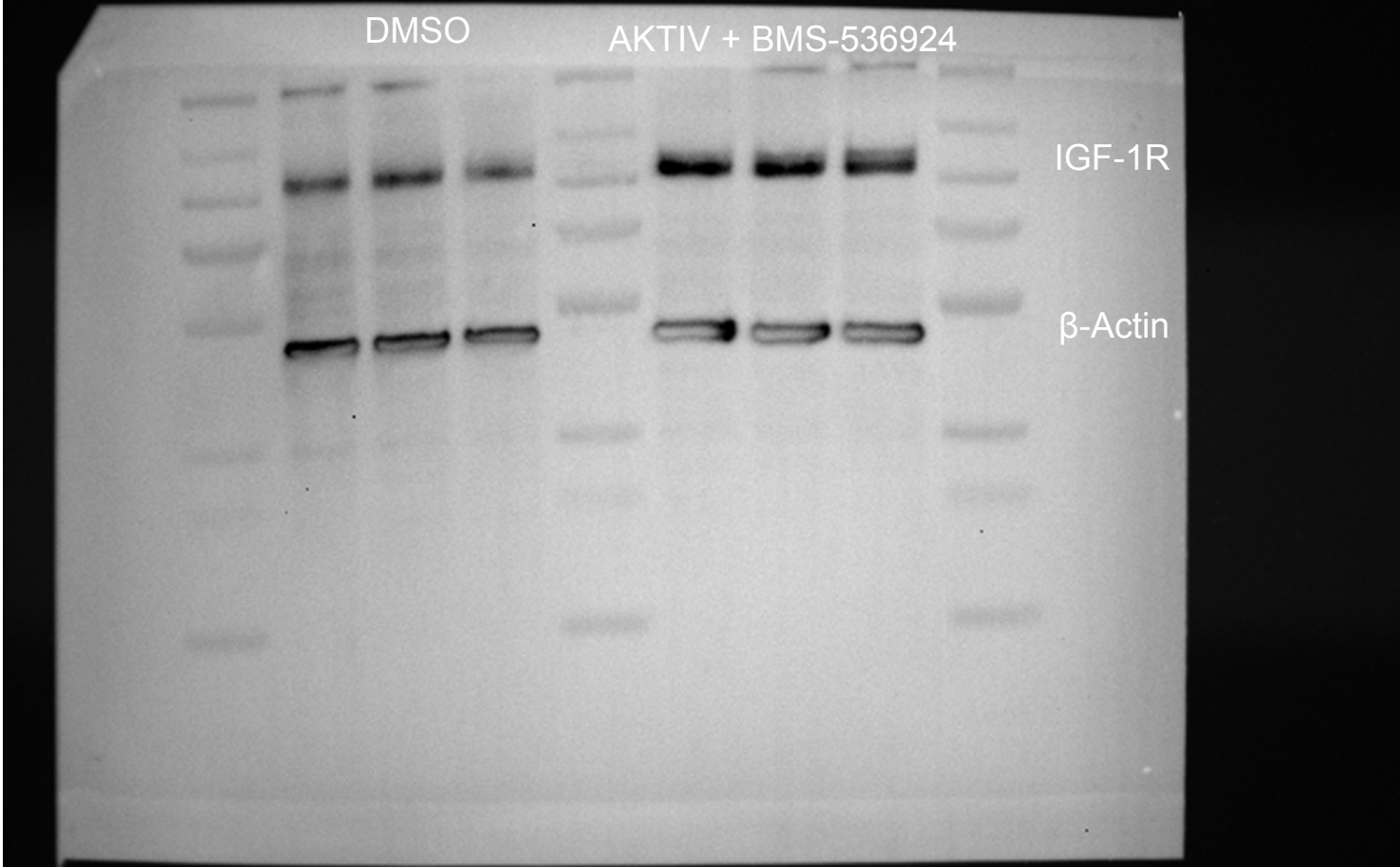

S7 Fig (ii). IGF-1R in AKTIV + BMS-536924

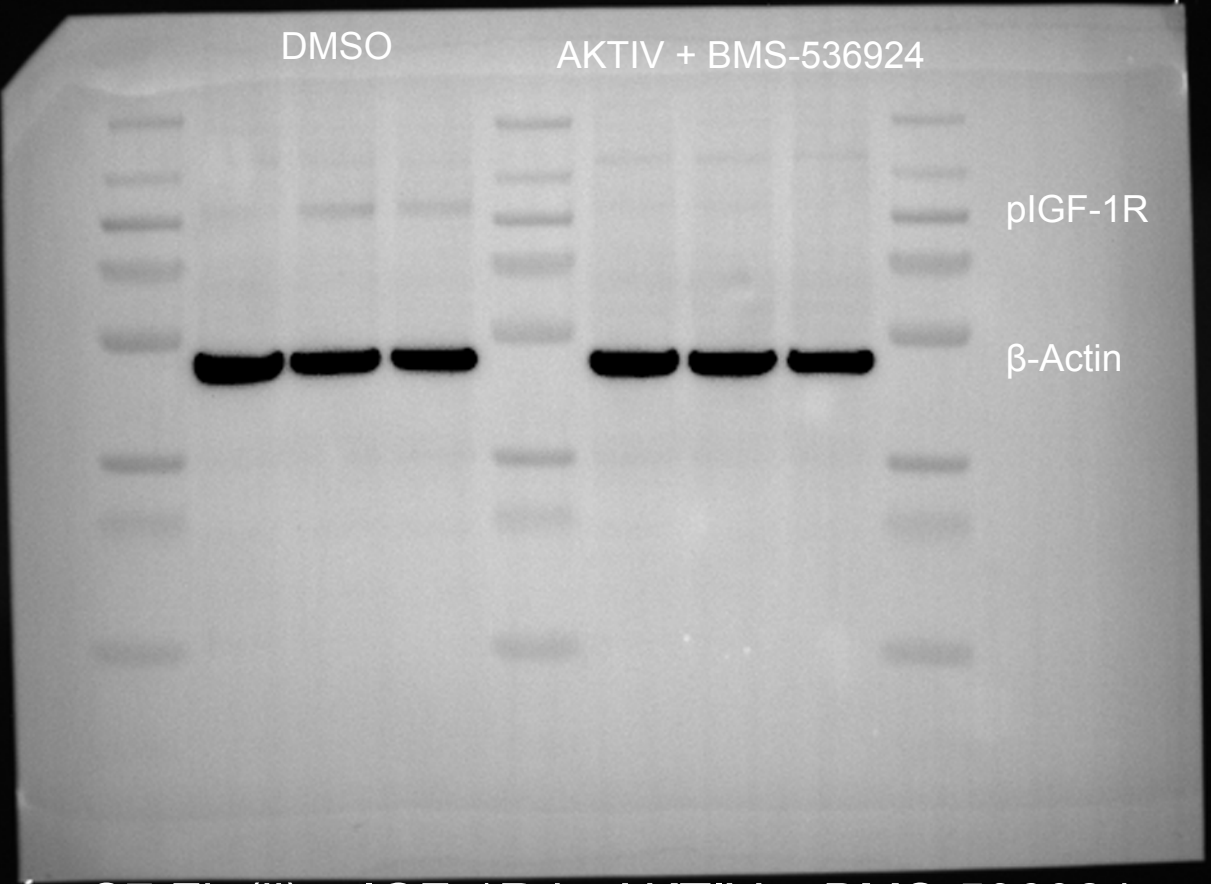

S7 Fig(ii). pIGF-1R in AKTIV + BMS-536924

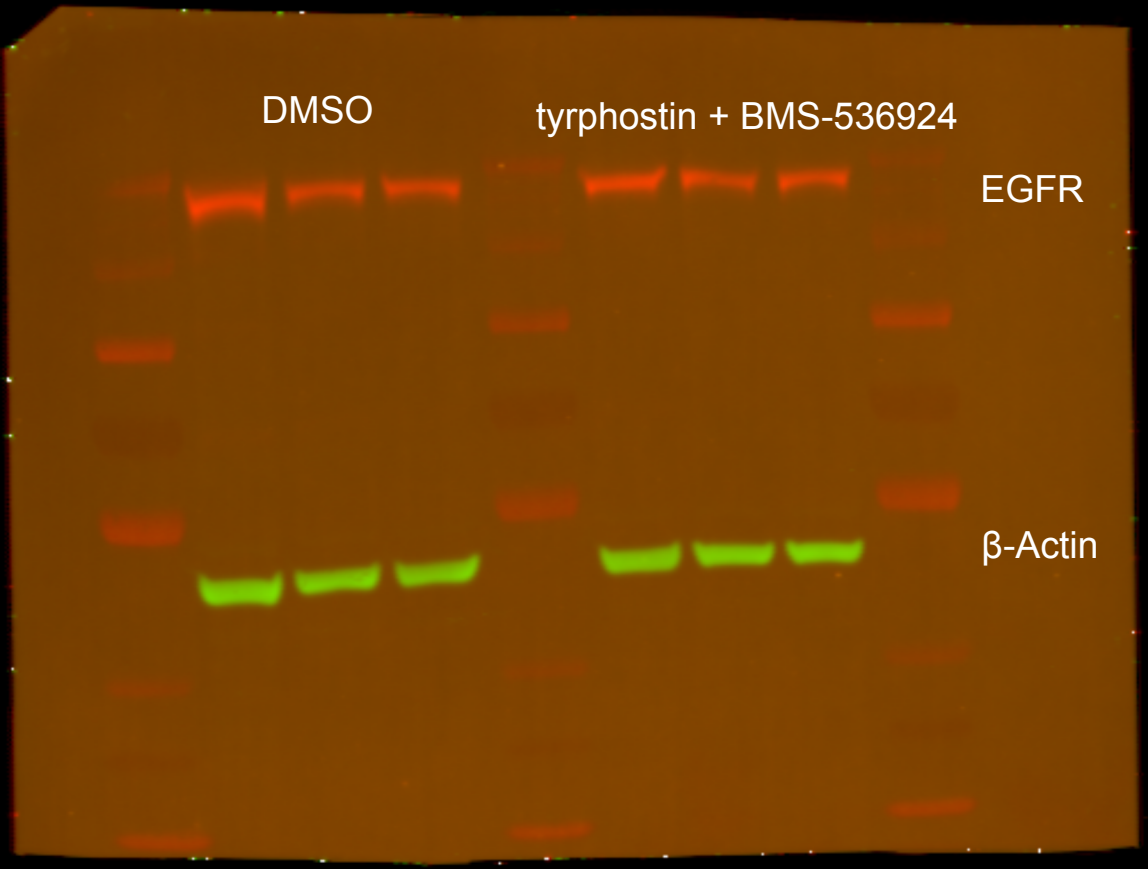

S7 Fig (iii). EGFR in tyrphostin + BMS-536924

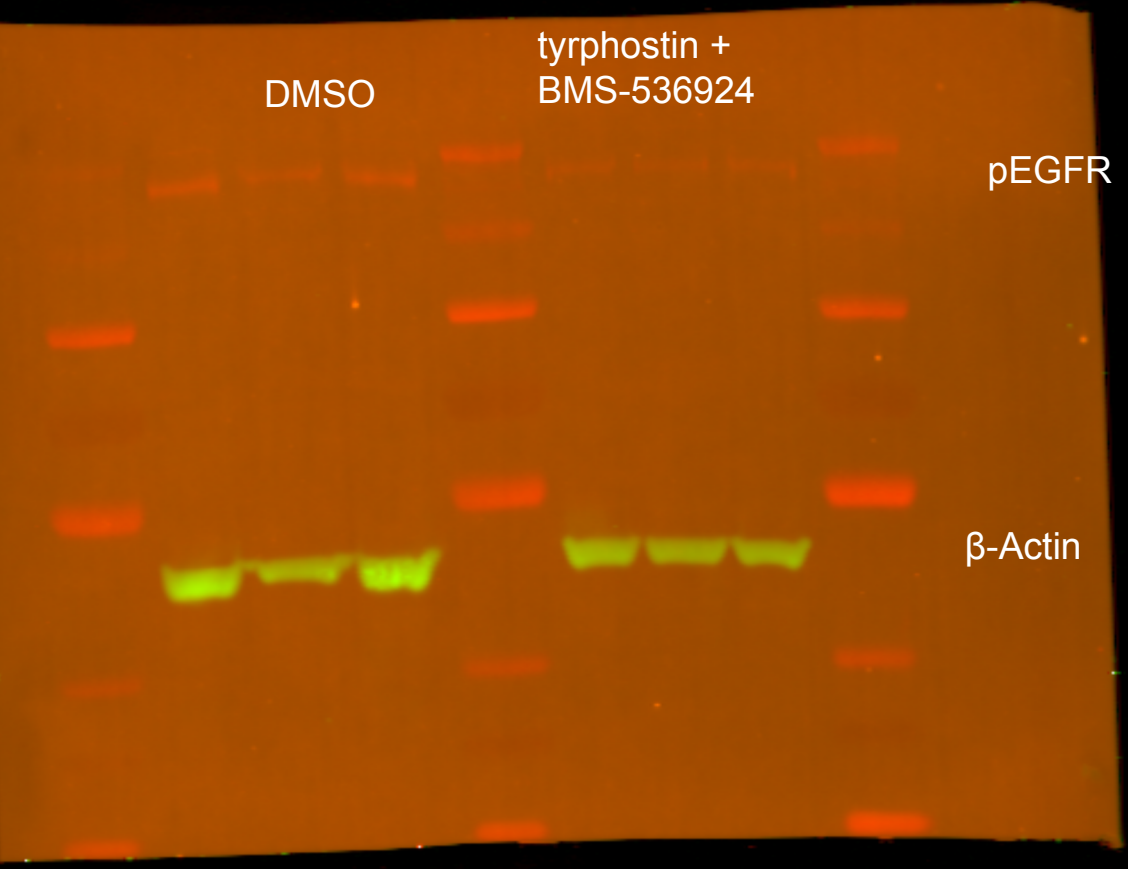

S7 Fig (iii). pEGFR in tyrphostin + BMS-536924

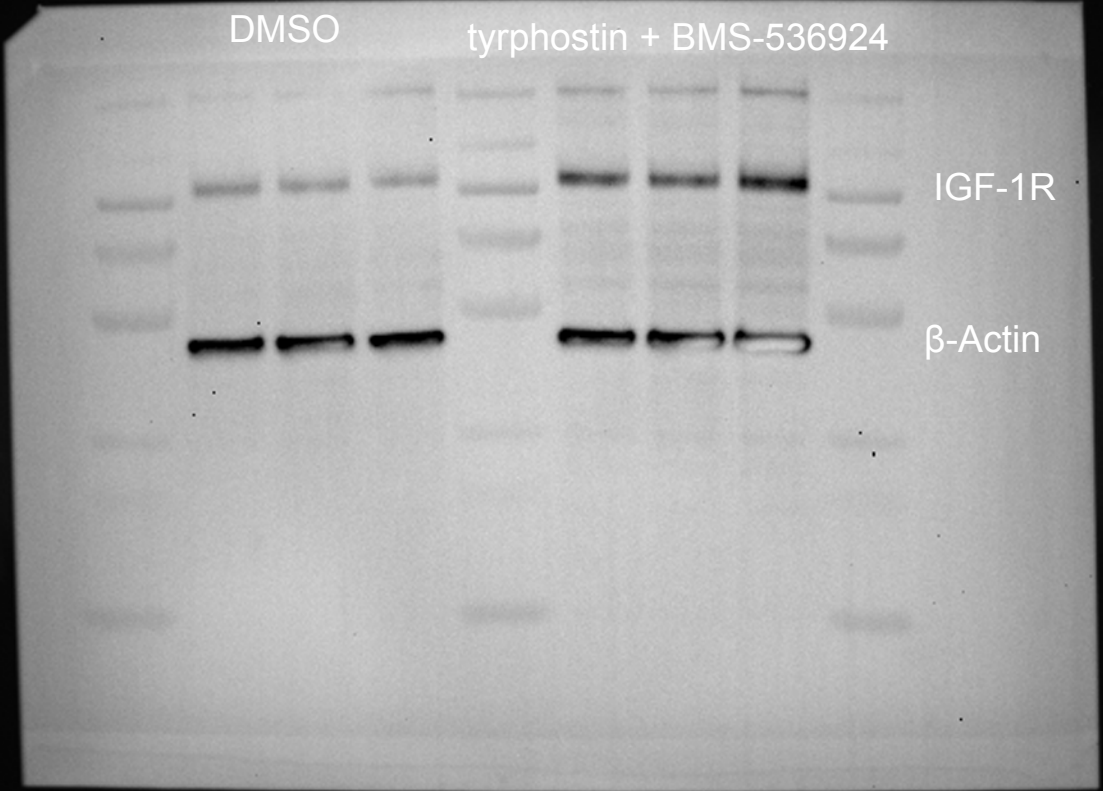

S7 Fig (iii). IGF-1R in tyrphostin + BMS-536924

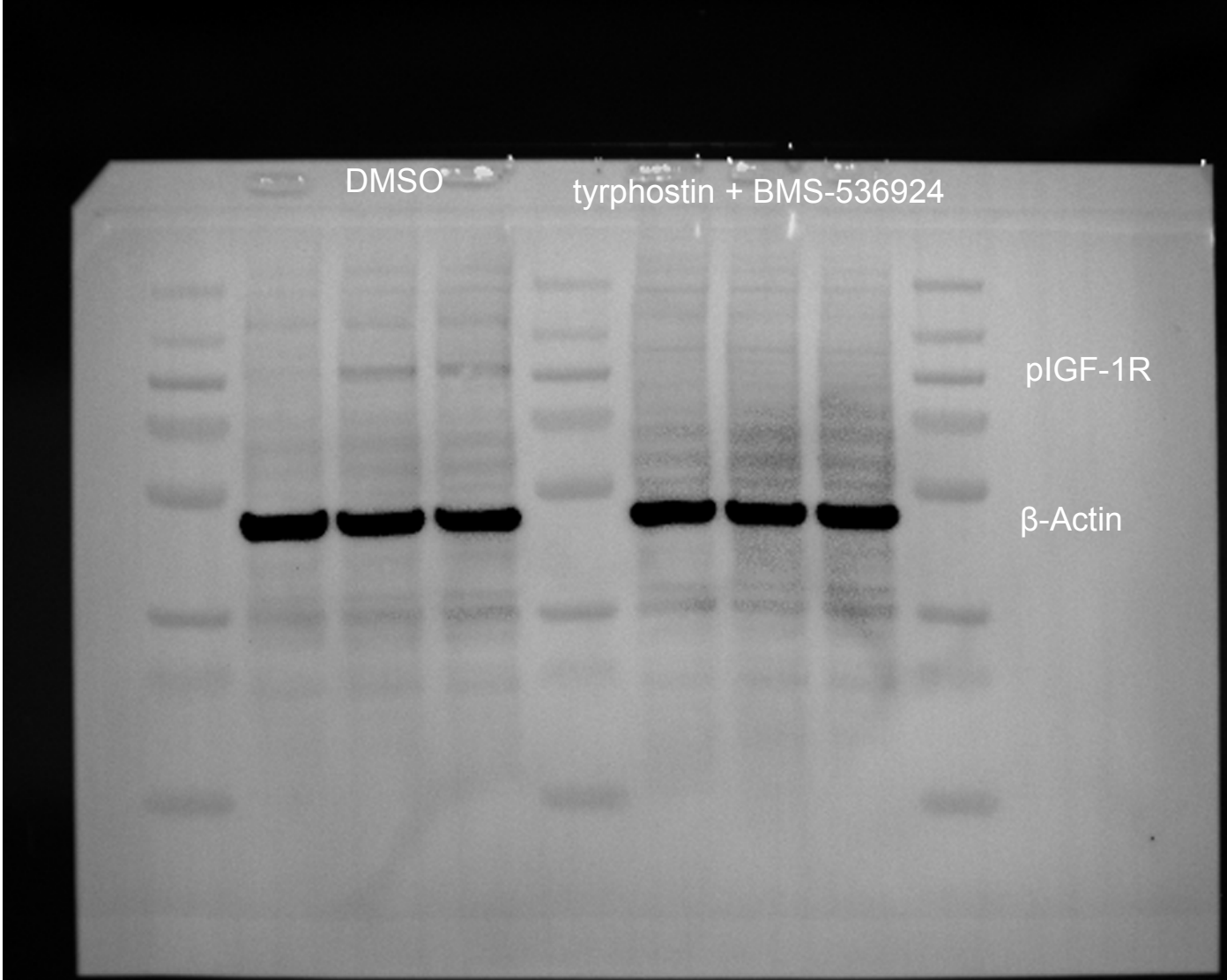

S7 Fig (iii). pIGF-1R in tyrphostin + BMS-536924
