# Supplementary material for: From in silico prediction to experimental validation: Identification of drugs and novel synergistic combinations that inhibit growth of inflammatory breast cancer cells
Source: PLoS One. 2026 Sep 16;21(9):e0339543. doi: 10.1371/journal.pone.0339543 (PMC13581016; doi:10.1371/journal.pone.0339543)
Supplement: Supplementary file 6 [file pone.0339543.s010.docx]

**S1 Table.** **The 24 LWAS-predicted drugs for IBC (from Ji et al., 2020).** The table presents the corresponding drug class, PubMed entry, Clinical Trials entry, and NCT number.

|  | **Drug Name** | **Drug Bank ID ^1^** | **Drug class (MOA) ^2^** | **PubMed for IBC ^3^** | **Clinical Trials for IBC ^4^** | **NCT**  **Number ^5^** |
| --- | --- | --- | --- | --- | --- | --- |
| **Vinca alkaloids** | Vincristine | DB00541 | Microtubule destabilizer | Yes | No | -- |
|  | Vinorelbine | DB00361 | Microtubule destabilizer | Yes | Yes | NCT01325428 |
| **Antimetabolite** | Methotrexate | DB00563 | DNA/RNA damage/inhibits dihydrofolate reductase (DHFR) | Yes | Yes | NCT00003680 |
|  | 5-Fluorouracil | DB00544 | DNA/RNA damage/inhibits  thymidylate synthase (TS) | Yes | Yes | NCT01036087  NCT00976989  NCT02132949 |
|  | Gemcitabine | DB00441 | Inhibits DNA synthesis | Yes | Yes | NCT00193050  NCT00193206 |
|  | Hydroxyurea | DB01005 | Inhibits DNA synthesis | No | No | -- |
|  | Cytarabine | DB00987 | DNA replication inhibitor | No | No | -- |
| **Alkylating Agents** | Carmustine | DB00262 | Alkylates DNA | Yes | No | -- |
|  | Ifosfamide | DB01181 | Alkylates DNA | Yes | No | -- |
|  | Dacarbazine | DB00851 | Alkylates DNA | No | No | -- |
|  | Carboplatin | DB00958 | Platinum-based alkylating agent (DNA crosslinker) | Yes | Yes | NCT01036087  NCT00118053  NCT05093387  NCT00251329 |
| **Anthracycline** | Daunorubicin | DB00694 | Intercalates DNA/ topoisomerase (topo) 2 inhibitor/DNA breakage | Yes | No | -- |
|  | Doxorubicin | DB00997 | Intercalates DNA/ topo 2 inhibitor/DNA breakage | Yes | Yes | NCT00004925  NCT00005822  NCT00016406  NCT00005800 |
| **Topoisomerase inhibitor** | Etoposide | DB00773 | Plant alkaloids (topo 2 inhibitor) | Yes | Yes | NCT00001507 |
|  | Mitoxantrone | DB01204 | (Intercalates DNA/DNA damage/topo inhibitor) | Yes | No | -- |
|  | Topotecan | DB01030 | Topoisomerase 1 inhibitor | No | No | -- |
| **Taxane** | Paclitaxel | DB01229 | Tubulin stabilizer/mitotic inhibitor | Yes | Yes | NCT00111787  NCT01036087  NCT00001507  NCT02132949 |
|  | Docetaxel | DB01248 | Tubulin stabilizer/mitotic inhibitor | Yes | Yes | NCT00193050  NCT00066443  NCT00118053  NCT00017095 |
| **Kinase inhibitor** | Gefitinib | DB00317 | EGFR Tyrosine Kinase (TK) inhibitor | Yes | No | -- |
|  | Imatinib | DB00619 | TK inhibitor (BCR-ABL, KIT, PDGF-R) | Yes | No | -- |
|  | Sunitinib | DB01268 | TK inhibitor (VEGFR/PDGFR/c-kit) | No | Yes | NCT00513695 |
|  | Lapatinib | DB01259 | TK inhibitor (EGFR/HER2) | Yes | Yes | NCT00105950  NCT00558103  NCT00111787  NCT00450892 |
| **Peptide drug** | Octreotide | DB00104 | For metastatic carcinoid tumors and vasoactive intestinal peptide secreting tumors | Yes | No | -- |
| **Glucocort-icoids** | Prednisone | DB00635 | Inhibit NF-Kappa B and other inflammatory transcription factors | Yes | No | -- |

^1^ Drug Bank ID; ^2^ Mechanism of action (MOA); ^3^ Status in PubMed for IBC; ^4^ Clinical Trials for IBC; ^5^ National Clinical Trial Identifier Number.
