# Supplementary material for: From in silico prediction to experimental validation: Identification of drugs and novel synergistic combinations that inhibit growth of inflammatory breast cancer cells
Source: PLoS One. 2026 Sep 16;21(9):e0339543. doi: 10.1371/journal.pone.0339543 (PMC13581016; doi:10.1371/journal.pone.0339543)
Supplement: Supplementary file 7 [file pone.0339543.s011.docx]

**S2 Table.** **The 19 GRR-predicted drugs/compounds (from Ji et al., 2023)**. The table shows with the corresponding drug class for each drug and its mechanism of action.

| **Drug Name** | **Drug ID**  **(PubChem) ^1^** | **Drug Class ^2^** |
| --- | --- | --- |
| **AG-14361** | 9840076 | DNA damage, anti-cancer → PARP-1 inhibitor |
| **AKTIV** | 5719375 | Cytotoxic and antiproliferative → Akt protein kinase Inhibitor |
| **Ambrisentan** | 6918493 | Vasodilator Endothelin → Receptor, GPCR &amp; G Protein |
| **AZD-7545** | 16741245 | Adenocarcinoma → Selective inhibitor of PDHK2 |
| **BMS-536924** | 135440466 | Anti-cancer → IGF1-R kinase and IR inhibitor |
| **BMS-754807** | 24785538 | Anti-cancer → reversible IGF1-R/IR inhibitor |
| **Butalbital** | 2481 | CNS depressant → JAK/STAT  signaling, P53 signaling, and NOTCH  signaling pathway |
| **Clobenpropit** | 2790 | Anti-tumor → Histamine H3 receptor, PI3K/AKT pathway |
| **COT-10b** |  | Acute myeloid leukemia → Serine/threonine MAP3 kinase |
| **Dapsone** | 2955 | Antibacterial → Sulfone drug  Anti-inflammatory → (Not fully understood) |
| **Letrozole** | 3902 | Aromatase Inhibitor  Antineoplastic Agent |
| **Medetomidine** | 68602 | Neurological Disease/Psychotic  Disorders → selective α2-adrenoceptor agonist |
| **Nicardipine** | 4474 | Cardiovascular Disease → Calcium channel blocker |
| **Nimesulide** | 4495 | Selective COX-2 inhibitor (NSAID) |
| **Temsirolimus** | 6918289 | Antineoplastic (mTOR inhibitor) Immunomodulating agent |
| **Ticarcillin** | 36921 | Infection → Beta lactam antibiotic |
| **Tipifarnib-P2** | 159324 | Antineoplastic → Farnesyltransferase inhibitors |
| **Tyrphostin-AG-1478** | 2051 | Histiocytic Lymphoma → EGFR Tyrosine Kinase Inhibitor |
| **ZM336372** | 5730 | Histiocytic Lymphoma → Inhibitor of the MAP protein kinase c-Raf, JAK/STAT signaling. |

^1^PubChemID, ^2^Drug Class.
