# Supplementary material for: From in silico prediction to experimental validation: Identification of drugs and novel synergistic combinations that inhibit growth of inflammatory breast cancer cells
Source: PLoS One. 2026 Sep 16;21(9):e0339543. doi: 10.1371/journal.pone.0339543 (PMC13581016; doi:10.1371/journal.pone.0339543)
Supplement: Supplementary file 8 [file pone.0339543.s012.docx]

**S3 Table.** **Efficacy of LWAS and GRR predicted drugs and compounds in four breast cancer cell lines using the MTT assay.** The table lists the IC_50_ values determined for each drug from the MTT assay across the cell lines, with values reported as the mean ± SD from 2 independent experiments.

| Study | Drug | SUM149 | SUM159 | MDA-MB-231 | MCF-7 |
| --- | --- | --- | --- | --- | --- |
|  |  | (IC_50_ µM) | (IC_50_ µM) | (IC_50_ µM) | (IC_50_ µM) |
| **LWAS ^1^** | Docetaxel | 0.0003 ± 2.3E-05 | 0.0009 | 0.002 ± 0.0009 | 0.002 |
|  | Paclitaxel | 0.002 ± 0.0001 | 0.004 ± 0.0008 | 0.007 ± 0.002 | 0.003 |
|  | Vincristine | 0.003 ± 0.001 | 0.005 ± 0.0009 | 0.004 ± 0.003 | 0.009 |
|  | Vinorelbine | 0.2 ± 0.4 | 0.2 | 0.2 ± 0.03 | 0.1 |
|  | Lapatinib | 0.7 ± 0.5 | 3.6 ± 1.2 | 7.3 | 5.6 |
|  | Sunitinib | 2.1 ± 0.05 | 1.8 ± 0.3 | 2.1 ± 0.6 | 2.04 |
|  | Gefitinib | 0.1 ± 0.02 | 11 ± 1.9 | -- | -- |
|  | Doxorubicin | 0.3 ± 0.1 | 0.07 | 0.3 ± 0.1 | 0.2 |
|  | Daunorubicin | 0.09 ± 0.01 | 0.05 ± 0.003 | 0.2 ± 0.08 | 0.1 |
|  | Mitoxantrone | 0.08 ± 0.04 | 0.07 | 0.1 ± 0.05 | 0.05 |
|  | Etoposide | 0.2 ± 0.1 | 0.35 | 7.4 | 0.7 |
|  | Topotecan | 0.2 ± 0.2 | 0.02 | 0.8 ± 0.08 | 0.5 |
|  | Gemcitabine | 0.002 ± 0.0003 | 0.004 ± 0.0004 | 0.3 ± 0.03 | 0.01 |
|  | Cytarabine | 0.04 ± 0.004 | 0.05 | 0.8 ± 0.2 | 0.4 |
|  | Carboplatin | 4.5 ± 2.2 | -- | -- | -- |
|  | Methotrexate | 0.07 ± 0.09 | 0.1 | 0.5 ± 0.1 | 0.05 |
|  | 5-Fluorouracil | 1.1 | 15.7 ± 3.8 | 1.9 | 2.5 |
| **GRR ^2^** | BMS-536924 | 1.5 ± 0.1 | 1.2 ± 0.2 | 6.7 | 0.8 ± 0.1 |
|  | BMS-754807 | 3.6 ± 0.9 | 2.3 ± 0.7 | 8.0 | 0.2 ± 0.1 |
|  | AKTIV | 0.6 ± 0.2 | 0.7 ± 0.2 | 0.3 | 0.4 ± 0.1 |
|  | Temsirolimus | 7.3 ± 1.7 | 0.0005 ± 0.0005 | 0.0003 | 0.0005 ± 0.0004 |
|  | Tipifarnib | 1.3 ± 0.1 | 2.1 ± 0.7 | 6.4 | 3.8 ± 4.8 |
|  | Tyrphostin AG-1478 | 0.4 ± 0.2 | 5.7 ± 0.3 | 10.9 | -- |

^1^ Drugs identified by LWAS in Ji et al., 2020; ^2^ Drugs identified by GRR in Ji et al., 2023.
