# Supplementary material for: From in silico prediction to experimental validation: Identification of drugs and novel synergistic combinations that inhibit growth of inflammatory breast cancer cells
Source: PLoS One. 2026 Sep 16;21(9):e0339543. doi: 10.1371/journal.pone.0339543 (PMC13581016; doi:10.1371/journal.pone.0339543)
Supplement: Supplementary file 9 [file pone.0339543.s013.docx]

**S4 Table.** **Drugs selected for combination studies.** Selected compounds from LWAS and GRR active lists for combination studies in SUM149, along with their corresponding EC_25_ values and the highest concentration used for the 8 x 8 matrix.

|  | **Compounds** | **IC_50_ Average**  **“Hoechst Assay”**  **(µM)** | **EC_25_**  **(µM)** | **Highest Conc. in**  $\boldsymbol{8\times8}$ **Matrix (µM)** |
| --- | --- | --- | --- | --- |
| **LWAS** | Doxorubicin | 0.01 | 0.004 | 1 |
|  | Mitoxantrone | 0.005 | -- | -- |
|  | Etoposide | 0.07 | 0.023 | 1 |
|  | Topotecan | 0.008 | 0.003 | 0.1 |
|  | Docetaxel | 0.0004 | 0.0002 | 0.01 |
|  | Vincristine | 0.004 | 0.002 | 0.1 |
|  | Carboplatin | 4.4 | 1.5 | 10 |
|  | Fluorouracil | 4.8 | -- | -- |
|  | Lapatinib | 0.63 | 0.12 | 10 |
|  | Gefitinib | 0.4 | 0.2 | 10 |
|  | Sunitinib | 1.4 | -- | -- |
|  | Gemcitabine | 0.0012 | -- | -- |
|  | Cytarabine | 0.025 | 0.008 | 1 |
| **GRR** | BMS 536924 | 1.9 | 0.7 | 10 |
|  | BMS 754807 | 0.5 | 0.2 | 10 |
|  | AKTIV | 0.22 | 0.1 | 10 |
|  | Temsirolimus | 4.3 | 1.5 | 10 |
|  | Tipifarnib | 0.075 | 0.03 | 10 |
|  | Tyrphostin AG-1478 | 0.23 | 0.1 | 10 |
